# *Staphylococcus aureus* Responds to the Central Metabolite Pyruvate To Regulate Virulence

**DOI:** 10.1128/mBio.02272-17

**Published:** 2018-01-23

**Authors:** Lamia Harper, Divya Balasubramanian, Elizabeth A. Ohneck, William E. Sause, Jessica Chapman, Bryan Mejia-Sosa, Tenzin Lhakhang, Adriana Heguy, Aristotelis Tsirigos, Beatrix Ueberheide, Jeffrey M. Boyd, Desmond S. Lun, Victor J. Torres

**Affiliations:** a Department of Microbiology, New York University School of Medicine, Alexandria Center for Life Science, New York, New York, USA; b Proteomics Resource Center, Office of Collaborative Science, NYU School of Medicine, New York, New York, USA; c Center for Computational and Integrative Biology and Department of Computer Science, Rutgers University, Camden, New Jersey, USA; d Applied Bioinformatics Center, Office of Collaborative Science, NYU School of Medicine, New York, New York, USA; e Genome Technology Center, Office of Collaborative Science, NYU School of Medicine, New York, New York, USA; f Department of Biochemistry and Microbiology, Rutgers University, New Brunswick, New Jersey, USA; University of Pittsburgh; University of Rochester

**Keywords:** *Staphylococcus aureus*, gene regulation, infection, metabolite, pathogenesis, pyruvate

## Abstract

*Staphylococcus aureus* is a versatile bacterial pathogen that can cause significant disease burden and mortality. Like other pathogens, *S. aureus* must adapt to its environment to produce virulence factors to survive the immune responses evoked by infection. Despite the importance of environmental signals for *S. aureus* pathogenicity, only a limited number of these signals have been investigated in detail for their ability to modulate virulence. Here we show that pyruvate, a central metabolite, causes alterations in the overall metabolic flux of *S. aureus* and enhances its pathogenicity. We demonstrate that pyruvate induces the production of virulence factors such as the pore-forming leucocidins and that this induction results in increased virulence of community-acquired methicillin-resistant *S. aureus* (CA-MRSA) clone USA300. Specifically, we show that an efficient “pyruvate response” requires the activation of *S. aureus* master regulators AgrAC and SaeRS as well as the ArlRS two-component system. Altogether, our report further establishes a strong relationship between metabolism and virulence and identifies pyruvate as a novel regulatory signal for the coordination of the *S. aureus* virulon through intricate regulatory networks.

## IMPORTANCE

Delineation of the influence of host-derived small molecules on the makeup of human pathogens is a growing field in understanding host-pathogen interactions. *S. aureus* is a prominent pathogen that colonizes up to one-third of the human population and can cause serious infections that result in mortality in ~15% of cases. Here, we show that pyruvate, a key nutrient and central metabolite, causes global changes to the metabolic flux of *S. aureus* and activates regulatory networks that allow significant increases in the production of leucocidins. These and other virulence factors are critical for *S. aureus* to infect diverse host niches, initiate infections, and effectively subvert host immune responses. Understanding how environmental signals, particularly ones that are essential to and prominent in the human host, affect virulence will allow us to better understand pathogenicity and consider more-targeted approaches to tackling the current *S. aureus* epidemic.

## INTRODUCTION

*Staphylococcus aureus* is a commensal Gram-positive bacterium that can also be considered a dangerous pathogen. While *S. aureus* asymptomatically colonizes ~30% of the population, it can also cause infections ranging from mildly invasive conditions like cutaneous abscesses and impetigo to much more severe invasive diseases such as endocarditis, osteomyelitis, and necrotizing fasciitis (1). Methicillin-resistant *S. aureus* (MRSA) is one of the leading causes of hospital-acquired infections in the United States, with up to 15% of severe infections leading to death (2). Interestingly, when *S. aureus* is cultured from sites of infection, it often derives from endogenous sites of colonization such as the skin or nares of the host, suggesting that colonizing bacteria become pathogenic (3). The precise mechanism that facilitates this switch from a commensal to pathogenic lifestyle, however, is not clear.

The classic model for an epidemiological triangle (host-pathogen-environment) posits that the progress and severity of an infection is influenced by a combination of factors, including the immune system of the host, virulence of the pathogen, and the environmental conditions governing the infection (4). Delineating the role of each of these factors, particularly the contribution of the environment, would allow us to better understand the progression of infection. *S. aureus* produces an array of virulence factors that allows the bacterium to colonize the host as well as to initiate and maintain an infection (5). These factors include exotoxins, exoenzymes, surface proteins, and immunomodulatory proteins. One group of exotoxins consists of the cytolytic bicomponent pore-forming leucocidins (referred to here as leucocidins), which form octameric pores on select immune cells and cause death via osmotic lysis (6). Extensive studies performed in our laboratory and in those of others have shown that leucocidins are important in *S. aureus* pathogenesis (6). Therefore, defining the environmental regulation of such proteins is critical to understanding *S. aureus* pathogenesis.

Given its wide spectrum for routes of infection, *S. aureus* must be able to adapt to diverse and often hostile host environments by sensing environmental signals and modulating the expression of virulence factors. Recent reviews have highlighted regulatory systems that sense environmental changes in bacterial density, nutrient and metabolite levels, oxygen levels, pH levels, production of reactive oxygen species, and other external cues (7, 8). Bacteria rely heavily on two-component systems (TCSs) to sense such changes (9). TCSs are composed of a sensor kinase that detects an environmental signal and initiates a phosphorelay cascade and a response regulator that is activated by the phosphorelay event to modulate the expression of target genes. In *S. aureus*, one of the best-characterized TCSs is the accessory gene regulator (Agr) quorum sensing system (10), which responds to changes in bacterial cell density to regulate gene expression (11), including that of the regulatory effector RNA known as RNAIII. RNAIII inhibits the translation of the repressor of toxin (Rot) protein (12, 13), which alters the expression of many virulence factors (14). An additional regulatory system that is critical to *S. aureus* host adaptation is the *S. aureus* exoprotein (Sae) TCS (15). In response to low pH, changes in cellular respiration, high concentrations of sodium chloride, neutrophils and their contents, or subinhibitory levels of select antibiotics (16–19), the SaeS sensor kinase activates the SaeR response regulator, which directly modulates virulence gene expression. In the context of virulence factor regulation, particularly for the leucocidins, SaeR serves as the master activator of toxin expression. The coordinated inhibition of Rot synthesis by the Agr TCS, as well as the activation of the Sae TCS, is required for maximal toxin expression and production.

While gene regulation in *S. aureus* has been extensively studied under *in vitro* conditions, the impact of host environments and host-derived signals on the regulation of virulence factors is less well studied. Environmental signals and the sensors that are responsible for responding to them could be critical for *S. aureus* pathogenicity and may also play a role in mediating the switch from commensal to pathogen. In this study, we set out to investigate the mechanism by which *S. aureus* coordinates the altered expression of leucocidins in a growth medium-dependent manner. We establish that pyruvate, an additive commonly used in the media CCY (casein hydrolysate/yeast extract) and YCP (yeast extract/Casamino Acids/pyruvate), is a regulatory signal for *S. aureus* pathogenicity. The identification of pyruvate as a molecule that can alter *S. aureus* virulence is significant because pyruvate is a critical metabolite that drives ATP production through central metabolism. Using a combination of transcriptomics, proteomics, and *in silico* metabolomics, we show that pyruvate causes global changes in metabolic flux and alters the transcriptional profile of virulence genes, resulting in increased production of secreted proteins. Our data suggest that pyruvate acts by modulating the activity of the Agr TCS to keep the levels of Rot low, allowing increased toxin expression. Moreover, we identified the ArlRS TCS (20) as a component that is critical for the pyruvate-mediated response in *S. aureus*. We also establish the physiological relevance of this metabolite, demonstrating that pyruvate enhances *S. aureus* pathogenicity.

## RESULTS

### Pyruvate causes global changes in the exoproteome of *S. aureus*.

**FIG 1:**
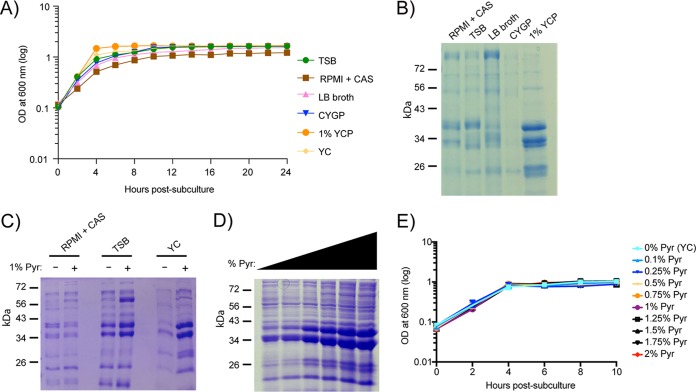
Pyruvate affects production of exoproteins in a dose-dependent manner. (A and B) USA300 (LAC) was used to assess growth curves (A) and exoprotein profiles (B) under 6 different media conditions (see Table 1). (C and D) The exoprotein profiles of LAC in RPMI + CAS, TSB, or YC media with or without 1% pyruvate (Pyr) (C) or in YC media supplemented with 0% to 2% pyruvate (D). (E) The growth curve of LAC in 0 to 2% pyruvate. For the exoprotein profiles, bacteria were grown to post-exponential phase (5 h), and the proteins were precipitated from cultured supernatants, separated using SDS-PAGE, and stained with Coomassie. Representative gels are shown. The growth curves were generated using three independent colonies grown in a 96-well format. Errors bars denote the standard deviations.

Previous work had demonstrated that alterations in *S. aureus* growth conditions can affect the regulation of leucocidins (21, 22), which directly contributes to the bacterium’s pathogenicity (23–26). In an attempt to determine what medium components may lead to this differential regulation, we investigated changes in the exoprotein profile of community-acquired methicillin-resistant *S. aureus* (CA-MRSA) USA300 (strain LAC; referred to here as USA300), the predominant CA-MRSA linage in the United States (27), when grown in various laboratory media (Fig. 1). While the growth kinetics of USA300 were similar in all of the media tested (Fig. 1A), the exoprotein profiles varied, particularly for YCP, which induced robust production of exoproteins compared to the other media tested (Fig. 1B). Upon examining the composition of each media, we noticed that pyruvate was unique to YCP (Table 1). To directly evaluate the effect of pyruvate on the production of exoproteins by USA300, we exogenously added pyruvate to YC, TSB, and RPMI + CAS. We observed that pyruvate induced the production of exoproteins in complex media such as TSB and YC, but this effect was not observed in RPMI + CAS, which is a more minimal media. We observed that the effect of pyruvate on the exoproteome in YC media was dose dependent (Fig. 1D) without affecting bacterial growth (Fig. 1E).

**TABLE 1:** General composition of growth media

Acronym	Name	Major components (general)
TSB	Tryptic soy broth	Tryptone, soytone, glucose, salts
		
RPMI + CAS	RPMI 1640	Amino acids, vitamins, glucose, glutathione, 1% Casamino Acids, sodium bicarbonate, salts
		
LB	Luria-Bertani broth	Typtone, yeast extract, salts
		
CYGP	Casamino Acids/yeast extract/β-glycerophosphate	Casamino Acids, yeast extract, β-glycerophosphate, salts
		
YCP	Yeast extract/Casamino Acids/pyruvate	Yeast extract, Casamino Acids, pyruvate, salts

To delineate whether the observed effect of pyruvate was an indirect result caused by access to an additional nutrient source, we cultured USA300 in YC media supplemented with pyruvate or another metabolite (e.g., glucose, lactate, acetate, or glutamate), all of which were controlled with equal molar concentrations of carbon. While no major growth differences were observed with the metabolites (see Fig. S1A in the supplemental material), pyruvate caused the most pronounced induction of exoproteins (Fig. S1B).

10.1128/mBio.02272-17.1 FIG S1  Pyruvate has the most pronounced effect of key central metabolites on the induction of the exoproteome. USA300 cultured in YC media supplemented with glucose, pyruvate, lactate, acetate, or glutamate normalized to equal molar concentrations of carbon corresponding to 2% pyruvate was used to assess the growth curve (A) and the exoprotein profile at the post-exponential-growth phase (B). Download FIG S1, PDF file, 0.5 MB. Copyright © 2018 Harper et al. 2018 Harper et al. This content is distributed under the terms of the Creative Commons Attribution 4.0 International license.

### Pyruvate alters the production of virulence factors.

**FIG 2:**
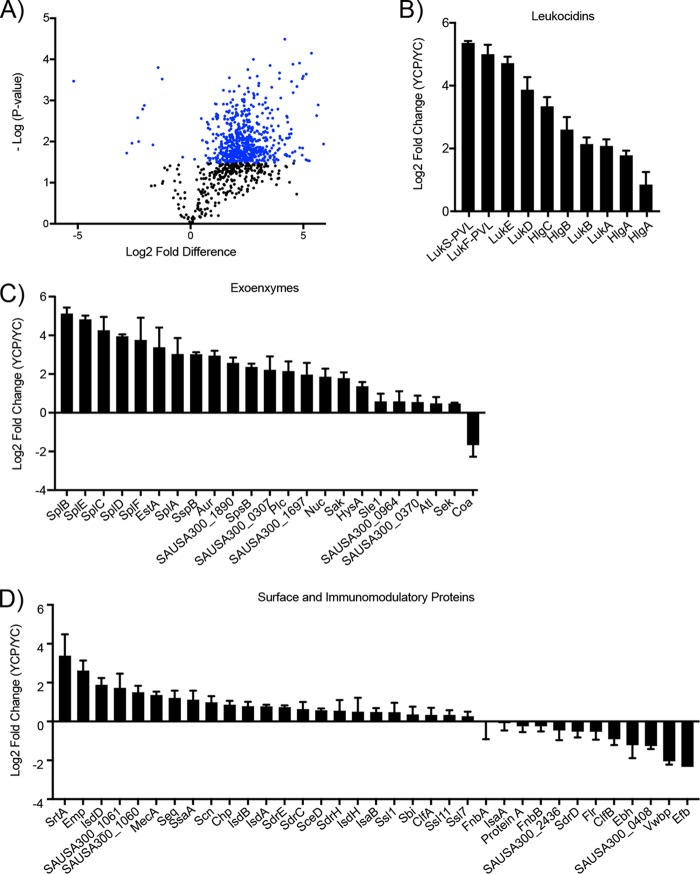
Pyruvate causes major changes in production of surface and secreted proteins, including known virulence factors. Surface and secreted proteins from USA300 grown to post-exponential phase (5 h), in the absence (YC) or presence (YCP) of 2% pyruvate, were assessed by quantitative mass spectrometry. (A) Volcano plot. The blue points depict proteins that were significantly changed in the presence of pyruvate. (B to D) Relative fold change in the levels of leucocidins (B), exoenzymes (C), and surface and immunomodulatory proteins (D). Values represent average relative fold changes in three independent cultures ± standard deviations.

To better understand how pyruvate induces the production of exoproteins, we performed quantitative mass spectrometry (MS) on surface and secreted proteins collected from USA300 grown to the post-exponential-growth phase, in the presence or absence of pyruvate. Pyruvate caused significant changes in the abundance of membrane-associated and secreted proteins (Fig. 2A; see also Table S1 in the supplemental material). An analysis of the relative fold change data revealed that many known virulence factors were among those most affected by pyruvate (Fig. 2).

10.1128/mBio.02272-17.4 TABLE S1  Proteins that significantly changed in abundance in the presence of pyruvate. Download TABLE S1, DOCX file, 0.1 MB. Copyright © 2018 Harper et al. 2018 Harper et al. This content is distributed under the terms of the Creative Commons Attribution 4.0 International license.

In *S. aureus*, the production and secretion of virulence factors are controlled in a growth-phase-dependent manner via the Agr TCS (10). During the exponential-growth phase, presumably corresponding to the time when an infection is first being established, the levels of immunomodulatory proteins are thought to be high whereas those of cytotoxins are low. At the post-exponential and early stationary phases, mimicking the stage when *S. aureus* has successfully established infection, the levels of surface and immunomodulatory proteins are thought to be reduced while cytotoxin production is increased (10). This trend at the post-exponential phase not only is maintained in the presence of pyruvate but is exacerbated relative to the no-pyruvate control. Expression of immunomodulatory and surface proteins tends to be relatively unchanged (fold change of <1.5) or repressed, whereas expression of exoenzymes and leucocidins is induced up to 6-fold (Fig. 2B to D).

**FIG 3:**
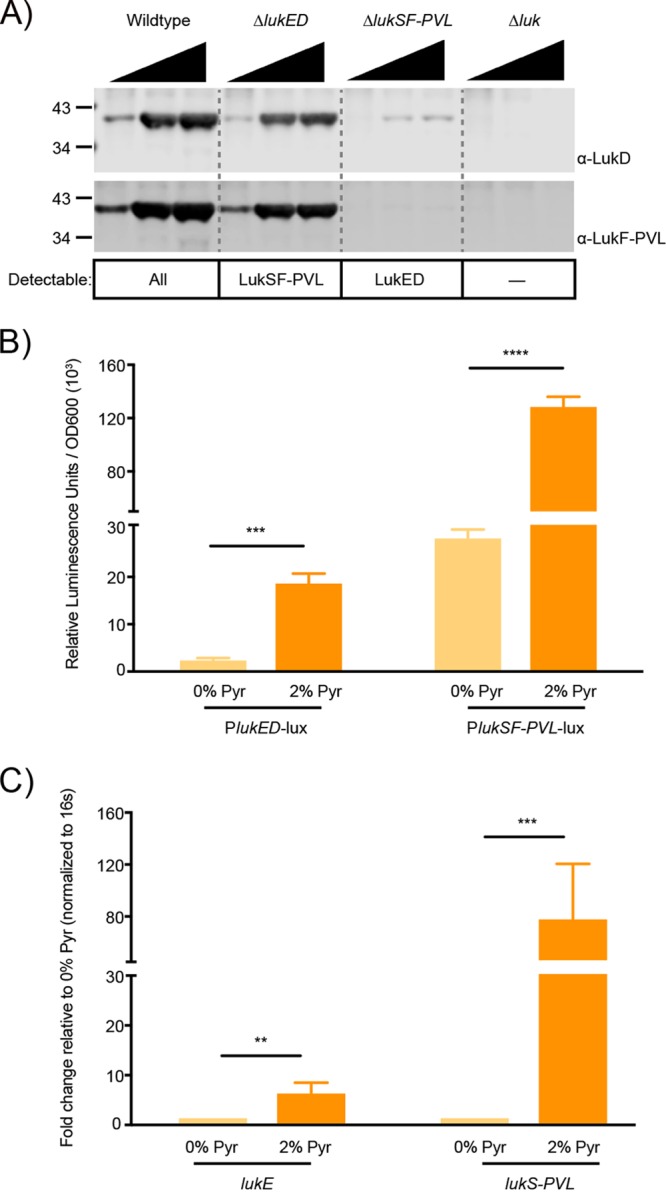
Pyruvate enhances the production of leucocidins LukED and LukSF-PVL at the level of transcription. (A) Secreted proteins were precipitated from supernatants of wild-type or leucocidin-deficient USA300 strains that were grown in the presence of 0%, 1%, or 2% pyruvate. Leucocidins were detected using polyclonal antibodies against LukD or LukF. The Δ*lukED and* Δ*lukSF-PVL* strains also lacked *hlgACB* to prevent cross-reactivity with the anti-Luk antibodies. The leucocidins that should still be detectable given the genetic background of the strain used are listed below. *Δluk* = *ΔhlgACB ΔlukED ΔlukSF-PVL ΔlukAB*. (B and C) The activity and expression of wild-type USA300 genes encoding LukED and LukSF-PVL were examined in the absence or presence of 2% pyruvate. Analysis of promoter reporter strains driving the expression of a luciferase gene from the *lukED* or *lukSF-PVL* promoters (B) and RT-qPCR analysis of *lukE* and *lukS-PVL* transcripts (C) were performed. The average fold change seen with medium plus 2% pyruvate was determined based on the mRNA of the target genes normalized to endogenous 16S ribosomal RNA relative to 0% pyruvate. Values represent average relative luminescence levels or fold change seen in 3 independent experiments ± standard deviations. Analysis of statistical significance was performed using a Student’s *t* test. *, *P* < 0.05; **, *P* < 0.01; ***, *P* < 0.005; ****, *P* < 0.0001.

As LukED and LukSF-PVL were among the most highly induced proteins in the proteomics analysis, we sought to further characterize pyruvate’s effect on virulence factor production using these proteins as a reference. We first aimed to validate the mass spectrometry findings by performing immunoblots using proteins secreted by USA300. As commonly used anti-leucocidin antibodies exhibit cross-reactivity due to the high (up to 70%) amino acid identity of the leucocidins (6), we used isogenic mutant strains to distinguish between induction of LukED and induction of LukSF-PVL. As observed in Fig. 3A, pyruvate indeed induced the production of both LukD and LukF, albeit with lower overall abundance for LukD.

Furthermore, measuring the activity of the leucocidin promoters, we observed that pyruvate induced a significant increase in the activity of both the *lukED* and *lukSF-PVL* promoters (Fig. 3B), which in turn resulted in an 8-fold increase in the transcript levels of *lukE* and an 80-fold increase in the transcript levels of *lukS-PVL* (Fig. 3C). Given the importance of master regulators in regulating leucocidin expression, we sought to test the hypothesis that pyruvate affects toxins by activating the expression of either *agr* or *sae*, both of which encode well-characterized toxin regulators (28). Using transcriptional reporters, we observed that pyruvate indeed stimulates the activity of both the *agr* and *sae* promoters (Fig. S2A to B).

10.1128/mBio.02272-17.2 FIG S2  Pyruvate activates the promoters of master regulators *agr* and *sae*. (A and B) The promoter activity of *agr* and *sae* in USA300 was examined in the absence or presence of 2% pyruvate using promoter reporter strains driving the expression of GFP from the *agrP3* (A) or *sae* (B) promoter over time. Values representing the average levels of fluorescence seen in 3 independent experiments are shown ± standard deviations. Analysis of statistical significance was performed using a Student’s *t* test. ***, *P* < 0.005; ****, *P* < 0.0001. Download FIG S2, PDF file, 0.1 MB. Copyright © 2018 Harper et al. 2018 Harper et al. This content is distributed under the terms of the Creative Commons Attribution 4.0 International license.

As changes in pH have been previously shown to influence the expression of virulence factors in unbuffered media (29), we investigated whether changes in pH could account for increased production of the leucocidins in the presence of pyruvate. While the pH of YC does drop faster than that of YCP, when both media were buffered to pH ~5 to ~6 or pH ~6 to ~7, pyruvate was still able to induce the production of the leucocidins (Fig. S3).

10.1128/mBio.02272-17.3 FIG S3  The pH of the culture does not affect pyruvate-mediated leucocidin production. A representative Western blot of F-type leucocidins (Luk) is shown for protein isolated from the cultured supernatants of USA300 at the post-exponential-growth phase. YC medium with or without pyruvate was tested without a buffer or was buffered to either pH ~7.2 in HEPES or pH ~5.4 in MES. The pH levels seen in 3 independent experiments were measured at the start of the culture and at the post-exponential-growth phase (5 h). The pH values are shown ± standard deviations. Download FIG S3, PDF file, 0.1 MB. Copyright © 2018 Harper et al. 2018 Harper et al. This content is distributed under the terms of the Creative Commons Attribution 4.0 International license.

### Pyruvate influences the transcriptome of *S. aureus* and alters metabolism.

**FIG 4:**
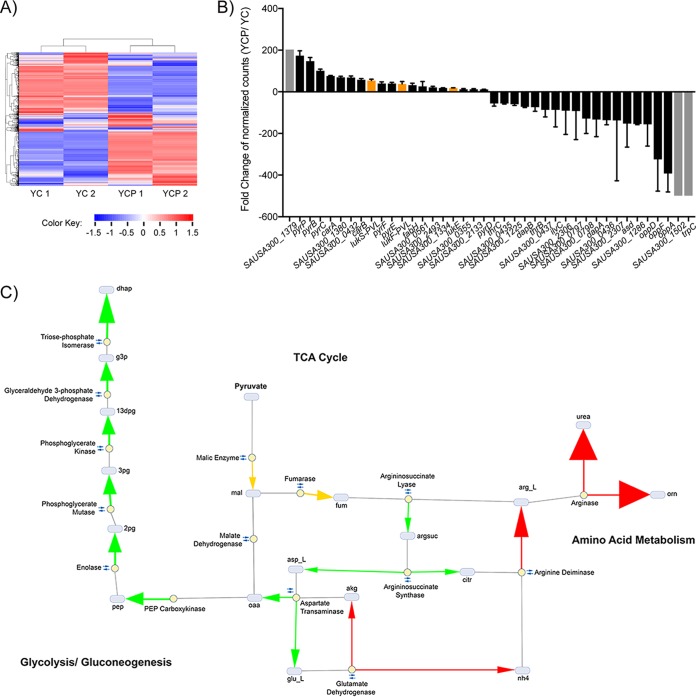
Pyruvate causes marked changes to the transcriptome of *S. aureus*, leading to alterations in the metabolic flux. (A) Heat map of all transcripts detected in RNA extracted from USA300 grown to early stationary phase, in the absence (YC) or presence (YCP) of 2% pyruvate. (B) The relative fold change of the genes most induced or repressed by pyruvate, as detected by RNA-Seq. Genes with normalized counts that were absent in both replicates under one set of conditions but significantly present in each of the two replicates under the other set of conditions are denoted with a gray bar and set at the maximum fold change. Orange bars highlight the leucocidins. Values represent average relative fold changes ± standard deviations seen in two independent colonies. (C) Simplified graphical representation of pyruvate-induced fold changes in metabolic flux of USA300. Grey ovals represent metabolic intermediates and yellow dots represent the enzymes involved in catalyzing reactions. Small double arrows represent reversible reactions. The magnitude of the fold flux is denoted by arrowhead thickness. The color of the arrow signifies relative flux activity levels in YCP versus YC as follows: green arrows indicate increased activity, red arrows indicate decreased activity, and orange arrows indicate that the enzyme activity switched direction. Abbreviations are as follows: 2 pg, 2-phosphoglycerate; 3 pg, 3-phospho-d-glycerate; 13dpg, 3-phospho-d-glyceroyl phosphate; akg, α-ketoglutarate; arg_L, l-arginine; argsuc, argininosuccinate; asp_L, l-aspartate; citr, citrulline; dhap, dihydroxyacetone phosphate; fum, fumarate; g3p, glyceraldehyde 3-phosphate; glu_L, l-glutamate; mal, malate; nh_4_, ammonium; oaa, oxaloacetate; orn, ornithine; pep, phosphoenolpyruvate.

To determine the extent to which pyruvate alters the global transcriptome of *S. aureus*, we performed RNA sequencing (RNA-Seq) (30). Pyruvate triggered marked alterations in USA300 gene expression (Fig. 4A; see also Tables S2 and S3). Of the 103 genes with fold expression changes greater than 10, those encoding leucocidins were among the most strongly induced, whereas genes encoding surface and immunomodulatory proteins were among the most highly repressed (Fig. 4B; Tables S2 and S3). These results are congruent with the proteomics data representing changes in the relative levels of abundance of virulence factors (Fig. 2C and D).

10.1128/mBio.02272-17.5 TABLE S2  Genes that significantly changed in relative mRNA levels in the presence of pyruvate. Download TABLE S2, DOCX file, 0.2 MB. Copyright © 2018 Harper et al. 2018 Harper et al. This content is distributed under the terms of the Creative Commons Attribution 4.0 International license.

10.1128/mBio.02272-17.6 TABLE S3  Genes most induced in the presence of pyruvate. Download TABLE S3, DOCX file, 0.03 MB. Copyright © 2018 Harper et al. 2018 Harper et al. This content is distributed under the terms of the Creative Commons Attribution 4.0 International license.

As expected, genes involved in metabolism and transport were categorically the most significantly affected by pyruvate (Fig. 4B; see also Table S4). As changes in metabolic flux have been shown to influence *S. aureus* pathogenicity (7, 8, 31), we were interested in identifying trends in key metabolic pathways that might contribute to pyruvate’s effect on toxin regulation. To gain insight into the potential impact that pyruvate supplementation had on USA300 metabolism, we performed *in silico* metabolic profiling analyses (32) of the transcriptomic data (Fig. 4C). An important aspect of metabolic flux analysis is defining the carbon sources available to the bacteria. Carbon metabolism is not limited to a single substrate but may switch to alternative substrates when the preferential carbon source becomes available (33). For example, in the absence of glucose, *S. aureus* uses amino acids as its carbon source. When glucose is available, however, amino acids become secondary sources, as glucose is preferentially utilized by the cell (34). In our E-Flux2 simulations, we observed a similar response in the flux profile of pyruvate-supplemented USA300. The predicted uptake flux of amino acids such as serine, threonine, glutamate, and arginine decreased or became negligible under YCP growth conditions, as pyruvate influx accounted for the bulk of incoming carbon flux. The apparent shift from amino acids to pyruvate as a primary carbon source resulted in the differential responses of three metabolic pathways: glycolysis/gluconeogenesis, tricarboxylic acid (TCA) cycle, and amino acid metabolism (Fig. 4C; see also Table S4).

10.1128/mBio.02272-17.7 TABLE S4  Metabolic pathways whose flux was significantly altered in the presence of pyruvate. Download TABLE S4, DOCX file, 0.1 MB. Copyright © 2018 Harper et al. 2018 Harper et al. This content is distributed under the terms of the Creative Commons Attribution 4.0 International license.

First, we observed an increase in the levels of transcripts involved in gluconeogenesis in USA300 grown in the presence of pyruvate. For both YC and YCP simulations, the entry point into the gluconeogenesis pathway is phosphoenolpyruvate, whose biosynthesis is controlled by flux across the reaction catalyzed by phosphoenolpyruvate carboxykinase. In the absence of pyruvate, flux toward oxaloacetate biosynthesis was found to represent a combination of arginine influx into the metabolic network and flux through malate dehydrogenase. In the presence of pyruvate, however, despite an ~15.6-fold decrease in arginine uptake, subsequent pyruvate influx into malate through malic enzyme resulted in an ~4.5-fold increase in flux through enolase and in up to a 5-fold flux increase across subsequent upstream gluconeogenesis reactions (Fig. 4C; see also Table S4).

Second, the simulation predicted that two reversible reactions in the TCA cycle would switch direction in the presence of pyruvate, shifting overall metabolic flux away from pyruvate biosynthesis. This was expected, as there is no need to expend cellular resources to synthesize a metabolite that is readily available to the bacterium. These reactions were catalyzed by malic enzyme and fumerase, two enzymes that upon being simultaneously reversed, result in an overall flux shift towards fumarate biosynthesis. There is a concomitant increase in flux activity across reactions in aspartate and glutamate metabolism, which ultimately feeds back into oxaloacetate biosynthesis, serving as an anaplerotic source for replenishing gluconeogenesis intermediates.

Third, we observed a decrease in amino acid deamination and, consequently, a decrease in ammonia and urea production. As pyruvate is preferentially utilized by the bacteria under these conditions, amino acid transport into the cells decreased, resulting in a decrease in amino acid catabolism. In particular, with respect to arginine metabolism, we observed flux fold decreases of between 4 and 6 across reactions leading up to urea excretion, with the arginase-catalyzed reaction showing a notable ~33-fold decrease in activity in pyruvate. These metabolic flux data are in line with literature linking carbohydrate-dependent suppression of ammonia generation to virulence in *S. aureus* (31).

### The ArlRS two-component system is necessary for pyruvate-mediated regulation of toxin production.

**FIG 5:**
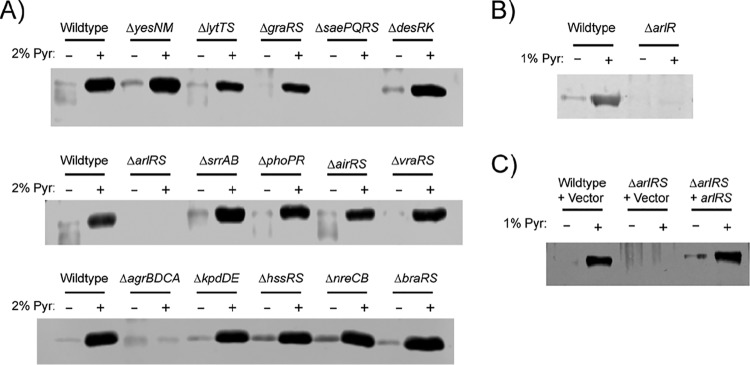
The ArlRS TCS is involved in pyruvate-mediated leucocidin regulation. Representative Western blots of F-type leucocidins isolated from cultured supernatants of USA300 strains grown to the post-exponential-growth phase in 0% or 2% pyruvate are shown. (A) USA300 wild-type strain or isogenic mutants lacking the indicated two-component systems. (B) USA300 strain AH-LAC, wild-type strain, or transposon mutant with a mutation of the gene encoding the ArlR response regulator. (C) USA300 wild-type strain or Δ*arlRS* mutant, with chromosomal integration of the empty pJC1306 vector or pJC1306 *arlRS*-positive (*arlRS*^+^) complementation plasmid.

Given pyruvate’s activity in regulating virulence (Fig. 2 and 3), we were interested in gaining insight into how USA300 may sense pyruvate. We screened a collection of USA300 isogenic mutants lacking all the nonessential TCSs to determine whether any of these may be involved in sensing or responding to pyruvate and in transducing the signal that ultimately leads to increased toxin production. By monitoring leucocidin protein levels, we found that deletion of master regulators *agr* and *sae* blunted pyruvate-mediated induction of leucocidins (Fig. 5A), consistent with the role of these regulators in the production of toxins (28). Interestingly, we found that deletion of the *arlRS* TCS also resulted in attenuated induction of leucocidin production by pyruvate (Fig. 5A).

The ArlRS TCS is a regulator of virulence (35–38). Additionally, microarray data indicate that ArlRS regulates genes involved in sugar uptake and enzymes involved in amino acid utilization (39) and mediates alterations in the utilization of carbon sources (38). Taken together, these observations suggest that the ArlRS TCS may be important in regulating *S. aureus* metabolism by controlling some of the same metabolic pathways that pyruvate is predicted to affect. While we understand that the ArlRS TCS is important for *S. aureus* virulence and has been implicated in metabolic processes, the signal(s) that activates this TCS is unknown. Thus, we decided to further characterize the connection between pyruvate and the ArlRS TCS.

We first sought to verify the findings of the screen showing that leucocidin induction is attenuated in a USA300 strain lacking *arlRS* (Δ*arlRS*). To this end, we used a transposon insertion mutant of *arlR* (40). We observed that disruption of the *arlR* response regulator resulted in attenuated basal levels of leucocidins, which we would expect, as ArlRS is a positive regulator of virulence. Moreover, in the absence of functional ArlR, pyruvate failed to induce toxin production (Fig. 5B). Finally, when a wild-type copy of the *arlRS* locus was reintroduced into the chromosome of the Δ*arlRS* mutant, pyruvate-mediated induction of leucocidins was restored (Fig. 5C), confirming that the observed effect was indeed ArlRS specific.

**FIG 6:**
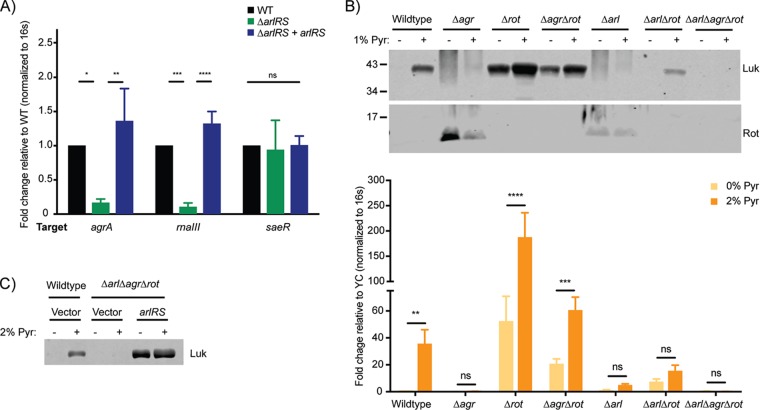
Pyruvate acts through Arl and Agr to suppress Rot and activate leucocidin expression. USA300 strains were grown to post-exponential phase. (A) RT-qPCR analysis of transcript levels in medium with 0% pyruvate in WT, *ΔarlRS*, or *ΔarlRS*-plus-*arlRS* complementation strains. The fold change seen with the target gene in the *ΔarlRS* mutant or complemented strains is normalized to the endogenous 16S ribosomal RNA, relative to the wild-type control. (B and C) Western blots detecting F-type leucocidins (LukS) isolated from cultured supernatants (top) or Rot from cytoplasmic lysate (bottom) (B) or RT-qPCR analyses of *lukS* transcripts (C) using mutants harboring deletions of genes encoding Agr, Rot, or ArlRS, either individually or in combination. The fold change of *lukS-PVL* in 2% pyruvate was normalized to endogenous 16S ribosomal RNA, relative to 0% pyruvate. For RT-qPCR, the relative fold change values represent averages from 3 to 5 independent experiments ± standard deviations. The analyses of statistical significance were performed using an ANOVA, correcting for multiple comparisons using Dunnett’s analysis. *, *P* < 0.05; **, *P* < 0.01; ***, *P* < 0.005; ****, *P* < 0.0001; ns, not significant.

Given that the Agr and Sae TCSs also seem to be involved in the pyruvate response, we performed reverse transcriptase quantitative PCR (RT-qPCR) to determine whether ArlRS affects the expression of the *agr* locus or the *sae* locus. As shown in Fig. 6A, *agrA* and RNAIII gene levels were significantly reduced in an Δ*arlRS* mutant, confirming previous reports that the ArlRS TCS positively regulates *agr* (39). However, loss of *arlRS* had no detectable effect on basal *sae* expression (Fig. 6A), suggesting that pyruvate can activate the Sae TCS independently of ArlRS (Fig. 6B).

We next aimed to delineate the mechanism for pyruvate-mediated induction of toxins, looking specifically at the interplay between Arl and Agr. In wild-type USA300, pyruvate induced robust leucocidin production (Fig. 2, 3, and 6B and C). Deletion of the *agr* locus resulted in increased Rot production and decreased leucocidin production, even in the presence of pyruvate (Fig. 6B). In the absence of Rot, leucocidin expression and production were increased and were further heightened by the presence of pyruvate. While production of leucocidins was slightly lower in an Δ*agr* Δ*rot* double mutant than in the Δ*rot* mutant, leucocidins were still induced by pyruvate (relative to the Δ*agr* mutant results) based on transcript and protein levels (Fig. 6B; top and bottom), suggesting that pyruvate can regulate toxin production independently of Agr. These data also suggest that pyruvate failed to induce toxin expression in a Δ*agr* mutant due to high levels of Rot, whose repression of toxin expression may be too strong for even pyruvate to overcome.

Deletion of *arlRS* phenocopied the Δ*agr* mutant, resulting in increased production of Rot (relative to the wild-type strain), and impaired pyruvate-mediated toxin induction (Fig. 6B [top and bottom]). However, whereas pyruvate was still able to cause major induction of toxins in the Δ*agr* Δ*rot* mutant, there was only minor pyruvate-mediated induction in the Δ*arlRS* Δ*rot* mutant. This can be accounted for by the presence of the Agr TCS, as deletion of *arlRS*, *agr*, and *rot* together resulted in no leucocidin induction by pyruvate (Fig. 6B and C). Thus, ArlRS TCS responds to pyruvate in both Agr-dependent and -independent fashions.

### Pyruvate regulates *S. aureus* pathogenicity.

**FIG 7:**
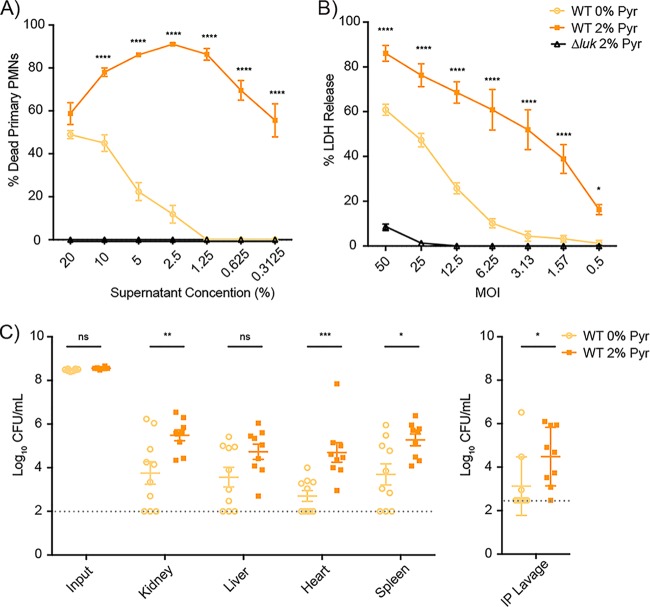
Pyruvate enhances the virulence of USA300. (A and B) Wild-type and isogenic leucocidin-inactivated (*Δluk*) USA300 strains were cultured to post-exponential phase in 0% or 2% pyruvate and used to determine the impact of virulence *ex vivo*. (A) Primary human PMNs (2 × 10^5^ cells/well) were either incubated with filtered culture supernatants to a concentration of 0.313% to 20% with CellTiter metabolic dye or were infected with live USA300 strains at a multiplicity of infection (MOI) of 0.5 to 50 for intoxication assays measuring cell death levels. (B) Cell death was measured as the percentage of LDH release from lysed neutrophils. For both experiments, values representing "% Dead Primary PMNs” represent averages from neutrophils isolated from 4 to 5 independent donors, each incubated/infected with two USA300 colonies, ± standard errors of the means. The analyses of statistical significance were performed using a two-way ANOVA, correcting for multiple comparison using Dunnett’s analysis. Stars denote the statistical significance of results of comparisons of wild-type LAC in the presence or absence of pyruvate. *Δluk* = *ΔhlgACB ΔlukED ΔlukSF-PVL ΔlukAB*. (C) Mice (in two independent experiments conducted with a total of 10 mice per condition) were challenged via intraperitoneal infection with 0.5 × 10^8^ to 1 × 10^8^ CFU of wild-type USA300 LAC cultured to post-exponential phase in 0% or 2% pyruvate. At approximately 42 h postinfection (p.i.), mice were sacrificed and bacteria were recovered from the indicated organs to determine bacterial burden. Values representing the input or average CFU level per milliliter of bacteria per organ are shown ± standard errors of the means. The dotted line signifies the limit of detection for CFU. Analysis of statistical significance for bacterial burden was performed using a Student’s *t* test. *, *P* < 0.05; **, *P* < 0.01; ***, *P* < 0.005; ****, *P* < 0.0001.

To determine if pyruvate alters the *S. aureus*-host interaction, we exposed primary human polymorphonuclear neutrophils (PMNs), representing an innate immune cell critical for the containment of *S. aureus* (41) and a target of leucocidins (6), to increasing concentrations of bacterium-free culture filtrates (i.e., exoproteins) isolated from USA300 grown with or without pyruvate supplementation. The exoproteins from bacteria grown with pyruvate exhibited a cytotoxic effect on PMNs that was significantly higher and more sustained than that seen with the exoproteins isolated from bacteria grown without pyruvate (Fig. 7A).

We next investigated if the observed enhancement of pyruvate-mediated cytotoxicity by the exoproteome was also observable when PMNs were infected with live bacteria that had been previously exposed to pyruvate. To this end, primary human PMNs (hPMNs) were infected with USA300 grown in 0% or 2% pyruvate, washed, and then normalized to different multiplicities of infection (MOIs). Release of host lactate dehydrogenase (LDH) was used to measure bacterium-mediated lytic activity. As with the exoproteins, we observed a drastic increase in LDH release from PMNs infected with USA300 grown in pyruvate, even after the bacteria had been washed (Fig. 7B). Importantly, the pyruvate-mediated increase in cytotoxic activity toward PMNs, using both bacterium-free cultured supernatant and live bacteria, was the result of increased production of bicomponent leucocidins, as an isogenic mutant lacking these toxins failed to kill PMNs even at the highest concentrations and MOIs tested (Fig. 7A and B).

To further understand the potential role of pyruvate in *S. aureus* pathogenesis *in vivo*, we used a murine model of systemic intraperitoneal infection. Mice were challenged with USA300 grown in YC supplemented with or not supplemented with 2% pyruvate, and the bacterial burden was monitored 42 h postinfection. While the bacterial input levels were comparable in the two groups, the mice challenged with USA300 cultured in pyruvate had significantly higher bacterial burdens in the kidneys, heart, spleen, and peritoneal cavity (Fig. 7C). Taken together, these data demonstrate that exposure to pyruvate can enhance the severity of *S. aureus* pathogenesis.

## DISCUSSION

*S. aureus* is a human pathogen that is prominent both in the community and in nosocomial settings (1). The effectiveness of the bacterium is due, in part, to its ability to readily adapt to diverse environmental conditions and produce a wide array of virulence factors to facilitate host colonization and immune evasion (5). In this study, we sought to understand how different growth conditions affect the production of exoproteins by *S. aureus*. In doing so, we identified pyruvate as an important signal involved in the regulation of secreted proteins (Fig. 1). We demonstrate that pyruvate causes global changes to the exoproteome of USA300 and that it directly alters the expression of a plethora of virulence factors (Fig. 3 and 4). Moreover, we show that USA300 responds to pyruvate by upregulating the expression of leucocidins, which increases the pathogen’s virulence (Fig. 7).

The role of pyruvate in enhancing the pathogenicity of *S. aureus* could be important, as pyruvate is a central metabolite and prominent nutrient in the human host. The reported concentration of pyruvate ranges from 40 μM in individual cells to 10 mM in the blood of people with diabetes (42–45). Interestingly, diabetic patients have a higher propensity for pneumonia and foot infections caused by *S. aureus* (46–48). Additionally, it has been reported that diabetic mice showed a significantly higher bacterial burden after being challenged with *S. aureus* and had an impaired ability to clear the infection relative to nondiabetic mice (49). Together with the findings presented in our study, the association between increased pyruvate levels in diabetics and their increased susceptibility to *S. aureus* infection supports the notion that *S. aureus* sensing of pyruvate could be contributing to the observed enhanced pathogenicity.

On a more global level, our data further support the idea of a link between metabolism and virulence (7, 31). For instance, *S. aureus* senses changes in the levels of glucose and citrate through carbon catabolite repression regulators CcpA and CcpE, respectively, and this is linked to changes in central metabolism and modulation of virulence gene expression (50–52). By modeling the changes in flux that occur when *S. aureus* is exposed to pyruvate, we also saw significant alterations in the metabolic flux of pathways involved in central metabolism and amino acid metabolism, as well as in the transcript levels of enzymes that regulate these and other metabolic pathways (Fig. 4; see also Tables S2 and S3). Interestingly, however, while central metabolites are broadly involved in *S. aureus* virulence, among the tested metabolites, only pyruvate strongly induced the production of exoproteins (see Fig. S1 in the supplemental material), suggesting a specific impact of pyruvate on *S. aureus* virulence.

There are several caveats with respect to *in silico* metabolic flux analysis. The rationale behind E-Flux2 is that mRNA levels can be used as an approximate upper bound of the maximum concentration of metabolic enzymes and, consequently, as an upper limit on reaction rates (32). However, existing literature highlights the challenges associated with integrative transcriptomic and proteomic studies conducted to find strong correlations between mRNA and enzyme activity (53). Most notably, this approach does not directly consider regulatory processes that modulate the effective levels of enzyme activity in the system such as metabolite feedback regulation, allosteric interactions, and various posttranslational modifications that may alter the activity of the enzymes already synthesized (54). Nevertheless, because such modulations cannot lead to more enzyme activity under conditions in which all available enzymes are maximally active, the constraints defined by mRNA levels serve as a reliable maximal bound for enzyme amounts that cannot be exceeded. Still, future work utilizing traditional metabolomics to understand how intracellular metabolite levels are affected by the influx of pyruvate would provide more insight into changes in the metabolic flux of the system.

The relationship between pyruvate sensing and bacterial responses has been proposed in other bacterial species. In *Escherichia coli*, for example, pyruvate has been shown to be a low-affinity stimulus for the YpdAB TCS and a high-affinity stimulus for the BstSR TCS, with both serving to modulate the expression of putative transporters (55, 56). In *Bacillus subtilis*, crystallization of sensor histidine kinase KinD revealed that pyruvate is an endogenous ligand, presumably regulating sporulation; however, this has yet to be confirmed experimentally (57). Pairwise comparison of the amino acid sequences revealed 27% identity between *E. coli* sensors YpdB and BtsS and 15% to 16% identity between *B. subtilis* sensor KinD and either of the *E. coli* sensors. While no obvious motifs were revealed that might account for the shared pyruvate-sensing potential, comparison of ArlS with these revealed up to 18% sequence identity, which is within the range of sequence identity differences between the experimentally identified pyruvate sensors of different bacterial species. Together with our data, this supports a model in which ArlRS could be a pyruvate sensor in *S. aureus*. However, whether ArlS directly or indirectly senses pyruvate remains to be determined. Moreover, the molecular details of how ArlRS regulates *agr* also need to be fully elucidated.

Pathogens must adapt to their environment and survive changes in a given niche in response to infection. Thus, it is not surprising that, given the importance of pyruvate in host metabolism, *S. aureus* would have the ability to sense this metabolite and even usurp it accordingly for its own benefit. Understanding how pyruvate affects the pathogenesis of *S. aureus*, identifying the sensors responsible for monitoring pyruvate levels, and delineating the molecular mechanism of pyruvate’s regulatory role are crucial. Not only will this information define novel mechanisms in gene regulation, but it will also provide insight into the adaptive mechanisms that enable *S. aureus* to be such a versatile pathogen.

## MATERIALS AND METHODS

### Ethics statement.

Buffy coats were obtained from anonymous blood donors with informed consent from the New York Blood Center. Since all of the samples were collected anonymously prior to their delivery, the New York University Langone Health Institutional Review Board determined that our study was exempt from further ethics approval requirements.

All animal experiments were reviewed and approved by the Institutional Animal Care and Use Committee of New York University Langone Health. All experiments were performed according to NIH guidelines, the Animal Welfare Act, and U.S. federal law.

### Bacterial cultures and growth conditions.

*S. aureus* strains were streaked to single colonies on tryptic soy agar (TSA). Colonies were inoculated in yeast-Casamino Acids (YC) broth for overnight culture and then subsequently subcultured 1:100 to the post-exponential-growth phase (5 h, unless otherwise specified) in YC alone or YC supplemented with sodium pyruvate (Fisher) (YCP), as specified. Cultures were allowed to shake at 180 rpm and 37°C. TSA plates or YC broth was supplemented with antibiotics, as needed, to the following final concentrations: chloramphenicol to 10 μg/ml, kanamycin to 50 μg/ml, erythromycin to 5 μg/ml, tetracycline to 4 μg/ml, or spectomycin to 1,000 μg/ml.

### Construction of bacterial strains.

Cells of the USA300 LAC *arlR*::*bursa* strain were generated by phage transduction of the JE2 strain from the Nebraska Transposon Mutant Library (NTML) (40) using phage 80α into wild-type, erythromycin-sensitive LAC clone AH1263 (“A.H. LAC”) (58). The LAC strains with individual two-component deletions, including Δ*arlRS*, were generated by Jeff Boyd. Complementation of the Δ*arlRS* mutant strain was performed using the pJC1306 suicide plasmid (provided by John Chen). This strategy enabled the stable integration of *arlRS* with its endogenous promoter into the SaP1 site of mutant strains, resulting in a single-copy insertion of the *arlRS* locus into the chromosome (59).

### Bacterial reporters.

Wild-type Lac reporter strains driving the expression of either fluorescence (green fluorescent protein [GFP]) from the *agrP3* promoter (60) or the *sae* promoter (26) promoter or luminescence from the *lukED* promoter or the *lukSF-PVL* promoter were grown for ~16 h in YC media supplemented with 10 μg/ml of chloramphenicol in 96-well round-bottom plates (Corning). The overnight cultures were subcultured 1:100 into fresh YC media with or without 2% pyruvate in black flat-bottom plates and allowed to shake at 37°C with measurements of optical density at 600 nm (OD_600_) and either luminescence or fluorescence taken every 2 h. The luminescence reporters were measured directly from the plates, whereas the fluorescent reporters were washed and resuspended in phosphate-buffered saline (PBS) prior to measurements.

### Exoprotein isolation, Coomassie staining, and immunoblotting.

*S. aureus* strains were grown as described above, and cultures were normalized by optical density such that the cultures had the same number of cells. Exoproteins were collected by TCA precipitation from cultured supernatants, as previously described (28).

Protein samples were separated on SDS-PAGE gels and then either directly stained with Coomassie or transferred to a nitrocellulose membrane for detection of specific proteins. Membranes were blocked in 5% milk–human IgG, immunoblotted with polyclonal primary antibodies, and detected with a fluorescent Alexa Fluor-conjugated secondary antibody.

### Cytotoxicity assays.

Human PMNs were isolated from LeukoPaks of human blood samples using a dextran gradient as previously described (25, 26). For intoxication studies, bacterium-free culture supernatants (at a final intoxication level ranging from 0.3% to 20%) were collected, filter sterilized, frozen from 5-h subcultures of USA300 LAC cells, and grown in 5 ml YC or YCP for 5 h at 37°C. hPMNs were diluted to a final concentration of 2 × 10^5^ cells/well in a final volume of 80 μl/well in colorless RPMI medium supplemented with 0.1% human serum albumin (HSA) and 10 mM HEPES. PMNs were intoxicated for 1 h at 37°C with 20 μl of cultured supernatants. Ten microliters of CellTiter viability dye was added to the intoxication mixture, which was then incubated for 1.5 h at 37°C. The viability of the hPMNs was assessed using a PerkinElmer EnVision 2103 multilabel plate reader at 492 nm for CellTiter analysis.

### Infection of human PMNs.

To determine the effect of pyruvate on the ability of extracellular bacteria to kill primary human PMNs, 2 × 10^5^ cells were infected with wild-type or isogenic leucocidin-deficient strains of USA300 LAC bacteria, cultured as described above, at up to an MOI of 50 for 2 h at 37°C and 5% CO_2_. Cell death was determined as a measure of LDH release. In brief, equal volumes of supernatant and LDH reagent were incubated for 15 min at room temperature. LDH signal was measured using the EnVision 2103 plate reader.

### Murine intraperitoneal infection.

Five-week-old female ND4 Swiss-Webster mice (Envigo) were infected through the intraperitoneal route of infection with 300 μl of 1× PBS containing 0.5 × 10^8^ to 1 × 10^8^ CFU of wild-type USA300 strain LAC that had been subcultured for 5 h in YC media with or without 2% pyruvate. Infectious doses were normalized for cell density (OD_600_) and confirmed to be equal through the quantification of CFU. Infections were allowed to persist for ~42 h, at which time the animals were euthanized with CO_2_ and the indicated organs were harvested. To enumerate the number of bacteria existing in the peritoneum, 3 ml of 1× PBS was injected into the peritoneal cavity and the liquid exudate was measured for CFU. Infections were done on two separate occasions, with 5 mice/group per infection.

### Sample preparation and data analysis for quantitative mass spectrometry.

Three independent colonies from wild-type USA300 LAC were subcultured to the post-exponential phase (5 h), in the presence or absence of 2% pyruvate, and then normalized by optical density. Exoproteins were TCA precipitated from cultured supernatants as described above. For surface proteins, the pelleted bacteria were washed with PBS and treated with 3 M LiCl to fractionate the noncovalent cell wall proteins. In order to disrupt the outer cell membrane and fractionate the covalent cell wall proteins, the pellets were treated with 2 mg/ml lysostaphin–TSM (100 mM Tris [pH 7]; 500 mM sucrose; 10 mM MgCl_2_) buffer to minimize lysis. Equal volumes of the noncovalent and covalent fractions were pooled and TCA precipitated. The reconstituted protein isolates were prepared for liquid chromatography-mass spectrometry (LC-MS) analysis, digested, and eluted into an Orbitrap Fusion (Thermo Scientific) mass spectrometer as described in reference 28.

For data analysis, the MaxQuant software suite (version 1.5.2.8) was used for peptide and protein identifications and label-free quantitation using the iBAQ (Intensity-Based Absolute Quantification) method (61). The exoproteome and surface proteome raw files for each sample were combined as fractions and searched against a UniProt USA300 protein database downloaded on 18 September 2015 containing 2,607 entries. The peptide tolerance was set to 20 ppm for the first search, and the peptide tolerance was 4.5 ppm for the main search. Trypsin-specific cleavage was selected with 2 missed cleavages. A peptide spectral match (PSM) false-detection rate (FDR [*q* value]) of 1% and a protein FDR of 1% were selected for identification. iBAQ quantitation was performed using a minimum ratio of 2 and allowing unique peptides only. Matching between runs was allowed with a 0.7-min match time window and a 20-min alignment time window. Carbamidomethylation of Cys was added as a static modification. Oxidation of Met, deamidation of Asn and Gln, and acetylation of the protein N terminus were the variable modifications allowed.

Results were filtered to include proteins identified with 2 or more unique peptides in all three replicates under one experimental condition. iBAQ quantitation intensity values were log_2_ transformed, and missing values were imputed from the normal data distribution. A two-sided Welch’s *t* test was performed, correcting for multiple testing with the Benjamini-Hochberg procedure to control for FDR at 5%. Proteins with a *q* value of <0.05 were considered statistically significant.

### RNA isolation, RNA-Seq, and data analysis.

Total RNA was extracted from *S. aureus* cultures grown for 5 h in the presence or absence of 2% pyruvate, using an RNeasy extraction kit and following the manufacturer’s instructions and the method previously described by Carroll et al. (30). Transcriptome sequencing (RNA-Seq) libraries were prepared using an Illumina TruSeq Stranded Total RNA Library Prep kit, after ribodepletion was performed with an Epicenter Ribo-Zero Gold kit (catalog no. RZE1224), starting from 2 μg of DNase I-treated total RNA, following the manufacturer’s protocol, with the exception that 13 cycles of PCR were performed to amplify the libraries, to keep the duplication rate lower than that which occurs with the recommended 15 cycles. The amplified libraries were purified using AMPure beads, quantified by Qubit and qPCR, and visualized in an Agilent Bioanalyzer. The libraries were pooled equimolarly and loaded on an Illumina MiSeq flow cell (9v2) and run as paired 150-nucleotide reads.

FASTQC was performed on raw sequencing files to ensure the availability of reads of the proper quality before beginning data processing. The reads were then aligned against the USA300_FPR3757 ensemble reference genome/transcriptome utilizing the STAR/2.5 aligner. Subsequent gene counting of each sample was performed utilizing featureCounts/1.5.3. Normalization of sequence counts and differential gene expression analysis were performed using DESeq2/3.5, an R Bioconductor package (62). Genes with an adjusted *P* value of less than 0.05 were considered to be significantly differentiated. For heat maps, a variance-stabilizing transformation was applied to the normalized counts (62).

### qRT-PCR preparation and analysis.

For RT-qPCR, up to 100 ng of total RNA (prepared as described above) was used to perform QuantiTect one-step quantitative RT-PCR (qRT-PCR) with reverse transcriptase Mastermix and SYBR green master mix in an Applied Biosystems 7300 real-time PCR system. All genes were normalized to the housekeeping gene (the 16S rRNA gene). Fold change for target genes from LAC grown in the presence of pyruvate relative to the corresponding genes in LAC grown without pyruvate was determined using the threshold cycle (2^−ΔΔCT^) method of analysis.

### Computational metabolic flux prediction.

The E-Flux2 computational model was used to analyze the difference in intracellular metabolic fluxes between wild-type USA300 grown with (YCP) and wild-type USA300 grown without (YC) 2% pyruvate, as previously described (26), with the noted changes. Because we saw no significant difference in the levels of growth of YC-grown and YCP-grown *S. aureus*, the biomass fluxes of both strains were adjusted to 1 (in arbitrary units) and the other fluxes were normalized relatively. To account for the basal carbon sources in the complex media, influx reactions for all the amino acids were included in the model to best resemble the overlap in the compositions of YC and YCP media. In addition, we added a pyruvate influx reaction to our model for YCP simulations and removed flux corresponding to lactate dehydrogenase, an enzyme that catalyzes conversion pyruvate to lactate, as a simplifying assumption in our analysis. Lactate dehydrogenase is active primarily under anaerobic conditions; thus, under our experimental aerobic conditions, we would not expect flux toward this enzyme to be significant. Furthermore, because *S. aureus* does not synthesize polyamines (63), we removed reactions with the following enzymes from the model: agmatinase, arginine decarboxylase, ornithine decarboxylase, and spermidine synthase. Finally, because *S. aureus* encodes only an irreversible malate:quinone oxidoreductase, redundant malate dehydrogenase reactions were removed from the model. Significant fluxes used to calculate absolute fold changes were determined by considering both a computational rounding-off error set at 10^−9^ (e.g., 0.000000001 → 0) and the uncertainty of each flux value on the order of the average magnitude of all predicted fluxes in the network.

### Statistical analyses.

Prism software (GraphPad, Inc.) was used to perform statistical analyses. A Student’s *t* test was used for simple analyses of comparisons of two samples, whereas one-way or two-way analysis of variance (ANOVA) was used for multiple comparisons. The results were corrected for multiple comparisons by using the Bonferroni-corrected threshold. Statistical significance was considered to be represented by *P* values of <0.05.
