# Supplementary material for: Staphylococcus aureus Responds to the Central Metabolite Pyruvate To Regulate Virulence
Source: mBio. 2018 Jan 23;9(1):e02272-17. doi: 10.1128/mBio.02272-17 (PMC5784258; doi:10.1128/mBio.02272-17)
Supplement: FIG S1 [file mbo001183696sf1.pdf]

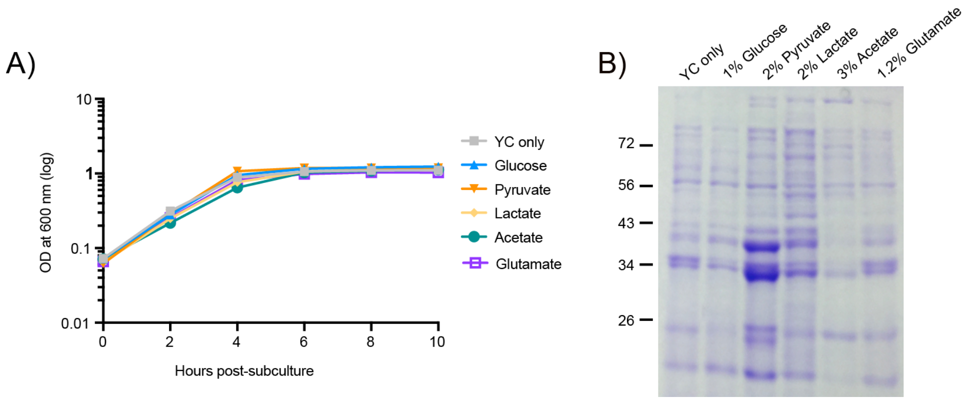

Supplemental Figure 1: Pyruvate has most pronounced effect of key central metabolites on the induction of the exoproteome. USA300 was cultured in YC media supplemented with either glucose, pyruvate, lactate, acetate, or glutamate, normalized to equal molar concentrations of carbon corresponding to 2% pyruvate and used to assess: the growth curve (A) and exoprotein profile at post-exponential growth phase (B).
