# Supplementary material for: Staphylococcus aureus Responds to the Central Metabolite Pyruvate To Regulate Virulence
Source: mBio. 2018 Jan 23;9(1):e02272-17. doi: 10.1128/mBio.02272-17 (PMC5784258; doi:10.1128/mBio.02272-17)
Supplement: TABLE S1 [file mbo001183696st1.docx]

**Supplemental Table 1:** Proteins that significantly changed in abundance in the presence of pyruvate (YCP).

| **Associated gene Name** | **Log_2_ iBAQ YCP1** | **Log_2_ iBAQ YCP 2** | **Log_2_ iBAQ YCP 3** | **Log_2_ iBAQ YC 1** | **Log_2_ iBAQ YC 2** | **Log_2_ iBAQ YC 3** | **Average Log_2_ Difference (Relative to YCP)** | **Stdev Log_2_ Difference** |
| --- | --- | --- | --- | --- | --- | --- | --- | --- |
| *xseA* | 18.88 | 18.32 | 18.09 | 11.04 | 12.92 | 13.64 | 5.90 | 1.74 |
| *carB* | 22.70 | 21.88 | 21.67 | 15.84 | 16.05 | 17.39 | 5.66 | 1.30 |
| *SAUSA300_0574* | 18.42 | 17.84 | 18.21 | 12.86 | 11.71 | 13.11 | 5.59 | 0.51 |
| *lukS-PVL* | 26.80 | 26.12 | 26.67 | 21.43 | 20.72 | 21.38 | 5.36 | 0.06 |
| *SAUSA300_1090* | 22.53 | 22.95 | 22.77 | 17.54 | 15.81 | 19.09 | 5.27 | 1.75 |
| *panE* | 18.35 | 17.17 | 18.03 | 10.73 | 14.35 | 12.89 | 5.19 | 2.40 |
| *splB* | 23.83 | 22.88 | 23.44 | 18.43 | 17.68 | 18.65 | 5.13 | 0.31 |
| *SAUSA300_1579* | 20.27 | 19.81 | 19.23 | 13.54 | 13.77 | 16.65 | 5.12 | 2.22 |
| *lukF-PVL* | 27.63 | 26.63 | 27.24 | 22.34 | 21.61 | 22.56 | 5.00 | 0.30 |
| *nrdD* | 21.84 | 21.36 | 21.01 | 15.23 | 16.29 | 17.78 | 4.97 | 1.69 |
| *pyrC* | 23.28 | 22.52 | 22.46 | 17.21 | 17.88 | 18.27 | 4.96 | 0.98 |
| *pyrE* | 20.41 | 19.25 | 18.59 | 12.71 | 16.17 | 14.52 | 4.95 | 2.44 |
| *fmt* | 17.78 | 17.72 | 17.99 | 13.21 | 11.85 | 13.92 | 4.84 | 0.93 |
| *splE* | 24.38 | 23.57 | 24.06 | 19.38 | 18.72 | 19.43 | 4.83 | 0.19 |
| *lukE* | 22.35 | 21.32 | 22.01 | 17.42 | 16.78 | 17.33 | 4.72 | 0.20 |
| *SAUSA300_1701* | 18.15 | 18.07 | 17.04 | 14.35 | 11.58 | 13.46 | 4.63 | 1.62 |
| *copB* | 17.14 | 17.11 | 16.95 | 10.90 | 12.85 | 13.64 | 4.60 | 1.50 |
| *SAUSA300_0231* | 20.58 | 20.08 | 20.52 | 16.22 | 15.28 | 16.01 | 4.55 | 0.22 |
| *SAUSA300_0079* | 22.37 | 21.86 | 22.61 | 18.17 | 17.62 | 17.43 | 4.54 | 0.56 |
| *SAUSA300_2314* | 17.09 | 16.90 | 16.51 | 11.19 | 12.15 | 13.59 | 4.52 | 1.50 |
| *SAUSA300_0553* | 23.29 | 22.40 | 23.12 | 18.73 | 18.10 | 18.55 | 4.48 | 0.15 |
| *nadK* | 17.89 | 17.56 | 17.42 | 13.60 | 12.24 | 13.63 | 4.46 | 0.78 |
| *carA* | 21.69 | 20.91 | 20.86 | 15.65 | 17.04 | 17.37 | 4.46 | 1.38 |
| *SAUSA300_0657* | 18.00 | 17.92 | 18.03 | 12.83 | 13.74 | 14.19 | 4.39 | 0.70 |
| *tagH* | 18.09 | 18.01 | 18.00 | 12.74 | 14.98 | 13.20 | 4.39 | 1.21 |
| *ipdC* | 22.80 | 22.87 | 22.52 | 17.80 | 18.06 | 19.20 | 4.38 | 0.92 |
| *arcB* | 18.15 | 17.84 | 17.15 | 14.58 | 12.60 | 13.00 | 4.32 | 0.85 |
| *SAUSA300_0590* | 16.31 | 17.96 | 17.68 | 12.24 | 12.93 | 13.84 | 4.31 | 0.63 |
| *radA* | 16.71 | 16.83 | 16.78 | 12.32 | 12.17 | 12.94 | 4.29 | 0.42 |
| *SAUSA300_0540* | 17.04 | 17.35 | 16.98 | 11.65 | 12.94 | 13.97 | 4.27 | 1.20 |
| *SAUSA300_1518* | 22.04 | 21.20 | 20.97 | 15.71 | 17.18 | 18.53 | 4.27 | 1.95 |
| *splC* | 22.37 | 21.16 | 22.25 | 18.02 | 17.63 | 17.35 | 4.26 | 0.69 |
| *alsS* | 22.90 | 22.90 | 22.32 | 17.85 | 18.01 | 19.50 | 4.25 | 1.24 |
| *topA* | 19.34 | 18.89 | 19.06 | 13.89 | 14.56 | 16.14 | 4.23 | 1.27 |
| *trmB* | 16.06 | 18.88 | 18.14 | 12.76 | 13.40 | 14.35 | 4.19 | 1.14 |
| *SAUSA300_2264* | 20.81 | 20.23 | 20.63 | 16.93 | 15.26 | 16.94 | 4.18 | 0.69 |
| *sraP* | 17.25 | 16.90 | 16.81 | 13.01 | 12.52 | 12.89 | 4.18 | 0.24 |
| *obg* | 23.49 | 23.41 | 22.64 | 18.97 | 18.20 | 20.18 | 4.06 | 1.43 |
| *SAUSA300_2378* | 15.72 | 16.60 | 16.37 | 12.09 | 12.86 | 11.74 | 4.00 | 0.55 |
| *aldA2* | 19.52 | 19.62 | 20.19 | 15.02 | 15.62 | 16.76 | 3.97 | 0.54 |
| *era* | 20.10 | 19.48 | 19.00 | 14.70 | 15.39 | 16.62 | 3.96 | 1.52 |
| *splD* | 24.45 | 23.82 | 24.30 | 20.47 | 19.79 | 20.46 | 3.95 | 0.10 |
| *SAUSA300_0194* | 16.84 | 16.63 | 16.02 | 12.35 | 12.35 | 12.97 | 3.94 | 0.78 |
| *SAUSA300_2473* | 19.72 | 19.20 | 18.46 | 14.12 | 14.96 | 16.48 | 3.94 | 1.83 |
| *hemL2* | 22.45 | 22.17 | 22.01 | 17.84 | 17.78 | 19.26 | 3.91 | 1.02 |
| *hutG* | 15.76 | 16.74 | 15.96 | 11.41 | 13.05 | 12.33 | 3.89 | 0.40 |
| *pyrF* | 18.43 | 17.42 | 17.27 | 13.26 | 12.75 | 15.47 | 3.88 | 1.82 |
| *arlR* | 18.70 | 18.19 | 18.18 | 13.57 | 14.30 | 15.59 | 3.87 | 1.27 |
| *lukD* | 22.94 | 22.14 | 22.71 | 18.87 | 18.73 | 18.58 | 3.87 | 0.40 |
| *SAUSA300_2357* | 18.36 | 17.50 | 17.56 | 14.97 | 12.49 | 14.54 | 3.81 | 1.05 |
| *splF* | 23.90 | 22.94 | 21.78 | 19.26 | 18.77 | 19.33 | 3.76 | 1.15 |
| *femX* | 20.75 | 20.50 | 20.15 | 15.72 | 16.28 | 18.19 | 3.73 | 1.59 |
| *vga* | 18.48 | 18.61 | 17.66 | 13.65 | 13.81 | 16.11 | 3.73 | 1.89 |
| *pcrA* | 19.62 | 19.02 | 18.59 | 14.04 | 15.46 | 16.56 | 3.72 | 1.78 |
| *SAUSA300_1873* | 18.34 | 18.32 | 17.74 | 13.55 | 14.46 | 15.26 | 3.71 | 1.16 |
| *prfC* | 21.69 | 21.85 | 21.04 | 16.80 | 17.48 | 19.27 | 3.68 | 1.67 |
| *SAUSA300_0905* | 17.66 | 18.41 | 18.54 | 14.54 | 13.52 | 15.54 | 3.67 | 1.06 |
| *cshA* | 22.16 | 21.91 | 21.28 | 17.28 | 17.60 | 19.46 | 3.67 | 1.63 |
| *SAUSA300_1716* | 16.81 | 16.50 | 16.27 | 12.64 | 12.31 | 13.63 | 3.67 | 0.89 |
| *acnA* | 24.31 | 23.75 | 23.36 | 19.32 | 20.17 | 20.94 | 3.66 | 1.29 |
| *yidC* | 17.00 | 17.14 | 18.15 | 12.99 | 14.23 | 14.16 | 3.64 | 0.63 |
| *sodM* | 23.55 | 23.34 | 23.12 | 19.41 | 19.51 | 20.27 | 3.61 | 0.68 |
| *SAUSA300_1494* | 20.35 | 20.63 | 19.81 | 15.77 | 16.45 | 17.76 | 3.60 | 1.36 |
| *SAUSA300_0655* | 23.48 | 22.77 | 22.72 | 19.07 | 19.25 | 19.86 | 3.60 | 0.78 |
| *SAUSA300_0328* | 17.71 | 18.02 | 16.83 | 13.77 | 13.79 | 14.22 | 3.59 | 0.86 |
| *SAUSA300_2631* | 17.89 | 17.60 | 17.80 | 13.50 | 14.73 | 14.40 | 3.55 | 0.77 |
| *sdaAB* | 20.05 | 19.24 | 19.23 | 15.57 | 15.03 | 17.27 | 3.55 | 1.38 |
| *SAUSA300_1795* | 19.95 | 19.60 | 19.43 | 15.32 | 15.72 | 17.34 | 3.53 | 1.30 |
| *SAUSA300_1978* | 17.13 | 17.48 | 17.54 | 14.05 | 13.51 | 13.99 | 3.53 | 0.45 |
| *rho* | 18.93 | 19.00 | 19.28 | 14.93 | 14.94 | 16.75 | 3.53 | 0.87 |
| *drp35* | 22.31 | 21.84 | 21.45 | 17.82 | 18.19 | 19.03 | 3.52 | 1.04 |
| *ffh* | 20.73 | 20.40 | 20.28 | 16.20 | 16.69 | 17.99 | 3.51 | 1.13 |
| *SAUSA300_0181* | 14.27 | 14.67 | 13.31 | 10.09 | 9.73 | 11.89 | 3.51 | 1.85 |
| *fmtB* | 17.08 | 16.72 | 16.20 | 13.72 | 11.83 | 13.92 | 3.51 | 1.31 |
| *rnz* | 18.37 | 18.20 | 17.65 | 14.10 | 15.20 | 14.62 | 3.43 | 0.72 |
| *SAUSA300_0945* | 18.26 | 18.83 | 18.19 | 14.18 | 14.46 | 16.34 | 3.43 | 1.38 |
| *dltD* | 18.46 | 17.33 | 18.03 | 13.76 | 14.76 | 15.02 | 3.43 | 1.12 |
| *SAUSA300_1654* | 22.80 | 22.62 | 22.40 | 18.22 | 19.28 | 20.07 | 3.42 | 1.13 |
| *SAUSA300_1674* | 21.33 | 21.10 | 21.30 | 17.32 | 17.77 | 18.40 | 3.41 | 0.56 |
| *srtA* | 19.97 | 18.99 | 19.50 | 15.40 | 15.80 | 17.10 | 3.39 | 1.10 |
| *SAUSA300_1658* | 18.44 | 18.36 | 18.56 | 14.65 | 14.98 | 15.58 | 3.39 | 0.41 |
| *estA* | 18.69 | 18.62 | 18.07 | 14.71 | 14.67 | 15.86 | 3.39 | 1.01 |
| *rsgA* | 16.29 | 16.62 | 14.75 | 12.03 | 11.89 | 13.60 | 3.38 | 1.94 |
| *SAUSA300_0252* | 18.51 | 18.36 | 18.65 | 14.94 | 14.60 | 15.83 | 3.38 | 0.49 |
| *treC* | 23.61 | 23.54 | 22.86 | 19.16 | 19.63 | 21.12 | 3.37 | 1.43 |
| *SAUSA300_1590* | 17.90 | 17.79 | 18.02 | 13.72 | 14.14 | 15.82 | 3.35 | 1.03 |
| *pyrR* | 19.06 | 18.54 | 18.56 | 15.25 | 14.95 | 15.93 | 3.34 | 0.63 |
| *mqo* | 18.77 | 18.74 | 18.42 | 14.51 | 14.78 | 16.60 | 3.34 | 1.33 |
| *hlgC* | 21.66 | 20.86 | 21.37 | 17.97 | 17.73 | 18.18 | 3.34 | 0.30 |
| *pyrG* | 24.19 | 24.21 | 23.68 | 20.14 | 20.43 | 21.53 | 3.33 | 1.03 |
| *SAUSA300_0589* | 20.40 | 20.13 | 19.97 | 16.28 | 16.45 | 17.81 | 3.32 | 1.02 |
| *pnbA* | 21.20 | 20.84 | 20.32 | 16.89 | 17.71 | 17.81 | 3.31 | 0.92 |
| *SAUSA300_0177* | 19.91 | 19.30 | 18.86 | 15.22 | 15.69 | 17.22 | 3.31 | 1.55 |
| *ftsK* | 15.36 | 14.65 | 14.05 | 10.94 | 10.89 | 12.29 | 3.31 | 1.38 |
| *pckA* | 22.56 | 22.32 | 22.06 | 18.37 | 18.84 | 19.80 | 3.31 | 0.98 |
| *dnaA* | 18.94 | 18.71 | 18.76 | 15.19 | 14.77 | 16.54 | 3.30 | 0.95 |
| *femA* | 21.66 | 21.36 | 21.24 | 17.63 | 17.43 | 19.37 | 3.27 | 1.22 |
| *icd* | 20.68 | 20.35 | 20.23 | 16.24 | 16.98 | 18.23 | 3.27 | 1.22 |
| *tsaD* | 19.38 | 18.59 | 18.66 | 14.94 | 15.10 | 16.79 | 3.27 | 1.30 |
| *trmFO* | 20.33 | 20.54 | 20.30 | 16.67 | 17.01 | 17.70 | 3.26 | 0.58 |
| *copZ* | 22.22 | 21.07 | 22.03 | 18.01 | 18.21 | 19.36 | 3.25 | 0.84 |
| *SAUSA300_0859* | 21.05 | 21.02 | 20.91 | 17.45 | 17.44 | 18.37 | 3.24 | 0.60 |
| *SAUSA300_2317* | 22.62 | 22.45 | 21.88 | 18.29 | 18.74 | 20.22 | 3.24 | 1.40 |
| *saeR* | 20.89 | 20.65 | 20.48 | 17.06 | 17.14 | 18.12 | 3.23 | 0.77 |
| *hemG* | 20.65 | 20.38 | 20.10 | 16.49 | 16.66 | 18.36 | 3.21 | 1.29 |
| *gatA* | 24.58 | 24.59 | 24.19 | 20.55 | 21.07 | 22.11 | 3.21 | 1.01 |
| *arcA* | 16.27 | 16.34 | 15.74 | 12.24 | 12.52 | 13.96 | 3.21 | 1.24 |
| *SAUSA300_2259* | 20.57 | 20.10 | 20.15 | 17.13 | 17.09 | 17.01 | 3.20 | 0.22 |
| *SAUSA300_1650* | 19.15 | 18.16 | 19.13 | 15.36 | 16.22 | 15.28 | 3.19 | 1.09 |
| *rsmH* | 20.89 | 20.37 | 20.83 | 16.64 | 17.54 | 18.35 | 3.19 | 0.94 |
| *SAUSA300_1450* | 18.79 | 18.84 | 18.69 | 15.63 | 14.62 | 16.55 | 3.17 | 1.04 |
| *SAUSA300_1750* | 16.07 | 14.67 | 15.95 | 13.20 | 11.82 | 12.20 | 3.15 | 0.52 |
| *SAUSA300_0841* | 16.75 | 16.59 | 16.35 | 12.85 | 13.07 | 14.30 | 3.15 | 0.98 |
| *SAUSA300_2146* | 19.78 | 19.15 | 19.32 | 15.77 | 15.77 | 17.32 | 3.14 | 1.03 |
| *glmS* | 24.14 | 24.25 | 23.97 | 20.44 | 20.65 | 21.90 | 3.12 | 0.92 |
| *SAUSA300_0251* | 16.25 | 15.47 | 16.51 | 11.85 | 13.47 | 13.56 | 3.12 | 1.20 |
| *SAUSA300_2542* | 22.35 | 22.24 | 21.85 | 18.25 | 18.82 | 20.04 | 3.11 | 1.18 |
| *SAUSA300_2076* | 23.14 | 23.16 | 22.85 | 19.30 | 19.83 | 20.73 | 3.09 | 0.88 |
| *SAUSA300_2354* | 17.93 | 17.76 | 17.63 | 14.49 | 13.96 | 15.59 | 3.09 | 0.93 |
| *proS* | 22.16 | 22.48 | 21.74 | 18.16 | 18.78 | 20.25 | 3.06 | 1.37 |
| *SAUSA300_2554* | 18.58 | 18.74 | 18.19 | 15.14 | 14.81 | 16.38 | 3.06 | 1.11 |
| *hemE* | 20.80 | 20.68 | 20.63 | 17.26 | 17.40 | 18.27 | 3.06 | 0.62 |
| *tig* | 24.61 | 24.78 | 24.12 | 20.80 | 21.40 | 22.16 | 3.05 | 0.97 |
| *SAUSA300_0695* | 16.02 | 17.98 | 17.21 | 13.53 | 13.50 | 15.06 | 3.04 | 1.26 |
| *splA* | 24.32 | 23.37 | 23.86 | 20.40 | 21.07 | 20.97 | 3.04 | 0.82 |
| *hemH* | 22.52 | 22.12 | 22.25 | 18.60 | 19.14 | 20.05 | 3.03 | 0.86 |
| *sspB* | 23.08 | 22.38 | 22.73 | 19.93 | 19.39 | 19.77 | 3.03 | 0.10 |
| *rpsD* | 25.32 | 24.82 | 24.21 | 21.18 | 21.63 | 22.46 | 3.03 | 1.20 |
| *pflA* | 21.46 | 21.23 | 20.88 | 18.17 | 18.00 | 18.33 | 3.02 | 0.41 |
| *murA* | 21.55 | 21.22 | 20.88 | 17.36 | 18.11 | 19.13 | 3.02 | 1.23 |
| *bshC* | 18.83 | 19.21 | 18.91 | 15.24 | 15.72 | 16.94 | 3.02 | 0.91 |
| *SAUSA300_1911* | 16.44 | 16.11 | 17.16 | 13.10 | 13.06 | 14.51 | 3.01 | 0.35 |
| *folC* | 17.56 | 17.55 | 17.68 | 14.32 | 14.07 | 15.38 | 3.00 | 0.63 |
| *mutS2* | 16.98 | 16.94 | 15.89 | 12.69 | 13.13 | 15.00 | 3.00 | 1.84 |
| *pepF* | 21.54 | 21.80 | 21.50 | 18.18 | 18.63 | 19.09 | 2.98 | 0.50 |
| *SAUSA300_2422* | 21.37 | 21.00 | 20.46 | 17.19 | 17.91 | 18.79 | 2.98 | 1.26 |
| *SAUSA300_0930* | 22.86 | 22.76 | 22.25 | 18.63 | 19.56 | 20.75 | 2.98 | 1.38 |
| *walR* | 22.86 | 22.39 | 22.08 | 18.86 | 19.35 | 20.19 | 2.97 | 1.06 |
| *cysE* | 15.23 | 16.03 | 15.67 | 12.55 | 12.43 | 13.04 | 2.97 | 0.55 |
| *ampA* | 21.75 | 21.32 | 21.13 | 17.92 | 18.40 | 18.97 | 2.97 | 0.83 |
| *SAUSA300_0086* | 15.81 | 14.88 | 15.31 | 11.69 | 12.49 | 12.93 | 2.97 | 1.00 |
| *SAUSA300_2236* | 20.02 | 19.22 | 19.88 | 15.86 | 16.78 | 17.59 | 2.96 | 1.04 |
| *SAUSA300_0883* | 25.10 | 24.90 | 25.18 | 22.39 | 21.95 | 21.97 | 2.96 | 0.25 |
| *aur* | 22.17 | 22.07 | 22.15 | 19.19 | 18.88 | 19.47 | 2.95 | 0.25 |
| *femB* | 20.63 | 20.64 | 21.14 | 17.24 | 18.14 | 18.22 | 2.94 | 0.44 |
| *moaE* | 17.09 | 17.15 | 16.74 | 13.32 | 13.71 | 15.13 | 2.94 | 1.17 |
| *rpsR* | 22.50 | 21.39 | 21.23 | 18.66 | 18.35 | 19.29 | 2.94 | 0.96 |
| *lysS* | 22.04 | 22.10 | 22.21 | 18.63 | 18.89 | 20.02 | 2.94 | 0.65 |
| *ubiE* | 18.02 | 18.09 | 19.06 | 14.46 | 15.47 | 16.45 | 2.93 | 0.54 |
| *SAUSA300_2085* | 19.67 | 19.40 | 19.28 | 16.59 | 15.73 | 17.26 | 2.93 | 0.84 |
| *SAUSA300_1304* | 24.48 | 23.96 | 23.81 | 20.49 | 21.09 | 21.95 | 2.91 | 1.06 |
| *murE* | 19.84 | 19.95 | 19.47 | 16.27 | 16.54 | 17.75 | 2.90 | 1.02 |
| *SAUSA300_2513* | 16.61 | 16.45 | 15.49 | 13.63 | 11.98 | 14.26 | 2.90 | 1.63 |
| *SAUSA300_0082* | 16.76 | 16.38 | 16.15 | 12.83 | 13.33 | 14.44 | 2.89 | 1.12 |
| *nagA* | 21.53 | 21.20 | 21.29 | 18.04 | 18.03 | 19.26 | 2.89 | 0.77 |
| *secA1* | 22.15 | 21.96 | 21.61 | 18.30 | 18.74 | 20.04 | 2.88 | 1.18 |
| *lyrA* | 17.40 | 17.19 | 17.24 | 13.66 | 14.68 | 14.86 | 2.88 | 0.75 |
| *SAUSA300_0981* | 19.52 | 19.72 | 18.87 | 15.47 | 16.61 | 17.42 | 2.87 | 1.32 |
| *araB* | 17.25 | 17.51 | 16.60 | 14.59 | 13.24 | 14.93 | 2.87 | 1.31 |
| *SAUSA300_1533* | 19.85 | 19.68 | 19.76 | 16.49 | 16.58 | 17.67 | 2.85 | 0.67 |
| *parE* | 19.21 | 19.23 | 19.00 | 15.47 | 16.11 | 17.33 | 2.84 | 1.06 |
| *msrA* | 22.14 | 21.39 | 21.38 | 18.12 | 18.78 | 19.48 | 2.84 | 1.08 |
| *SAUSA300_2147* | 20.41 | 20.07 | 19.82 | 16.71 | 17.06 | 18.00 | 2.84 | 0.95 |
| *SAUSA300_0550* | 17.19 | 17.16 | 17.02 | 14.29 | 13.49 | 15.07 | 2.84 | 0.86 |
| *mnmA* | 21.29 | 21.03 | 20.44 | 17.42 | 17.74 | 19.11 | 2.83 | 1.33 |
| *SAUSA300_1787* | 19.08 | 18.78 | 19.28 | 15.94 | 16.09 | 16.64 | 2.82 | 0.27 |
| *SAUSA300_0250* | 22.22 | 21.65 | 21.38 | 18.17 | 18.87 | 19.77 | 2.81 | 1.22 |
| *panC* | 19.09 | 19.36 | 19.01 | 15.67 | 16.16 | 17.18 | 2.81 | 0.86 |
| *coaW* | 18.03 | 17.91 | 17.27 | 14.47 | 14.41 | 15.89 | 2.81 | 1.24 |
| *gatB* | 23.37 | 23.29 | 23.14 | 19.90 | 20.10 | 21.35 | 2.81 | 0.91 |
| *purR* | 19.30 | 18.98 | 19.26 | 15.63 | 16.31 | 17.18 | 2.81 | 0.81 |
| *qoxA* | 22.50 | 22.08 | 22.19 | 19.22 | 19.27 | 19.87 | 2.81 | 0.47 |
| *argG* | 19.03 | 19.05 | 18.79 | 15.86 | 16.20 | 16.43 | 2.79 | 0.41 |
| *SAUSA300_1170* | 15.94 | 16.81 | 16.60 | 13.25 | 13.86 | 13.86 | 2.79 | 0.14 |
| *SAUSA300_0383* | 21.00 | 20.55 | 20.78 | 17.85 | 17.89 | 18.20 | 2.79 | 0.31 |
| *scrB* | 21.23 | 21.07 | 20.74 | 17.57 | 18.00 | 19.15 | 2.78 | 1.07 |
| *SAUSA300_0576* | 20.10 | 19.86 | 19.81 | 16.49 | 17.23 | 17.75 | 2.76 | 0.78 |
| *SAUSA300_0569* | 22.54 | 22.16 | 22.25 | 18.88 | 19.75 | 20.02 | 2.76 | 0.78 |
| *metK* | 23.35 | 22.99 | 22.68 | 19.73 | 19.86 | 21.17 | 2.75 | 1.11 |
| *SAUSA300_1463* | 19.75 | 19.31 | 19.55 | 16.62 | 16.27 | 17.45 | 2.75 | 0.57 |
| *ychF* | 22.62 | 22.38 | 22.44 | 19.10 | 19.38 | 20.73 | 2.74 | 0.93 |
| *rpsU* | 24.27 | 23.57 | 23.85 | 21.08 | 20.61 | 21.77 | 2.74 | 0.58 |
| *SAUSA300_0995* | 25.33 | 25.12 | 24.75 | 21.78 | 22.12 | 23.08 | 2.74 | 0.96 |
| *hemL1* | 23.38 | 23.17 | 23.26 | 20.02 | 20.15 | 21.42 | 2.74 | 0.80 |
| *pheT* | 23.45 | 23.31 | 23.15 | 20.14 | 20.52 | 21.04 | 2.74 | 0.60 |
| *pflB* | 27.47 | 27.50 | 26.91 | 23.78 | 24.26 | 25.63 | 2.74 | 1.28 |
| *tyrS* | 22.13 | 22.04 | 22.01 | 18.59 | 19.28 | 20.10 | 2.74 | 0.82 |
| *SAUSA300_1687* | 19.60 | 19.10 | 19.15 | 15.88 | 16.39 | 17.39 | 2.74 | 0.98 |
| *rnc* | 17.57 | 18.03 | 18.00 | 14.83 | 15.91 | 14.66 | 2.73 | 0.61 |
| *SAUSA300_1369* | 22.92 | 22.60 | 22.10 | 19.37 | 19.55 | 20.51 | 2.73 | 1.02 |
| *glmU* | 21.65 | 21.38 | 21.01 | 17.94 | 18.31 | 19.60 | 2.73 | 1.19 |
| *SAUSA300_1691* | 20.45 | 20.61 | 20.48 | 17.11 | 17.69 | 18.55 | 2.73 | 0.73 |
| *cdr* | 22.96 | 22.79 | 22.43 | 19.48 | 19.95 | 20.59 | 2.73 | 0.83 |
| *rnr* | 19.58 | 19.08 | 18.98 | 15.79 | 16.30 | 17.39 | 2.72 | 1.10 |
| *SAUSA300_2432* | 18.52 | 18.12 | 18.45 | 15.79 | 14.73 | 16.42 | 2.72 | 0.68 |
| *SAUSA300_0925* | 16.77 | 17.17 | 16.35 | 13.18 | 14.66 | 14.30 | 2.72 | 0.79 |
| *mqo* | 23.78 | 23.67 | 23.88 | 20.48 | 20.89 | 21.80 | 2.72 | 0.61 |
| *SAUSA300_1685* | 17.95 | 17.70 | 18.21 | 14.54 | 14.91 | 16.26 | 2.72 | 0.73 |
| *sdhA* | 20.64 | 20.58 | 20.20 | 16.96 | 17.61 | 18.75 | 2.70 | 1.14 |
| *SAUSA300_1536* | 21.08 | 21.30 | 20.78 | 17.78 | 17.95 | 19.34 | 2.70 | 1.09 |
| *rpsA* | 24.27 | 23.98 | 23.94 | 20.95 | 21.18 | 21.98 | 2.69 | 0.68 |
| *SAUSA300_1323* | 21.35 | 20.94 | 20.52 | 18.00 | 18.71 | 18.03 | 2.69 | 0.59 |
| *SAUSA300_0355* | 22.87 | 22.84 | 22.78 | 19.71 | 19.98 | 20.72 | 2.69 | 0.57 |
| *dut* | 23.32 | 23.00 | 22.34 | 19.30 | 20.46 | 20.83 | 2.69 | 1.26 |
| *SAUSA300_0557* | 19.58 | 19.34 | 18.75 | 15.75 | 16.76 | 17.10 | 2.68 | 1.09 |
| *fabF* | 24.61 | 24.30 | 24.10 | 21.21 | 21.39 | 22.38 | 2.68 | 0.86 |
| *pbp2* | 22.59 | 22.39 | 22.69 | 19.84 | 19.46 | 20.34 | 2.68 | 0.30 |
| *SAUSA300_2483* | 20.95 | 20.63 | 20.53 | 17.43 | 17.75 | 18.89 | 2.67 | 0.96 |
| *SAUSA300_1683* | 20.74 | 20.66 | 20.56 | 17.52 | 18.18 | 18.27 | 2.67 | 0.49 |
| *mnmG* | 20.90 | 20.84 | 20.81 | 17.64 | 18.08 | 18.84 | 2.66 | 0.65 |
| *qoxB* | 19.17 | 19.37 | 19.43 | 15.97 | 16.57 | 17.45 | 2.66 | 0.62 |
| *tuf* | 29.39 | 28.96 | 28.82 | 25.65 | 26.10 | 27.45 | 2.66 | 1.20 |
| *SAUSA300_2315* | 19.21 | 18.82 | 19.54 | 15.95 | 16.24 | 17.40 | 2.66 | 0.56 |
| *SAUSA300_1689* | 18.34 | 18.65 | 18.18 | 16.00 | 16.29 | 14.95 | 2.64 | 0.51 |
| *rpsC* | 24.37 | 23.98 | 23.91 | 20.59 | 21.41 | 22.35 | 2.64 | 1.12 |
| *rbsK* | 21.70 | 21.43 | 21.14 | 18.68 | 18.60 | 19.09 | 2.63 | 0.51 |
| *emp* | 20.67 | 20.99 | 21.20 | 18.49 | 17.78 | 18.71 | 2.62 | 0.52 |
| *SAUSA300_2094* | 20.85 | 20.73 | 20.53 | 17.50 | 17.76 | 19.00 | 2.62 | 0.96 |
| *SAUSA300_1349* | 17.61 | 17.29 | 16.66 | 14.11 | 14.27 | 15.33 | 2.61 | 1.14 |
| *pdhA* | 25.63 | 24.93 | 25.06 | 22.19 | 22.47 | 23.12 | 2.61 | 0.76 |
| *parC* | 19.29 | 19.25 | 19.56 | 15.99 | 16.66 | 17.63 | 2.61 | 0.69 |
| *SAUSA300_0733* | 20.20 | 19.84 | 19.74 | 16.79 | 17.40 | 17.77 | 2.61 | 0.74 |
| *odhB* | 18.95 | 18.52 | 17.87 | 14.86 | 15.84 | 16.80 | 2.61 | 1.51 |
| *hlgB* | 22.37 | 21.84 | 22.38 | 20.10 | 19.35 | 19.33 | 2.60 | 0.40 |
| *purB* | 21.79 | 21.90 | 21.85 | 19.04 | 18.95 | 19.76 | 2.60 | 0.45 |
| *cvfB* | 20.26 | 19.84 | 20.00 | 16.91 | 17.40 | 18.00 | 2.60 | 0.69 |
| *gyrB* | 19.44 | 19.54 | 19.38 | 16.23 | 16.61 | 17.73 | 2.60 | 0.83 |
| *mvk* | 19.73 | 19.39 | 19.18 | 16.33 | 17.16 | 17.03 | 2.60 | 0.70 |
| *sarR* | 21.27 | 20.73 | 21.29 | 18.51 | 17.61 | 19.41 | 2.59 | 0.64 |
| *fmt* | 19.68 | 19.45 | 19.42 | 16.33 | 16.75 | 17.70 | 2.59 | 0.82 |
| *manA* | 18.92 | 19.72 | 19.80 | 16.67 | 16.80 | 17.23 | 2.59 | 0.33 |
| *SAUSA300_0769* | 21.74 | 21.02 | 21.19 | 18.98 | 17.90 | 19.32 | 2.58 | 0.64 |
| *SAUSA300_0871* | 24.11 | 23.95 | 23.50 | 20.72 | 21.29 | 21.82 | 2.58 | 0.85 |
| *gpmA* | 25.12 | 24.55 | 24.71 | 21.78 | 21.85 | 23.00 | 2.58 | 0.82 |
| *SAUSA300_1890* | 24.46 | 24.41 | 24.30 | 22.02 | 21.52 | 21.90 | 2.58 | 0.27 |
| *tdk* | 19.75 | 19.90 | 19.28 | 16.48 | 16.61 | 18.11 | 2.58 | 1.22 |
| *ackA* | 24.60 | 24.35 | 24.06 | 21.16 | 21.57 | 22.55 | 2.58 | 0.98 |
| *SAUSA300_0672* | 20.98 | 20.62 | 21.28 | 18.11 | 18.48 | 18.58 | 2.57 | 0.38 |
| *SAUSA300_1491* | 23.69 | 23.45 | 23.32 | 20.43 | 20.58 | 21.75 | 2.57 | 0.88 |
| *leuS* | 22.92 | 22.75 | 22.58 | 19.67 | 20.02 | 20.88 | 2.56 | 0.79 |
| *rluB* | 21.49 | 20.67 | 21.28 | 17.94 | 19.01 | 18.82 | 2.56 | 0.95 |
| *SAUSA300_1460* | 20.36 | 19.94 | 20.17 | 17.62 | 17.04 | 18.16 | 2.55 | 0.47 |
| *SAUSA300_1172* | 20.18 | 20.06 | 19.66 | 17.11 | 16.98 | 18.16 | 2.55 | 0.91 |
| *SAUSA300_0214* | 21.41 | 21.09 | 21.07 | 18.34 | 18.29 | 19.30 | 2.54 | 0.68 |
| *SAUSA300_0173* | 19.75 | 20.04 | 19.46 | 16.99 | 17.07 | 17.56 | 2.54 | 0.57 |
| *coaE* | 20.20 | 19.88 | 19.95 | 17.31 | 17.10 | 17.98 | 2.54 | 0.50 |
| *ddl* | 22.23 | 21.95 | 21.63 | 18.75 | 19.01 | 20.42 | 2.54 | 1.18 |
| *SAUSA300_1520* | 20.08 | 19.38 | 19.60 | 17.81 | 16.45 | 17.18 | 2.54 | 0.35 |
| *SAUSA300_1197* | 19.70 | 19.93 | 19.89 | 17.29 | 17.10 | 17.50 | 2.54 | 0.24 |
| *SAUSA300_2102* | 20.64 | 20.65 | 20.47 | 17.69 | 17.86 | 18.59 | 2.54 | 0.58 |
| *SAUSA300_0912* | 23.31 | 23.00 | 23.01 | 19.89 | 20.73 | 21.08 | 2.54 | 0.78 |
| *rimM* | 21.12 | 20.60 | 20.41 | 17.39 | 18.25 | 18.87 | 2.54 | 1.10 |
| *SAUSA300_2296* | 18.16 | 17.56 | 17.77 | 15.40 | 14.83 | 15.66 | 2.53 | 0.37 |
| *fhs* | 23.48 | 23.26 | 23.00 | 20.23 | 20.78 | 21.14 | 2.53 | 0.70 |
| *fbp* | 21.38 | 21.44 | 20.97 | 18.07 | 18.75 | 19.37 | 2.53 | 0.87 |
| *SAUSA300_0716* | 22.19 | 22.29 | 21.89 | 18.69 | 19.61 | 20.47 | 2.53 | 1.05 |
| *gcvT* | 20.34 | 19.80 | 19.99 | 16.79 | 17.57 | 18.19 | 2.53 | 0.91 |
| *SAUSA300_2272* | 18.80 | 18.55 | 18.37 | 15.28 | 16.35 | 16.50 | 2.53 | 0.87 |
| *SAUSA300_0213* | 16.51 | 16.12 | 15.02 | 13.31 | 12.61 | 14.15 | 2.53 | 1.44 |
| *SAUSA300_2387* | 15.39 | 15.54 | 15.60 | 12.84 | 12.61 | 13.50 | 2.52 | 0.42 |
| *map* | 22.11 | 21.92 | 21.85 | 19.01 | 19.29 | 20.02 | 2.52 | 0.64 |
| *SAUSA300_1970* | 18.09 | 17.66 | 17.49 | 15.12 | 14.83 | 15.72 | 2.52 | 0.66 |
| *SAUSA300_2251* | 21.12 | 20.85 | 21.22 | 18.10 | 18.12 | 19.45 | 2.51 | 0.66 |
| *moeA* | 22.95 | 22.54 | 22.19 | 19.42 | 20.03 | 20.69 | 2.51 | 1.02 |
| *SAUSA300_1688* | 23.11 | 22.72 | 22.26 | 19.50 | 20.22 | 20.83 | 2.51 | 1.09 |
| *SAUSA300_1181* | 20.79 | 20.27 | 19.84 | 17.51 | 17.54 | 18.35 | 2.50 | 0.91 |
| *infA* | 21.26 | 20.22 | 21.10 | 19.12 | 18.65 | 17.32 | 2.50 | 1.15 |
| *SAUSA300_2255* | 14.23 | 15.75 | 16.01 | 11.83 | 13.22 | 13.47 | 2.49 | 0.08 |
| *prfA* | 22.12 | 21.58 | 21.73 | 18.83 | 18.97 | 20.17 | 2.49 | 0.87 |
| *tpx* | 24.09 | 23.85 | 23.94 | 20.92 | 21.64 | 21.87 | 2.48 | 0.60 |
| *gpsA* | 19.52 | 19.24 | 19.05 | 16.05 | 16.86 | 17.46 | 2.48 | 0.94 |
| *SAUSA300_1464* | 20.24 | 20.16 | 19.69 | 17.01 | 17.11 | 18.54 | 2.47 | 1.15 |
| *SAUSA300_1884* | 20.71 | 20.35 | 20.16 | 17.51 | 17.83 | 18.48 | 2.47 | 0.76 |
| *SAUSA300_0592* | 16.58 | 16.21 | 17.57 | 14.79 | 13.44 | 14.74 | 2.47 | 0.58 |
| *msrA* | 19.84 | 19.08 | 19.49 | 16.53 | 17.03 | 17.46 | 2.47 | 0.73 |
| *SAUSA300_0042* | 20.61 | 19.92 | 19.31 | 16.99 | 16.87 | 18.59 | 2.47 | 1.54 |
| *valS* | 23.41 | 23.42 | 23.10 | 20.35 | 20.68 | 21.52 | 2.46 | 0.77 |
| *SAUSA300_1969* | 20.25 | 19.70 | 20.05 | 17.04 | 17.73 | 17.84 | 2.46 | 0.65 |
| *miaB* | 20.44 | 20.29 | 20.32 | 17.43 | 17.61 | 18.64 | 2.45 | 0.69 |
| *rpoC* | 23.63 | 23.25 | 23.13 | 20.08 | 20.91 | 21.66 | 2.45 | 1.04 |
| *ftsZ* | 24.16 | 23.69 | 23.62 | 20.82 | 20.88 | 22.41 | 2.45 | 1.11 |
| *atpD* | 23.54 | 23.33 | 23.43 | 20.50 | 20.70 | 21.76 | 2.45 | 0.71 |
| *aspS* | 23.23 | 23.42 | 22.90 | 20.15 | 20.66 | 21.40 | 2.45 | 0.83 |
| *hemC* | 21.77 | 21.61 | 21.50 | 18.77 | 19.10 | 19.71 | 2.44 | 0.61 |
| *SAUSA300_1648* | 23.22 | 22.98 | 22.98 | 20.11 | 20.50 | 21.29 | 2.43 | 0.71 |
| *queC* | 18.95 | 19.59 | 18.65 | 15.74 | 16.64 | 17.53 | 2.43 | 1.14 |
| *pgcA* | 21.06 | 20.90 | 20.57 | 18.04 | 18.22 | 19.00 | 2.42 | 0.76 |
| *clpX* | 22.00 | 21.82 | 21.80 | 18.78 | 19.36 | 20.22 | 2.42 | 0.82 |
| *SAUSA300_0601* | 19.75 | 19.66 | 19.82 | 16.94 | 17.53 | 17.49 | 2.42 | 0.35 |
| *SAUSA300_0136* | 19.80 | 19.30 | 18.93 | 17.79 | 16.12 | 16.85 | 2.42 | 0.66 |
| *typA* | 21.84 | 21.95 | 21.74 | 18.83 | 19.11 | 20.33 | 2.42 | 0.88 |
| *prsA* | 24.45 | 23.92 | 24.21 | 21.88 | 21.34 | 22.11 | 2.42 | 0.28 |
| *alaS* | 21.99 | 21.95 | 21.47 | 18.72 | 19.09 | 20.35 | 2.42 | 1.15 |
| *SAUSA300_1909* | 20.31 | 20.27 | 20.26 | 17.39 | 18.27 | 17.93 | 2.42 | 0.47 |
| *treR* | 18.67 | 17.95 | 17.90 | 15.09 | 15.52 | 16.67 | 2.41 | 1.18 |
| *opuCa* | 18.09 | 17.62 | 18.29 | 15.39 | 15.04 | 16.36 | 2.40 | 0.42 |
| *SAUSA300_0598* | 19.80 | 19.34 | 19.54 | 17.19 | 16.83 | 17.46 | 2.40 | 0.28 |
| *pknB* | 19.15 | 18.59 | 19.22 | 16.52 | 16.40 | 16.87 | 2.39 | 0.22 |
| *SAUSA300_1007* | 19.24 | 19.23 | 18.73 | 16.45 | 17.28 | 16.30 | 2.39 | 0.42 |
| *SAUSA300_0844* | 22.46 | 22.38 | 22.26 | 19.23 | 20.15 | 20.56 | 2.39 | 0.78 |
| *SAUSA300_0696* | 18.46 | 17.79 | 17.36 | 15.23 | 16.10 | 15.13 | 2.38 | 0.78 |
| *rplY* | 26.49 | 25.59 | 25.72 | 23.10 | 23.64 | 23.91 | 2.38 | 0.87 |
| *SAUSA300_1082* | 19.70 | 19.81 | 19.04 | 17.05 | 16.87 | 17.50 | 2.38 | 0.74 |
| *plsX* | 20.76 | 20.99 | 20.71 | 17.91 | 18.46 | 18.96 | 2.38 | 0.56 |
| *glnA* | 23.77 | 23.75 | 23.44 | 20.89 | 21.22 | 21.72 | 2.38 | 0.60 |
| *SAUSA300_1585* | 19.15 | 19.31 | 19.04 | 16.34 | 16.55 | 17.49 | 2.37 | 0.71 |
| *ear* | 22.36 | 21.85 | 22.42 | 19.98 | 19.63 | 19.89 | 2.37 | 0.16 |
| *serS* | 24.36 | 24.16 | 23.87 | 21.32 | 21.58 | 22.37 | 2.37 | 0.79 |
| *SAUSA300_1572* | 19.24 | 19.27 | 20.62 | 17.13 | 16.63 | 18.24 | 2.37 | 0.27 |
| *ilvE* | 22.91 | 22.49 | 22.36 | 19.65 | 20.04 | 20.97 | 2.37 | 0.94 |
| *SAUSA300_1631* | 15.46 | 15.76 | 15.24 | 12.74 | 13.20 | 13.41 | 2.37 | 0.47 |
| *spsB* | 19.89 | 19.91 | 20.06 | 17.33 | 17.59 | 17.82 | 2.37 | 0.17 |
| *phoH* | 16.97 | 16.11 | 16.57 | 13.88 | 13.94 | 14.73 | 2.36 | 0.65 |
| *SAUSA300_2070* | 16.58 | 17.62 | 16.94 | 14.24 | 15.08 | 14.73 | 2.36 | 0.17 |
| *murI* | 18.06 | 17.99 | 17.07 | 14.83 | 14.90 | 16.32 | 2.36 | 1.39 |
| *SAUSA300_1119* | 22.56 | 22.82 | 22.26 | 19.37 | 20.17 | 21.03 | 2.36 | 1.01 |
| *prs* | 21.51 | 21.21 | 21.09 | 18.37 | 18.65 | 19.73 | 2.36 | 0.91 |
| *SAUSA300_0834* | 20.56 | 20.81 | 19.45 | 17.90 | 17.17 | 18.69 | 2.35 | 1.46 |
| *SAUSA300_1182* | 19.28 | 19.46 | 19.28 | 16.64 | 17.00 | 17.32 | 2.35 | 0.35 |
| *mvaD* | 19.30 | 18.35 | 19.12 | 16.58 | 15.92 | 17.22 | 2.35 | 0.42 |
| *SAUSA300_2267* | 16.19 | 16.00 | 17.01 | 14.21 | 13.16 | 14.80 | 2.34 | 0.44 |
| *fda* | 24.16 | 23.94 | 24.10 | 21.36 | 21.58 | 22.23 | 2.34 | 0.47 |
| *SAUSA300_2258* | 21.02 | 21.01 | 20.59 | 18.00 | 18.17 | 19.42 | 2.34 | 1.02 |
| *SAUSA300_0552* | 20.29 | 19.79 | 19.32 | 16.51 | 18.07 | 17.81 | 2.34 | 1.26 |
| *hslO* | 21.59 | 21.10 | 21.20 | 18.55 | 18.57 | 19.77 | 2.33 | 0.82 |
| *tkt* | 25.23 | 25.11 | 24.68 | 22.17 | 22.57 | 23.29 | 2.33 | 0.86 |
| *SAUSA300_2492* | 20.77 | 20.26 | 20.31 | 17.54 | 17.77 | 19.04 | 2.33 | 0.99 |
| *SAUSA300_0637* | 16.49 | 15.95 | 16.29 | 13.66 | 13.55 | 14.55 | 2.32 | 0.55 |
| *fabH* | 23.41 | 23.26 | 23.04 | 20.43 | 20.92 | 21.41 | 2.32 | 0.67 |
| *purA* | 21.32 | 20.92 | 20.94 | 18.04 | 18.69 | 19.49 | 2.32 | 0.92 |
| *rpsP* | 23.25 | 22.80 | 22.68 | 20.24 | 20.63 | 20.93 | 2.31 | 0.64 |
| *SAUSA300_2132* | 23.12 | 22.80 | 22.70 | 20.20 | 20.54 | 20.96 | 2.31 | 0.59 |
| *nusA* | 23.49 | 23.23 | 23.02 | 20.46 | 20.75 | 21.60 | 2.31 | 0.82 |
| *SAUSA300_1101* | 20.31 | 19.94 | 19.48 | 17.17 | 17.35 | 18.31 | 2.30 | 1.02 |
| *sarA* | 22.75 | 22.23 | 23.01 | 20.33 | 19.86 | 20.91 | 2.30 | 0.17 |
| *SAUSA300_0857* | 23.54 | 23.05 | 22.77 | 20.26 | 20.74 | 21.48 | 2.30 | 1.00 |
| *sirA* | 21.03 | 20.71 | 20.82 | 18.60 | 18.26 | 18.83 | 2.29 | 0.26 |
| *SAUSA300_1624* | 21.45 | 21.45 | 21.39 | 18.69 | 19.21 | 19.53 | 2.29 | 0.45 |
| *cysS* | 22.35 | 22.33 | 22.03 | 19.51 | 19.85 | 20.49 | 2.28 | 0.67 |
| *SAUSA300_2021* | 19.74 | 20.16 | 19.80 | 17.26 | 17.45 | 18.19 | 2.27 | 0.58 |
| *lip* | 23.55 | 23.38 | 23.66 | 21.56 | 20.95 | 21.30 | 2.27 | 0.24 |
| *SAUSA300_1690* | 21.46 | 21.22 | 21.09 | 18.63 | 19.00 | 19.35 | 2.26 | 0.55 |
| *odhA* | 19.21 | 19.14 | 18.81 | 16.17 | 16.56 | 17.66 | 2.26 | 0.99 |
| *SAUSA300_0688* | 19.13 | 19.15 | 19.22 | 16.20 | 16.89 | 17.66 | 2.25 | 0.68 |
| *gltX* | 23.28 | 23.21 | 23.13 | 20.58 | 20.70 | 21.60 | 2.25 | 0.62 |
| *SAUSA300_2125* | 19.48 | 19.61 | 19.41 | 16.78 | 17.08 | 17.92 | 2.24 | 0.65 |
| *ileS* | 23.80 | 23.73 | 23.41 | 20.74 | 21.29 | 22.20 | 2.24 | 0.94 |
| *SAUSA300_1725* | 23.05 | 23.11 | 22.95 | 20.56 | 20.43 | 21.41 | 2.24 | 0.61 |
| *SAUSA300_1899* | 20.28 | 20.12 | 20.03 | 17.57 | 17.80 | 18.36 | 2.24 | 0.53 |
| *SAUSA300_2460* | 22.46 | 22.09 | 21.90 | 19.34 | 19.69 | 20.73 | 2.23 | 0.99 |
| *lpdA* | 24.90 | 24.78 | 24.59 | 22.11 | 22.46 | 23.02 | 2.23 | 0.62 |
| *SAUSA300_0212* | 19.42 | 19.12 | 18.96 | 17.16 | 16.35 | 17.31 | 2.23 | 0.56 |
| *deoB* | 21.78 | 21.73 | 21.56 | 19.24 | 18.86 | 20.29 | 2.23 | 0.84 |
| *SAUSA300_0307* | 27.12 | 26.73 | 27.06 | 24.27 | 24.38 | 25.58 | 2.22 | 0.69 |
| *pyk* | 25.09 | 24.88 | 24.77 | 22.11 | 22.44 | 23.52 | 2.22 | 0.88 |
| *gtaB* | 20.73 | 20.76 | 20.32 | 17.84 | 18.12 | 19.21 | 2.21 | 0.96 |
| *rplS* | 24.98 | 24.66 | 24.15 | 21.89 | 21.95 | 23.29 | 2.21 | 1.20 |
| *ftsY* | 21.92 | 21.46 | 21.68 | 19.08 | 19.37 | 19.99 | 2.21 | 0.58 |
| *fumC* | 20.98 | 20.61 | 20.49 | 17.84 | 18.41 | 19.22 | 2.20 | 0.94 |
| *guaB* | 24.15 | 23.99 | 23.90 | 21.23 | 21.71 | 22.50 | 2.20 | 0.76 |
| *glpK* | 22.96 | 22.71 | 22.65 | 19.92 | 20.45 | 21.35 | 2.20 | 0.88 |
| *fusA* | 26.93 | 26.87 | 26.54 | 23.95 | 24.38 | 25.42 | 2.20 | 0.96 |
| *trxA* | 23.87 | 23.58 | 23.40 | 20.83 | 21.33 | 22.12 | 2.19 | 0.89 |
| *rpmI* | 22.17 | 21.47 | 21.71 | 19.46 | 19.67 | 19.65 | 2.19 | 0.47 |
| *rsbU* | 19.96 | 19.60 | 19.02 | 16.43 | 17.35 | 18.24 | 2.19 | 1.37 |
| *pgi* | 24.67 | 24.60 | 24.44 | 21.91 | 22.40 | 22.89 | 2.17 | 0.61 |
| *gntK* | 18.16 | 17.65 | 17.47 | 15.01 | 15.39 | 16.38 | 2.17 | 1.03 |
| *dnaN* | 23.15 | 22.82 | 22.73 | 20.22 | 20.56 | 21.45 | 2.16 | 0.83 |
| *argF* | 23.30 | 22.71 | 22.31 | 20.26 | 20.51 | 21.08 | 2.16 | 0.91 |
| *accA* | 15.99 | 15.16 | 15.00 | 13.15 | 12.71 | 13.81 | 2.16 | 0.86 |
| *SAUSA300_1449* | 19.64 | 18.52 | 18.67 | 16.42 | 16.68 | 17.26 | 2.16 | 0.94 |
| *zwf* | 22.65 | 22.80 | 22.61 | 20.12 | 20.32 | 21.16 | 2.16 | 0.62 |
| *menD* | 18.54 | 18.86 | 18.34 | 15.89 | 16.52 | 16.87 | 2.15 | 0.61 |
| *topB* | 18.28 | 18.13 | 17.59 | 15.24 | 15.72 | 16.59 | 2.15 | 1.05 |
| *SAUSA300_1171* | 20.04 | 20.34 | 20.18 | 17.68 | 17.78 | 18.64 | 2.15 | 0.54 |
| *trxB* | 24.57 | 24.11 | 24.05 | 21.74 | 21.87 | 22.66 | 2.15 | 0.72 |
| *plc* | 25.52 | 25.14 | 24.74 | 23.41 | 22.47 | 23.07 | 2.15 | 0.50 |
| *SAUSA300_0555* | 22.38 | 22.03 | 22.15 | 19.28 | 20.27 | 20.55 | 2.15 | 0.82 |
| *SAUSA300_0129* | 23.61 | 23.40 | 22.98 | 20.46 | 21.12 | 21.96 | 2.15 | 1.07 |
| *SAUSA300_2254* | 22.44 | 22.19 | 22.33 | 19.61 | 20.20 | 20.71 | 2.15 | 0.61 |
| *SAUSA300_0693* | 23.02 | 22.52 | 22.88 | 20.77 | 20.40 | 20.81 | 2.15 | 0.10 |
| *LukB* | 25.45 | 24.94 | 25.36 | 23.32 | 22.58 | 23.41 | 2.14 | 0.21 |
| *rnj1* | 22.85 | 22.67 | 22.46 | 19.99 | 20.30 | 21.27 | 2.14 | 0.86 |
| *SAUSA300_1136* | 17.95 | 18.07 | 18.03 | 15.55 | 15.71 | 16.40 | 2.14 | 0.43 |
| *ccpA* | 22.42 | 22.24 | 22.31 | 19.82 | 20.17 | 20.58 | 2.13 | 0.44 |
| *SAUSA300_1653* | 23.00 | 22.56 | 22.09 | 20.23 | 20.21 | 20.83 | 2.13 | 0.78 |
| *sepF* | 20.59 | 19.91 | 20.12 | 17.64 | 18.10 | 18.56 | 2.11 | 0.73 |
| *hslU* | 21.31 | 21.19 | 21.37 | 18.88 | 18.85 | 19.82 | 2.11 | 0.48 |
| *SAUSA300_0748* | 16.75 | 16.20 | 15.18 | 13.39 | 14.40 | 14.04 | 2.10 | 1.14 |
| *pepF* | 22.73 | 22.67 | 22.66 | 20.26 | 20.32 | 21.19 | 2.10 | 0.54 |
| *rpsS* | 24.77 | 24.09 | 23.98 | 21.82 | 22.00 | 22.73 | 2.10 | 0.85 |
| *azo1* | 19.58 | 19.52 | 18.60 | 16.46 | 17.63 | 17.32 | 2.09 | 0.94 |
| *dat* | 22.86 | 22.74 | 22.63 | 20.03 | 20.56 | 21.36 | 2.09 | 0.79 |
| *SAUSA300_1336* | 19.69 | 19.41 | 18.93 | 16.96 | 17.18 | 17.62 | 2.09 | 0.72 |
| *moaB* | 19.89 | 19.53 | 19.69 | 17.17 | 17.49 | 18.17 | 2.09 | 0.61 |
| *metB* | 20.59 | 20.02 | 20.33 | 18.06 | 17.83 | 18.78 | 2.09 | 0.50 |
| *rpsK* | 24.00 | 23.41 | 23.31 | 20.95 | 21.22 | 22.28 | 2.09 | 1.01 |
| *LukA* | 24.60 | 24.12 | 24.53 | 22.62 | 21.80 | 22.59 | 2.08 | 0.21 |
| *SAUSA300_2517* | 17.65 | 17.73 | 17.63 | 15.06 | 15.84 | 15.86 | 2.08 | 0.44 |
| *SAUSA300_1895* | 19.26 | 19.25 | 18.79 | 16.49 | 16.81 | 17.75 | 2.08 | 0.92 |
| *dtd* | 19.00 | 18.46 | 18.60 | 16.38 | 16.40 | 17.04 | 2.08 | 0.53 |
| *SAUSA300_1729* | 18.46 | 17.90 | 18.88 | 15.87 | 16.28 | 16.85 | 2.08 | 0.49 |
| *asnS* | 23.62 | 23.67 | 23.65 | 21.12 | 21.50 | 22.08 | 2.08 | 0.47 |
| *murB* | 20.96 | 20.31 | 19.92 | 17.97 | 17.99 | 19.01 | 2.08 | 1.06 |
| *udk* | 20.83 | 19.89 | 19.57 | 17.66 | 17.66 | 18.75 | 2.07 | 1.19 |
| *ezrA* | 18.94 | 19.21 | 19.53 | 16.96 | 17.26 | 17.26 | 2.07 | 0.17 |
| *SAUSA300_2620* | 22.30 | 21.83 | 21.62 | 19.13 | 19.81 | 20.61 | 2.07 | 1.07 |
| *SAUSA300_1011* | 19.24 | 19.03 | 18.92 | 16.69 | 16.80 | 17.54 | 2.06 | 0.61 |
| *traP* | 23.12 | 23.01 | 22.54 | 20.35 | 20.79 | 21.37 | 2.06 | 0.82 |
| *msrB* | 20.73 | 20.07 | 20.44 | 17.64 | 18.42 | 19.02 | 2.05 | 0.90 |
| *tgt* | 19.67 | 19.54 | 19.04 | 16.78 | 17.29 | 18.01 | 2.05 | 0.94 |
| *SAUSA300_0460* | 19.83 | 19.50 | 18.91 | 17.46 | 17.37 | 17.26 | 2.05 | 0.37 |
| *vraR* | 19.20 | 19.97 | 20.28 | 17.40 | 17.70 | 18.19 | 2.05 | 0.23 |
| *SAUSA300_0234* | 21.95 | 22.13 | 21.88 | 19.70 | 19.49 | 20.61 | 2.05 | 0.71 |
| *pbp4* | 16.18 | 16.05 | 16.86 | 14.89 | 13.88 | 14.17 | 2.05 | 0.70 |
| *SAUSA300_1971* | 17.83 | 18.61 | 17.60 | 15.43 | 15.90 | 16.58 | 2.05 | 0.90 |
| *rplP* | 24.78 | 24.49 | 24.29 | 21.83 | 22.45 | 23.15 | 2.04 | 0.90 |
| *fabG* | 22.53 | 22.26 | 22.14 | 19.73 | 20.37 | 20.71 | 2.04 | 0.70 |
| *guaA* | 23.74 | 23.80 | 23.79 | 21.20 | 21.70 | 22.31 | 2.04 | 0.54 |
| *aldA* | 20.75 | 20.27 | 20.12 | 17.93 | 17.86 | 19.24 | 2.04 | 1.02 |
| *pta* | 23.64 | 23.45 | 23.41 | 20.97 | 21.36 | 22.07 | 2.03 | 0.67 |
| *SAUSA300_1902* | 22.95 | 22.83 | 22.63 | 20.27 | 20.62 | 21.43 | 2.03 | 0.76 |
| *SAUSA300_2164* | 19.94 | 19.58 | 20.27 | 18.14 | 17.75 | 17.80 | 2.03 | 0.38 |
| *SAUSA300_1006* | 20.47 | 20.20 | 20.42 | 18.02 | 18.47 | 18.52 | 2.03 | 0.38 |
| *rplO* | 24.45 | 24.03 | 23.80 | 21.73 | 22.03 | 22.44 | 2.03 | 0.68 |
| *pnp* | 23.28 | 23.23 | 22.98 | 20.48 | 21.05 | 21.88 | 2.03 | 0.86 |
| *cmk* | 20.06 | 19.18 | 19.43 | 17.51 | 17.31 | 17.79 | 2.02 | 0.47 |
| *rpsG* | 24.10 | 23.72 | 23.88 | 21.66 | 21.63 | 22.35 | 2.02 | 0.46 |
| *SAUSA300_2453* | 17.71 | 18.08 | 18.77 | 15.45 | 16.25 | 16.80 | 2.02 | 0.22 |
| *metS* | 22.80 | 22.92 | 22.66 | 20.22 | 20.62 | 21.50 | 2.01 | 0.76 |
| *SAUSA300_0618* | 24.54 | 24.19 | 24.40 | 22.36 | 21.97 | 22.78 | 2.01 | 0.34 |
| *SAUSA300_0916* | 21.81 | 21.46 | 21.51 | 19.23 | 19.70 | 19.82 | 2.01 | 0.49 |
| *rplB* | 23.72 | 23.40 | 23.19 | 20.72 | 21.36 | 22.23 | 2.00 | 1.02 |
| *ptpA* | 19.89 | 19.05 | 19.00 | 17.39 | 17.18 | 17.38 | 2.00 | 0.45 |
| *gcvPB* | 20.82 | 20.71 | 20.39 | 18.10 | 18.49 | 19.35 | 1.99 | 0.86 |
| *sucD* | 22.65 | 22.44 | 22.19 | 19.83 | 20.39 | 21.11 | 1.98 | 0.87 |
| *crr* | 23.90 | 23.67 | 23.77 | 21.47 | 21.51 | 22.41 | 1.98 | 0.56 |
| *rnj2* | 22.22 | 22.05 | 21.89 | 19.55 | 19.93 | 20.74 | 1.98 | 0.77 |
| *SAUSA300_1466* | 21.14 | 21.13 | 20.55 | 18.63 | 18.89 | 19.36 | 1.98 | 0.70 |
| *glmM* | 23.07 | 22.78 | 22.69 | 20.44 | 20.67 | 21.50 | 1.98 | 0.73 |
| *nusG* | 23.50 | 23.21 | 23.17 | 20.85 | 21.22 | 21.91 | 1.97 | 0.70 |
| *SAUSA300_1697* | 23.57 | 23.57 | 23.57 | 21.25 | 21.25 | 22.30 | 1.97 | 0.61 |
| *SAUSA300_1759* | 20.23 | 19.99 | 20.42 | 18.58 | 17.85 | 18.33 | 1.96 | 0.27 |
| *folD* | 21.91 | 21.60 | 21.62 | 19.25 | 19.66 | 20.36 | 1.96 | 0.70 |
| *fabD* | 21.34 | 21.31 | 21.54 | 19.33 | 19.11 | 19.91 | 1.95 | 0.29 |
| *argS* | 21.99 | 22.33 | 22.26 | 19.86 | 20.09 | 20.80 | 1.95 | 0.43 |
| *thyA* | 18.84 | 18.51 | 18.80 | 16.71 | 16.14 | 17.46 | 1.94 | 0.54 |
| *rpmE2* | 24.81 | 24.50 | 24.32 | 22.43 | 22.59 | 22.81 | 1.93 | 0.43 |
| *pdhB* | 24.59 | 24.51 | 24.43 | 21.98 | 22.59 | 23.17 | 1.93 | 0.68 |
| *hslV* | 19.28 | 19.45 | 18.81 | 17.13 | 16.58 | 18.05 | 1.93 | 1.07 |
| *rocD* | 19.94 | 19.60 | 19.02 | 17.09 | 17.48 | 18.24 | 1.92 | 1.05 |
| *SAUSA300_0882* | 21.22 | 20.74 | 20.56 | 18.37 | 18.98 | 19.42 | 1.92 | 0.86 |
| *ruvA* | 18.76 | 18.52 | 18.86 | 16.27 | 16.76 | 17.37 | 1.92 | 0.52 |
| *pepS* | 22.22 | 22.13 | 21.86 | 19.75 | 19.93 | 20.78 | 1.92 | 0.74 |
| *polA* | 20.65 | 20.41 | 20.56 | 18.14 | 18.54 | 19.20 | 1.91 | 0.58 |
| *dltA* | 22.69 | 22.94 | 22.82 | 20.35 | 20.91 | 21.46 | 1.91 | 0.50 |
| *rpsQ* | 23.85 | 23.22 | 23.59 | 21.21 | 21.91 | 21.82 | 1.91 | 0.67 |
| *gnd* | 23.85 | 23.73 | 23.64 | 21.33 | 21.71 | 22.46 | 1.91 | 0.68 |
| *codY* | 22.82 | 22.77 | 22.90 | 20.55 | 20.92 | 21.31 | 1.91 | 0.35 |
| *nfo* | 19.99 | 20.07 | 19.78 | 17.49 | 18.20 | 18.45 | 1.90 | 0.58 |
| *mfd* | 19.61 | 18.32 | 18.77 | 16.63 | 17.04 | 17.34 | 1.90 | 0.94 |
| *SAUSA300_1031* | 17.63 | 17.50 | 17.92 | 15.49 | 15.45 | 16.43 | 1.89 | 0.35 |
| *gudB* | 21.16 | 21.13 | 21.15 | 18.89 | 19.10 | 19.77 | 1.89 | 0.46 |
| *cysK* | 24.75 | 24.74 | 24.59 | 22.38 | 22.67 | 23.33 | 1.89 | 0.57 |
| *mnmE* | 20.10 | 20.17 | 20.27 | 18.36 | 18.02 | 18.49 | 1.89 | 0.23 |
| *SAUSA300_1050* | 17.25 | 17.87 | 17.41 | 15.26 | 15.63 | 15.98 | 1.89 | 0.42 |
| *queF* | 21.73 | 20.42 | 20.59 | 18.21 | 19.43 | 19.43 | 1.89 | 1.41 |
| *SAUSA300_0673* | 19.84 | 19.59 | 19.68 | 18.17 | 17.42 | 17.89 | 1.88 | 0.26 |
| *SAUSA300_1720* | 17.20 | 17.21 | 17.35 | 15.13 | 15.50 | 15.50 | 1.87 | 0.18 |
| *ssb* | 21.34 | 21.08 | 20.86 | 18.61 | 19.24 | 19.82 | 1.87 | 0.84 |
| *SAUSA300_0534* | 18.07 | 17.63 | 17.51 | 16.03 | 15.10 | 16.47 | 1.87 | 0.76 |
| *ldh2* | 26.42 | 26.16 | 25.98 | 23.90 | 24.09 | 24.97 | 1.87 | 0.77 |
| *pepT* | 20.29 | 19.74 | 19.74 | 17.70 | 17.85 | 18.62 | 1.87 | 0.74 |
| *SAUSA300_2482* | 21.58 | 21.77 | 21.42 | 19.45 | 19.75 | 19.97 | 1.87 | 0.37 |
| *mtnN* | 20.81 | 20.78 | 20.87 | 18.54 | 18.83 | 19.50 | 1.86 | 0.45 |
| *msrR* | 20.13 | 19.90 | 20.09 | 18.29 | 17.87 | 18.36 | 1.86 | 0.15 |
| *nuc* | 28.73 | 28.83 | 28.77 | 27.26 | 26.53 | 26.96 | 1.86 | 0.42 |
| *trpS* | 21.01 | 21.07 | 21.27 | 18.89 | 19.04 | 19.84 | 1.86 | 0.38 |
| *rplM* | 24.99 | 24.63 | 24.62 | 22.63 | 22.60 | 23.44 | 1.86 | 0.61 |
| *rplE* | 24.34 | 24.07 | 24.31 | 22.04 | 22.24 | 22.87 | 1.86 | 0.43 |
| *SAUSA300_1519* | 17.24 | 17.00 | 16.51 | 14.85 | 14.95 | 15.39 | 1.85 | 0.66 |
| *rpsF* | 22.96 | 22.55 | 22.38 | 20.31 | 20.70 | 21.35 | 1.85 | 0.81 |
| *acpP* | 18.37 | 18.65 | 18.54 | 16.21 | 16.51 | 17.32 | 1.85 | 0.53 |
| *rplF* | 25.14 | 24.88 | 24.63 | 22.46 | 23.05 | 23.60 | 1.84 | 0.83 |
| *glpD* | 23.56 | 23.58 | 23.61 | 21.27 | 21.55 | 22.40 | 1.84 | 0.56 |
| *SAUSA300_1844* | 19.46 | 19.45 | 19.89 | 17.14 | 18.06 | 18.06 | 1.84 | 0.47 |
| *ybeY* | 18.60 | 18.28 | 18.22 | 16.21 | 16.19 | 17.18 | 1.84 | 0.71 |
| *nagD* | 22.44 | 22.07 | 22.12 | 19.94 | 20.17 | 20.99 | 1.84 | 0.69 |
| *SAUSA300_0777* | 19.93 | 19.35 | 19.92 | 17.37 | 18.24 | 18.09 | 1.84 | 0.73 |
| *rplU* | 26.69 | 26.12 | 25.76 | 23.93 | 24.35 | 24.78 | 1.84 | 0.89 |
| *SAUSA300_2112* | 17.32 | 16.29 | 16.84 | 15.04 | 14.50 | 15.40 | 1.84 | 0.42 |
| *SAUSA300_2136* | 20.59 | 20.03 | 20.36 | 18.23 | 18.24 | 19.02 | 1.83 | 0.51 |
| *SAUSA300_1793* | 16.92 | 16.52 | 17.18 | 14.31 | 15.25 | 15.59 | 1.82 | 0.70 |
| *nagB* | 17.78 | 17.47 | 17.17 | 14.99 | 15.59 | 16.38 | 1.82 | 1.00 |
| *rplA* | 23.84 | 23.25 | 23.23 | 20.98 | 21.52 | 22.39 | 1.81 | 1.01 |
| *SAUSA300_0468* | 19.38 | 19.23 | 19.50 | 17.32 | 17.34 | 18.03 | 1.80 | 0.30 |
| *rpmB* | 22.28 | 21.40 | 21.17 | 19.61 | 19.67 | 20.16 | 1.80 | 0.83 |
| *gmk* | 20.79 | 20.65 | 20.62 | 18.39 | 18.78 | 19.49 | 1.80 | 0.64 |
| *thiD* | 22.26 | 21.67 | 21.65 | 19.78 | 19.72 | 20.68 | 1.80 | 0.76 |
| *SAUSA300_0321* | 18.34 | 18.04 | 17.79 | 15.86 | 16.28 | 16.64 | 1.80 | 0.67 |
| *cspA* | 23.07 | 21.88 | 22.45 | 20.19 | 20.66 | 21.16 | 1.79 | 0.94 |
| *apt* | 20.96 | 21.16 | 20.94 | 18.57 | 19.38 | 19.73 | 1.79 | 0.59 |
| *rpsE* | 25.20 | 24.73 | 24.64 | 22.50 | 23.04 | 23.65 | 1.79 | 0.86 |
| *murA* | 20.97 | 20.79 | 20.45 | 18.55 | 18.60 | 19.69 | 1.79 | 0.90 |
| *sak* | 26.28 | 26.39 | 26.36 | 24.83 | 24.33 | 24.52 | 1.79 | 0.30 |
| *SAUSA300_2130* | 21.72 | 22.04 | 21.90 | 19.85 | 19.98 | 20.46 | 1.79 | 0.32 |
| *azoR* | 18.69 | 18.67 | 19.10 | 16.48 | 16.92 | 17.70 | 1.79 | 0.41 |
| *SAUSA300_0105* | 18.59 | 18.27 | 18.74 | 16.86 | 16.20 | 17.18 | 1.79 | 0.26 |
| *modA* | 22.24 | 21.82 | 22.11 | 20.37 | 19.97 | 20.49 | 1.78 | 0.14 |
| *hla* | 27.83 | 27.37 | 27.61 | 26.20 | 25.57 | 25.69 | 1.78 | 0.15 |
| *SAUSA300_0279* | 17.54 | 17.25 | 17.75 | 16.37 | 15.16 | 15.70 | 1.77 | 0.52 |
| *panB* | 20.02 | 20.03 | 20.09 | 18.14 | 18.00 | 18.70 | 1.77 | 0.33 |
| *rpsM* | 24.54 | 23.89 | 23.87 | 21.82 | 22.48 | 22.70 | 1.77 | 0.84 |
| *ppaC* | 22.91 | 22.94 | 22.78 | 20.70 | 21.00 | 21.65 | 1.76 | 0.56 |
| *SAUSA300_1456* | 19.56 | 19.45 | 19.16 | 17.18 | 17.69 | 18.02 | 1.76 | 0.62 |
| *adk* | 22.22 | 22.03 | 22.25 | 20.06 | 20.24 | 20.93 | 1.75 | 0.42 |
| *ispD* | 20.83 | 20.70 | 21.07 | 18.63 | 19.03 | 19.69 | 1.75 | 0.41 |
| *SAUSA300_2484* | 22.70 | 22.64 | 22.82 | 20.68 | 20.82 | 21.42 | 1.75 | 0.32 |
| *rplI* | 21.83 | 21.17 | 21.46 | 19.23 | 19.79 | 20.19 | 1.75 | 0.74 |
| *efp* | 23.78 | 23.39 | 23.49 | 21.41 | 21.58 | 22.45 | 1.74 | 0.67 |
| *SAUSA300_2359* | 24.04 | 23.72 | 23.84 | 22.01 | 21.81 | 22.55 | 1.74 | 0.40 |
| *SAUSA300_0489* | 19.73 | 19.76 | 19.87 | 17.78 | 18.10 | 18.27 | 1.74 | 0.19 |
| *SAUSA300_0100* | 17.26 | 16.82 | 16.98 | 15.50 | 15.23 | 15.14 | 1.73 | 0.13 |
| *SAUSA300_1061* | 20.12 | 19.96 | 19.37 | 18.20 | 17.61 | 18.44 | 1.73 | 0.73 |
| *accC* | 20.95 | 20.60 | 20.99 | 18.59 | 18.97 | 19.79 | 1.73 | 0.59 |
| *SAUSA300_0385* | 21.60 | 21.44 | 21.24 | 19.58 | 19.64 | 19.90 | 1.72 | 0.35 |
| *frr* | 22.46 | 22.16 | 22.39 | 20.40 | 20.43 | 21.01 | 1.72 | 0.34 |
| *hup* | 23.63 | 23.58 | 23.87 | 21.66 | 22.13 | 22.12 | 1.72 | 0.26 |
| *hisS* | 21.46 | 20.77 | 20.86 | 18.79 | 19.06 | 20.11 | 1.71 | 0.96 |
| *rpoD* | 20.70 | 20.12 | 20.40 | 18.27 | 18.46 | 19.37 | 1.70 | 0.70 |
| *SAUSA300_0636* | 19.96 | 19.78 | 19.47 | 17.86 | 17.69 | 18.55 | 1.70 | 0.68 |
| *SAUSA300_2518* | 19.69 | 20.00 | 19.47 | 17.57 | 17.84 | 18.67 | 1.69 | 0.77 |
| *SAUSA300_0990* | 22.50 | 22.40 | 22.33 | 20.09 | 20.97 | 21.10 | 1.69 | 0.63 |
| *ligA* | 19.75 | 19.55 | 19.81 | 17.78 | 17.76 | 18.49 | 1.69 | 0.33 |
| *SAUSA300_0419* | 20.94 | 20.63 | 20.72 | 19.34 | 18.47 | 19.42 | 1.69 | 0.44 |
| *ptsI* | 23.62 | 23.51 | 23.46 | 21.45 | 21.71 | 22.36 | 1.69 | 0.54 |
| *rpmD* | 22.94 | 22.60 | 22.34 | 20.77 | 21.22 | 20.83 | 1.69 | 0.43 |
| *hchA* | 22.41 | 22.31 | 22.21 | 20.38 | 20.47 | 21.04 | 1.68 | 0.44 |
| *rplC* | 24.43 | 24.15 | 23.48 | 21.83 | 22.17 | 23.03 | 1.68 | 1.11 |
| *SAUSA300_1800* | 18.57 | 17.84 | 17.41 | 15.92 | 16.25 | 16.69 | 1.66 | 0.97 |
| *srrA* | 22.75 | 22.51 | 22.88 | 20.83 | 20.96 | 21.41 | 1.65 | 0.24 |
| *rplV* | 24.73 | 24.57 | 24.59 | 22.53 | 22.97 | 23.46 | 1.64 | 0.54 |
| *rplJ* | 23.67 | 23.39 | 23.50 | 21.47 | 21.89 | 22.30 | 1.63 | 0.52 |
| *rpsB* | 23.67 | 23.53 | 23.75 | 21.71 | 21.96 | 22.39 | 1.63 | 0.31 |
| *dnaJ* | 19.92 | 19.47 | 19.74 | 17.85 | 17.93 | 18.46 | 1.63 | 0.40 |
| *SAUSA300_2431* | 15.39 | 15.19 | 15.65 | 13.36 | 14.22 | 13.76 | 1.63 | 0.58 |
| *xpt* | 18.98 | 19.15 | 18.78 | 16.93 | 17.12 | 17.98 | 1.62 | 0.72 |
| *SAUSA300_0430* | 14.51 | 14.24 | 14.10 | 12.70 | 12.90 | 12.40 | 1.62 | 0.25 |
| *tsf* | 24.91 | 24.66 | 24.86 | 22.76 | 23.19 | 23.62 | 1.62 | 0.48 |
| *opp-1A* | 22.38 | 22.18 | 22.49 | 20.71 | 20.29 | 21.20 | 1.62 | 0.30 |
| *gcvPA* | 20.85 | 20.95 | 20.83 | 18.91 | 19.09 | 19.79 | 1.61 | 0.50 |
| *luxS* | 21.40 | 20.95 | 20.96 | 19.54 | 19.02 | 19.93 | 1.61 | 0.50 |
| *pbpA* | 19.64 | 19.47 | 19.68 | 18.07 | 17.70 | 18.21 | 1.61 | 0.15 |
| *sodA* | 22.28 | 22.46 | 22.79 | 20.59 | 21.11 | 21.02 | 1.60 | 0.22 |
| *fba* | 24.40 | 24.36 | 24.37 | 22.56 | 22.56 | 23.20 | 1.60 | 0.37 |
| *rplT* | 22.80 | 22.34 | 21.97 | 20.58 | 20.55 | 21.19 | 1.60 | 0.74 |
| *sucC* | 22.43 | 22.25 | 22.14 | 20.37 | 20.37 | 21.31 | 1.59 | 0.66 |
| *fruB* | 19.50 | 19.24 | 19.58 | 17.22 | 18.00 | 18.34 | 1.59 | 0.60 |
| *SAUSA300_0790* | 21.39 | 22.00 | 22.15 | 19.75 | 20.61 | 20.43 | 1.58 | 0.17 |
| *SAUSA300_2580* | 18.33 | 19.07 | 18.64 | 16.57 | 17.07 | 17.67 | 1.58 | 0.54 |
| *rpsL* | 23.47 | 22.56 | 22.54 | 21.00 | 21.11 | 21.73 | 1.58 | 0.84 |
| *rpsH* | 22.96 | 23.00 | 22.86 | 20.95 | 21.36 | 21.79 | 1.58 | 0.47 |
| *deoD* | 24.09 | 24.11 | 23.62 | 22.00 | 22.28 | 22.82 | 1.57 | 0.68 |
| *SAUSA300_pUSA010004* | 23.33 | 23.26 | 23.27 | 21.92 | 21.42 | 21.81 | 1.57 | 0.24 |
| *SAUSA300_1894* | 20.56 | 20.98 | 20.65 | 18.77 | 19.01 | 19.72 | 1.56 | 0.56 |
| *SAUSA300_1321* | 19.59 | 19.54 | 19.48 | 17.77 | 17.69 | 18.47 | 1.56 | 0.48 |
| *proC* | 18.43 | 18.14 | 17.76 | 16.08 | 16.54 | 17.06 | 1.55 | 0.82 |
| *SAUSA300_1112* | 18.86 | 18.11 | 18.30 | 16.34 | 16.71 | 17.58 | 1.55 | 0.91 |
| *rplN* | 23.23 | 22.77 | 22.52 | 20.90 | 21.23 | 21.79 | 1.54 | 0.80 |
| *SAUSA300_0073* | 17.88 | 17.74 | 17.81 | 16.26 | 15.88 | 16.72 | 1.52 | 0.40 |
| *rpsJ* | 24.30 | 23.80 | 23.85 | 22.41 | 22.23 | 22.76 | 1.52 | 0.40 |
| *SAUSA300_1728* | 19.41 | 19.26 | 19.38 | 17.58 | 17.62 | 18.30 | 1.52 | 0.39 |
| *SAUSA300_0025* | 21.37 | 20.89 | 21.36 | 19.62 | 19.59 | 19.88 | 1.51 | 0.23 |
| *SAUSA300_1393* | 13.43 | 13.10 | 13.08 | 11.39 | 11.73 | 11.97 | 1.51 | 0.48 |
| *SAUSA300_1183* | 18.73 | 18.45 | 18.40 | 16.69 | 16.74 | 17.63 | 1.51 | 0.66 |
| *rplX* | 24.21 | 23.43 | 23.38 | 21.84 | 22.11 | 22.61 | 1.48 | 0.82 |
| *atpG* | 20.11 | 19.81 | 20.14 | 18.25 | 18.43 | 18.93 | 1.48 | 0.34 |
| *SAUSA300_0377* | 19.24 | 19.07 | 19.28 | 17.81 | 17.51 | 17.88 | 1.46 | 0.09 |
| *rpmG1* | 23.02 | 22.53 | 22.63 | 20.96 | 21.18 | 21.67 | 1.46 | 0.56 |
| *mreC* | 21.52 | 21.20 | 21.49 | 20.13 | 19.79 | 19.92 | 1.46 | 0.10 |
| *SAUSA300_0721* | 21.06 | 20.78 | 20.95 | 19.33 | 19.12 | 19.98 | 1.45 | 0.42 |
| *menB* | 23.41 | 23.25 | 23.11 | 21.54 | 21.57 | 22.30 | 1.45 | 0.57 |
| *atpH* | 17.01 | 17.69 | 17.65 | 15.46 | 15.94 | 16.60 | 1.45 | 0.36 |
| *gpsB* | 19.79 | 20.26 | 20.68 | 18.50 | 18.69 | 19.26 | 1.43 | 0.14 |
| *rpmF* | 21.89 | 20.88 | 21.10 | 19.44 | 19.86 | 20.34 | 1.41 | 0.91 |
| *SAUSA300_2579* | 24.13 | 23.60 | 24.34 | 22.81 | 22.48 | 22.60 | 1.39 | 0.32 |
| *grpE* | 22.30 | 21.87 | 21.73 | 20.19 | 20.43 | 21.12 | 1.38 | 0.76 |
| *rplW* | 22.22 | 21.87 | 22.55 | 20.37 | 20.98 | 21.14 | 1.38 | 0.48 |
| *rplQ* | 24.46 | 24.07 | 23.99 | 22.37 | 22.91 | 23.12 | 1.37 | 0.64 |
| *hysA* | 20.48 | 20.25 | 20.54 | 19.30 | 18.64 | 19.23 | 1.37 | 0.22 |
| *def* | 21.43 | 21.35 | 21.12 | 19.47 | 20.05 | 20.28 | 1.36 | 0.57 |
| *SAUSA300_0544* | 19.91 | 19.86 | 19.91 | 18.45 | 18.33 | 18.83 | 1.36 | 0.24 |
| *mecA* | 24.59 | 24.47 | 24.66 | 23.36 | 22.91 | 23.38 | 1.36 | 0.18 |
| *SAUSA300_1656* | 22.99 | 22.86 | 22.81 | 21.15 | 21.62 | 21.85 | 1.35 | 0.46 |
| *SAUSA300_1333* | 16.66 | 16.87 | 16.46 | 15.21 | 14.97 | 15.77 | 1.35 | 0.61 |
| *rsbW* | 20.98 | 20.93 | 20.80 | 19.28 | 19.38 | 20.09 | 1.32 | 0.53 |
| *SAUSA300_1652* | 21.27 | 21.14 | 21.41 | 19.45 | 20.25 | 20.18 | 1.31 | 0.47 |
| *SAUSA300_0717* | 21.80 | 21.47 | 21.47 | 19.77 | 20.27 | 20.77 | 1.31 | 0.67 |
| *infC* | 21.36 | 22.22 | 21.58 | 20.15 | 20.48 | 20.68 | 1.29 | 0.42 |
| *ebpS* | 19.28 | 18.90 | 19.24 | 17.91 | 17.46 | 18.22 | 1.28 | 0.23 |
| *rpoE* | 20.82 | 20.54 | 20.60 | 19.01 | 19.27 | 19.91 | 1.25 | 0.56 |
| *pyrH* | 18.25 | 18.87 | 18.19 | 16.84 | 16.95 | 17.78 | 1.25 | 0.76 |
| *ltaS* | 22.80 | 22.73 | 22.66 | 21.68 | 21.28 | 21.51 | 1.24 | 0.18 |
| *SAUSA300_1684* | 19.17 | 18.98 | 19.39 | 17.79 | 17.76 | 18.31 | 1.23 | 0.15 |
| *SAUSA300_1118* | 20.98 | 20.80 | 20.67 | 19.19 | 19.63 | 19.98 | 1.21 | 0.55 |
| *seq* | 22.27 | 22.66 | 22.37 | 21.19 | 21.01 | 21.46 | 1.21 | 0.38 |
| *ndk* | 20.31 | 19.91 | 20.24 | 18.70 | 18.98 | 19.14 | 1.21 | 0.36 |
| *ptsH* | 22.34 | 22.09 | 22.13 | 20.71 | 20.82 | 21.45 | 1.20 | 0.48 |
| *SAUSA300_0146* | 19.30 | 18.89 | 19.21 | 18.02 | 17.82 | 18.07 | 1.17 | 0.11 |
| *SAUSA300_0736* | 22.96 | 22.64 | 23.04 | 21.26 | 21.70 | 22.18 | 1.16 | 0.47 |
| *SAUSA300_0320* | 22.75 | 22.50 | 23.07 | 21.75 | 21.33 | 21.77 | 1.16 | 0.15 |
| *fabZ* | 19.88 | 19.66 | 19.60 | 18.59 | 18.74 | 18.33 | 1.15 | 0.21 |
| *rplK* | 22.45 | 22.17 | 22.21 | 20.78 | 21.03 | 21.57 | 1.15 | 0.52 |
| *fni* | 17.11 | 17.44 | 17.22 | 16.06 | 16.04 | 16.25 | 1.14 | 0.23 |
| *SAUSA300_1804* | 19.62 | 19.53 | 20.08 | 18.76 | 18.40 | 18.66 | 1.13 | 0.28 |
| *SAUSA300_0404* | 19.44 | 19.30 | 19.15 | 18.41 | 18.06 | 18.04 | 1.13 | 0.11 |
| *ssaA* | 21.54 | 21.60 | 21.94 | 20.96 | 20.14 | 20.62 | 1.12 | 0.47 |
| *SAUSA300_2327* | 17.39 | 17.83 | 17.75 | 16.49 | 16.19 | 16.91 | 1.12 | 0.45 |
| *SAUSA300_0958* | 21.33 | 21.19 | 20.99 | 19.95 | 20.21 | 20.03 | 1.10 | 0.24 |
| *SAUSA300_0484* | 17.36 | 17.47 | 17.86 | 16.47 | 16.88 | 16.13 | 1.07 | 0.59 |
| *rplR* | 21.54 | 21.86 | 21.52 | 20.43 | 20.56 | 20.92 | 1.00 | 0.36 |
| *SAUSA300_1856* | 20.21 | 20.26 | 19.90 | 18.83 | 19.17 | 19.38 | 1.00 | 0.44 |
| *SAUSA300_0486* | 20.81 | 20.37 | 20.55 | 19.42 | 19.31 | 20.01 | 1.00 | 0.43 |
| *scn* | 23.83 | 24.00 | 24.21 | 23.17 | 22.94 | 22.95 | 0.99 | 0.31 |
| *SAUSA300_1299* | 18.50 | 18.41 | 18.68 | 17.65 | 17.34 | 17.97 | 0.88 | 0.18 |
| *chp* | 23.67 | 23.60 | 23.95 | 23.03 | 22.67 | 22.94 | 0.86 | 0.20 |
| *hlgA* | 20.81 | 20.89 | 20.66 | 20.16 | 19.58 | 20.06 | 0.85 | 0.40 |
| *purD* | 16.32 | 16.69 | 16.87 | 15.56 | 15.85 | 16.10 | 0.79 | 0.04 |
| *isdB* | 18.24 | 18.35 | 18.42 | 17.70 | 17.38 | 17.57 | 0.79 | 0.22 |
| *isdA* | 21.78 | 21.54 | 21.83 | 20.95 | 20.84 | 21.00 | 0.78 | 0.08 |
| *SAUSA300_1797* | 20.30 | 20.56 | 20.64 | 19.54 | 19.65 | 20.01 | 0.77 | 0.14 |
| *copA* | 17.07 | 17.08 | 17.60 | 16.60 | 16.57 | 16.34 | 0.75 | 0.45 |
| *SAUSA300_0400* | 18.63 | 18.21 | 18.36 | 17.82 | 17.45 | 17.70 | 0.74 | 0.08 |
| *sdrE* | 19.39 | 19.12 | 19.37 | 18.55 | 18.42 | 18.70 | 0.74 | 0.09 |
| *SAUSA300_2578* | 16.99 | 16.59 | 16.83 | 16.27 | 15.96 | 16.07 | 0.70 | 0.06 |
| *SAUSA300_1988* | 19.55 | 19.93 | 19.90 | 19.23 | 18.85 | 19.28 | 0.68 | 0.39 |
| *SAUSA300_0114* | 19.26 | 19.51 | 19.52 | 18.85 | 18.83 | 19.03 | 0.53 | 0.14 |
| *sek* | 22.90 | 22.88 | 22.88 | 22.44 | 22.35 | 22.43 | 0.48 | 0.04 |
| *SAUSA300_2287* | 16.15 | 16.05 | 16.18 | 15.98 | 15.94 | 15.91 | 0.18 | 0.08 |
| *fmtB* | 20.17 | 20.03 | 20.11 | 20.57 | 20.32 | 20.46 | -0.35 | 0.06 |
| *SAUSA300_0408* | 22.66 | 22.82 | 22.91 | 24.10 | 23.95 | 24.10 | -1.26 | 0.16 |
| *SAUSA300_1052* | 22.25 | 22.48 | 22.28 | 23.84 | 23.86 | 23.63 | -1.44 | 0.13 |
| *coa* | 19.34 | 19.52 | 19.79 | 21.70 | 20.77 | 21.20 | -1.67 | 0.60 |
| *SAUSA300_0773* | 17.53 | 17.39 | 17.59 | 19.78 | 19.31 | 19.59 | -2.05 | 0.17 |
| *SAUSA300_0812* | 19.06 | 18.85 | 19.39 | 20.82 | 21.36 | 21.50 | -2.12 | 0.38 |
| *SAUSA300_1937* | 15.29 | 15.16 | 14.25 | 16.96 | 17.45 | 17.19 | -2.30 | 0.63 |
| *efb* | 18.21 | 18.78 | 18.53 | 20.74 | 20.89 | 20.91 | -2.34 | 0.22 |
| *SAUSA300_1960* | 16.24 | 16.52 | 15.09 | 18.11 | 18.43 | 19.11 | -2.60 | 1.23 |
| *SAUSA300_1926* | 14.82 | 16.30 | 14.62 | 17.04 | 18.66 | 18.53 | -2.83 | 0.94 |
| *SAUSA300_1934* | 14.59 | 14.09 | 15.25 | 19.27 | 20.12 | 20.08 | -5.18 | 0.74 |
