# Supplementary material for: Staphylococcus aureus Responds to the Central Metabolite Pyruvate To Regulate Virulence
Source: mBio. 2018 Jan 23;9(1):e02272-17. doi: 10.1128/mBio.02272-17 (PMC5784258; doi:10.1128/mBio.02272-17)
Supplement: FIG S2 [file mbo001183696sf2.pdf]

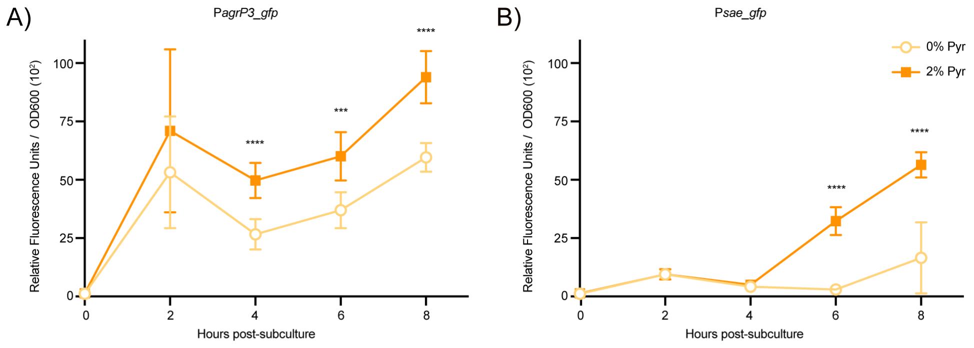

Supplemental Figure 2: Pyruvate activates the expression of master regulators, Sae and Agr. A – B) The promoter activity of USA300 was examined in the absence or presence of 2% pyruvate using promoter reporter strains driving the expression of GFP from the *agrP3* (A) or *sae* (B) promoter over time. The average relative fluorescence of 3 independent experiments are shown +/- standard deviation. Analysis of statistical significance was determined by a student t-test. ***, $P < 0.005$; ****, $P < 0.0001$.
