# Supplementary material for: Staphylococcus aureus Responds to the Central Metabolite Pyruvate To Regulate Virulence
Source: mBio. 2018 Jan 23;9(1):e02272-17. doi: 10.1128/mBio.02272-17 (PMC5784258; doi:10.1128/mBio.02272-17)
Supplement: FIG S3 [file mbo001183696sf3.pdf]

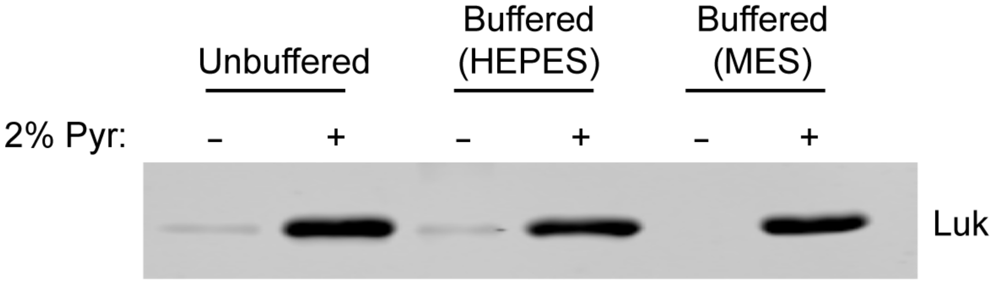

pH at 0hrs	6.96 ± 0.08	6.80 ± 0.06	7.23 ± 0.12	7.30 ± 0.10	5.41 ± 0.01	5.43 ± 0.06
pH at 5hrs	5.91 ± 0.03	6.48 ± 0.10	6.39 ± 0.07	6.42 ± 0.05	5.35 ± 0.04	6.12 ± 0.03

Supplemental Figure 3: The pH of the culture does not affect the ability of pyruvate to induce leukocidin production. Representative Western blot of F-type leukocidins (Luk) is shown for protein isolated from the cultured supernatants of USA300 at post-exponential growth phase. YC media +/- pyruvate was tested without a buffer, or buffered to either pH ~7.2 in HEPES or pH ~5.4 in MES. The pH of 3 independent experiments were measured at the start of the culture and at post-exponential growth phase (5hrs). The pH values are shown +/- standard deviation.
