# Supplementary material for: Staphylococcus aureus Responds to the Central Metabolite Pyruvate To Regulate Virulence
Source: mBio. 2018 Jan 23;9(1):e02272-17. doi: 10.1128/mBio.02272-17 (PMC5784258; doi:10.1128/mBio.02272-17)
Supplement: TABLE S2 [file mbo001183696st2.docx]

**Supplemental Table 2:** Genes that significantly changed in relative mRNA levels in the presence of pyruvate.

| **Gene Name** | **YC 1  Norm. Counts** | **YC 2  Norm. Counts** | **YCP 1  Norm. Counts** | **YCP 2  Norm. Counts** | **Fold Change1 (YCP/YC)** | **Fold Change 2 (YCP/YC)** | **Ave Fold Change (Relative to Pyr)** | **Standard Deviation** |
| --- | --- | --- | --- | --- | --- | --- | --- | --- |
| *pyrP* | 9.61 | 12.31 | 1851.10 | 1976.85 | 192.52 | 160.63 | 176.58 | 22.55 |
| *SAUSA300_1379*† | 0.00 | 0.00 | 16.49 | 12.99 | - | - | 175.00 | - |
| *pyrB* | 13.22 | 16.70 | 2133.35 | 2301.61 | 161.37 | 137.80 | 149.58 | 16.66 |
| *pyrC* | 27.64 | 27.25 | 2663.02 | 2912.17 | 96.34 | 106.87 | 101.60 | 7.45 |
| *carA* | 33.65 | 35.16 | 2567.71 | 2753.22 | 76.30 | 78.30 | 77.30 | 1.41 |
| *SAUSA300_1380* | 1.20 | 0.88 | 81.56 | 64.95 | 67.86 | 73.89 | 70.87 | 4.26 |
| *SAUSA300_0432* | 30.05 | 24.61 | 1968.40 | 1834.72 | 65.51 | 74.54 | 70.03 | 6.39 |
| *carB* | 139.42 | 130.98 | 7836.02 | 8186.32 | 56.21 | 62.50 | 59.35 | 4.45 |
| *lukS-PV* | 241.58 | 250.53 | 14280.05 | 12680.27 | 59.11 | 50.61 | 54.86 | 6.01 |
| *pyrF* | 32.45 | 26.37 | 1196.80 | 1221.11 | 36.88 | 46.30 | 41.59 | 6.66 |
| *pyrE* | 32.45 | 29.89 | 1241.70 | 1318.92 | 38.26 | 44.13 | 41.20 | 4.15 |
| *lukF-PV* | 231.96 | 304.15 | 10918.74 | 9609.16 | 47.07 | 31.59 | 39.33 | 10.94 |
| *fabH* | 37.26 | 53.62 | 1487.30 | 1493.91 | 39.92 | 27.86 | 33.89 | 8.53 |
| *SAUSA300_0561* | 1.20 | 2.64 | 58.65 | 45.85 | 48.80 | 17.39 | 33.09 | 22.21 |
| *SAUSA300_2493* | 10.82 | 10.55 | 274.00 | 207.08 | 25.33 | 19.63 | 22.48 | 4.03 |
| *SAUSA300_1334* | 104.56 | 112.52 | 2010.55 | 2144.20 | 19.23 | 19.06 | 19.14 | 0.12 |
| *lukE* | 2.40 | 2.64 | 44.90 | 50.43 | 18.68 | 19.12 | 18.90 | 0.31 |
| *SAUSA300_0355* | 241.58 | 200.42 | 2934.27 | 3024.50 | 12.15 | 15.09 | 13.62 | 2.08 |
| *SAUSA300_2133* | 117.78 | 117.79 | 1729.22 | 1437.36 | 14.68 | 12.20 | 13.44 | 1.75 |
| *pyrD* | 263.21 | 293.60 | 3249.51 | 3479.93 | 12.35 | 11.85 | 12.10 | 0.35 |
| *prsA* | 429.07 | 371.84 | 4877.92 | 4555.85 | 11.37 | 12.25 | 11.81 | 0.62 |
| *ear* | 76.92 | 70.32 | 943.88 | 750.39 | 12.27 | 10.67 | 11.47 | 1.13 |
| *tyrS* | 70.91 | 84.39 | 827.50 | 678.56 | 11.67 | 8.04 | 9.86 | 2.57 |
| *SAUSA300_1674* | 48.07 | 63.29 | 542.50 | 522.68 | 11.28 | 8.26 | 9.77 | 2.14 |
| *SAUSA300_0932* | 126.20 | 137.13 | 1316.85 | 1243.27 | 10.43 | 9.07 | 9.75 | 0.97 |
| *empbp* | 8.41 | 3.52 | 37.57 | 52.73 | 4.47 | 15.00 | 9.73 | 7.45 |
| *splA* | 84.13 | 75.60 | 743.19 | 769.50 | 8.83 | 10.18 | 9.51 | 0.95 |
| *msrR* | 94.95 | 75.60 | 816.50 | 787.07 | 8.60 | 10.41 | 9.51 | 1.28 |
| *SAUSA300_0931* | 51.68 | 44.83 | 416.96 | 463.07 | 8.07 | 10.33 | 9.20 | 1.60 |
| *SAUSA300_1099* | 49.28 | 56.26 | 470.11 | 458.49 | 9.54 | 8.15 | 8.84 | 0.98 |
| *SAUSA300_0307* | 80.53 | 87.03 | 761.52 | 698.43 | 9.46 | 8.03 | 8.74 | 1.01 |
| *SAUSA300_1665* | 61.30 | 43.95 | 411.46 | 458.49 | 6.71 | 10.43 | 8.57 | 2.63 |
| *pyrG* | 530.03 | 562.60 | 4772.54 | 4522.23 | 9.00 | 8.04 | 8.52 | 0.68 |
| *SAUSA300_0775* | 10.82 | 5.27 | 94.39 | 39.74 | 8.73 | 7.53 | 8.13 | 0.84 |
| *splB* | 58.89 | 55.38 | 467.36 | 447.79 | 7.94 | 8.09 | 8.01 | 0.11 |
| *hisS* | 51.68 | 65.93 | 468.27 | 454.67 | 9.06 | 6.90 | 7.98 | 1.53 |
| *SAUSA300_0755* | 677.86 | 685.66 | 5357.20 | 5400.23 | 7.90 | 7.88 | 7.89 | 0.02 |
| *SAUSA300_0783* | 74.52 | 86.15 | 555.33 | 660.22 | 7.45 | 7.66 | 7.56 | 0.15 |
| *acnA* | 1586.47 | 1419.68 | 10926.99 | 11374.34 | 6.89 | 8.01 | 7.45 | 0.80 |
| *SAUSA300_2446* | 28.84 | 25.49 | 212.60 | 190.27 | 7.37 | 7.46 | 7.42 | 0.07 |
| *SAUSA300_2484* | 687.47 | 641.71 | 4656.16 | 4997.53 | 6.77 | 7.79 | 7.28 | 0.72 |
| *sspA* | 10.82 | 14.06 | 89.81 | 87.11 | 8.30 | 6.19 | 7.25 | 1.49 |
| *fabF* | 552.86 | 568.75 | 4017.44 | 4081.31 | 7.27 | 7.18 | 7.22 | 0.06 |
| *splC* | 46.87 | 72.96 | 440.78 | 364.50 | 9.40 | 5.00 | 7.20 | 3.12 |
| *sspC* | 4.81 | 7.91 | 44.90 | 38.97 | 9.34 | 4.93 | 7.13 | 3.12 |
| *SAUSA300_2527* | 124.99 | 140.65 | 940.21 | 930.73 | 7.52 | 6.62 | 7.07 | 0.64 |
| *SAUSA300_1296* | 14.42 | 16.70 | 89.81 | 125.32 | 6.23 | 7.50 | 6.87 | 0.90 |
| *isaA* | 74.52 | 79.12 | 490.27 | 555.54 | 6.58 | 7.02 | 6.80 | 0.31 |
| *SAUSA300_0569* | 733.14 | 730.50 | 4875.18 | 5051.78 | 6.65 | 6.92 | 6.78 | 0.19 |
| *SAUSA300_0076* | 6.01 | 19.34 | 58.65 | 73.36 | 9.76 | 3.79 | 6.78 | 4.22 |
| *SAUSA300_1241* | 81.73 | 124.83 | 658.88 | 621.25 | 8.06 | 4.98 | 6.52 | 2.18 |
| *pyrR* | 157.45 | 172.30 | 966.79 | 1146.99 | 6.14 | 6.66 | 6.40 | 0.37 |
| *ribD* | 40.86 | 25.49 | 185.11 | 209.38 | 4.53 | 8.21 | 6.37 | 2.60 |
| *SAUSA300_2317* | 58.89 | 56.26 | 346.39 | 378.25 | 5.88 | 6.72 | 6.30 | 0.60 |
| *splD* | 36.06 | 72.96 | 308.82 | 291.14 | 8.57 | 3.99 | 6.28 | 3.23 |
| *SAUSA300_1121* | 302.87 | 366.57 | 1944.57 | 2134.27 | 6.42 | 5.82 | 6.12 | 0.42 |
| *SAUSA300_0846* | 207.92 | 200.42 | 1183.97 | 1282.24 | 5.69 | 6.40 | 6.05 | 0.50 |
| *SAUSA300_0257* | 1954.25 | 1995.46 | 12396.88 | 11249.79 | 6.34 | 5.64 | 5.99 | 0.50 |
| *SAUSA300_2495* | 543.25 | 498.43 | 3237.59 | 3000.81 | 5.96 | 6.02 | 5.99 | 0.04 |
| *SAUSA300_1668* | 48.07 | 54.50 | 326.23 | 276.62 | 6.79 | 5.08 | 5.93 | 1.21 |
| *SAUSA300_2471* | 93.75 | 98.45 | 530.59 | 609.79 | 5.66 | 6.19 | 5.93 | 0.38 |
| *sdaAB* | 44.47 | 36.92 | 252.01 | 227.72 | 5.67 | 6.17 | 5.92 | 0.35 |
| *SAUSA300_0215* | 8.41 | 15.82 | 63.23 | 64.95 | 7.52 | 4.10 | 5.81 | 2.41 |
| *SAUSA300_0672* | 3104.44 | 3458.21 | 18939.87 | 19022.70 | 6.10 | 5.50 | 5.80 | 0.42 |
| *rpsT* | 567.28 | 632.04 | 3651.80 | 3166.63 | 6.44 | 5.01 | 5.72 | 1.01 |
| *SAUSA300_0424* | 25.24 | 21.98 | 128.29 | 139.84 | 5.08 | 6.36 | 5.72 | 0.91 |
| *SAUSA300_1238* | 10.82 | 27.25 | 91.64 | 78.71 | 8.47 | 2.89 | 5.68 | 3.95 |
| *SAUSA300_1026* | 1650.17 | 1548.02 | 8817.47 | 9185.82 | 5.34 | 5.93 | 5.64 | 0.42 |
| *SAUSA300_2037* | 1355.71 | 1330.89 | 8019.30 | 7087.47 | 5.92 | 5.33 | 5.62 | 0.42 |
| *splE* | 38.46 | 57.14 | 263.00 | 249.88 | 6.84 | 4.37 | 5.61 | 1.74 |
| *SAUSA300_2262* | 44.47 | 69.45 | 285.00 | 318.65 | 6.41 | 4.59 | 5.50 | 1.29 |
| *acpP* | 925.44 | 1115.52 | 6181.03 | 4733.13 | 6.68 | 4.24 | 5.46 | 1.72 |
| *plsX* | 536.04 | 644.35 | 3081.81 | 3291.95 | 5.75 | 5.11 | 5.43 | 0.45 |
| *coaA* | 181.48 | 167.02 | 941.13 | 942.96 | 5.19 | 5.65 | 5.42 | 0.33 |
| *SAUSA300_2206* | 131.00 | 144.17 | 745.94 | 737.40 | 5.69 | 5.11 | 5.40 | 0.41 |
| *chs* | 75.72 | 72.96 | 420.62 | 382.07 | 5.56 | 5.24 | 5.40 | 0.23 |
| *tpx* | 200.71 | 214.49 | 1114.33 | 1106.49 | 5.55 | 5.16 | 5.36 | 0.28 |
| *fhs* | 814.87 | 847.41 | 4130.15 | 4474.85 | 5.07 | 5.28 | 5.17 | 0.15 |
| *SAUSA300_2530* | 31.25 | 60.65 | 230.93 | 175.75 | 7.39 | 2.90 | 5.14 | 3.18 |
| *tig* | 1519.17 | 1460.99 | 7674.74 | 7597.16 | 5.05 | 5.20 | 5.13 | 0.10 |
| *SAUSA300_0875* | 326.91 | 404.37 | 1906.08 | 1782.76 | 5.83 | 4.41 | 5.12 | 1.01 |
| *SAUSA300_1022* | 19.23 | 27.25 | 112.72 | 117.68 | 5.86 | 4.32 | 5.09 | 1.09 |
| *sdaAA* | 114.18 | 123.95 | 618.56 | 589.16 | 5.42 | 4.75 | 5.09 | 0.47 |
| *SAUSA300_2515* | 10.82 | 9.67 | 54.07 | 49.67 | 5.00 | 5.14 | 5.07 | 0.10 |
| *argG* | 20.43 | 14.94 | 87.97 | 86.35 | 4.31 | 5.78 | 5.04 | 1.04 |
| *SAUSA300_1607* | 70.91 | 68.57 | 354.64 | 345.39 | 5.00 | 5.04 | 5.02 | 0.03 |
| *aspS* | 455.51 | 479.97 | 2309.29 | 2378.03 | 5.07 | 4.95 | 5.01 | 0.08 |
| *copA* | 1838.87 | 1612.19 | 8856.87 | 8239.81 | 4.82 | 5.11 | 4.96 | 0.21 |
| *SAUSA300_0442* | 98.55 | 100.21 | 533.34 | 450.08 | 5.41 | 4.49 | 4.95 | 0.65 |
| *ureC* | 46.87 | 40.44 | 184.19 | 235.36 | 3.93 | 5.82 | 4.88 | 1.34 |
| *SAUSA300_0639* | 760.79 | 707.64 | 3442.86 | 3654.92 | 4.53 | 5.16 | 4.85 | 0.45 |
| *SAUSA300_0943* | 3.61 | 2.64 | 13.75 | 15.28 | 3.81 | 5.80 | 4.80 | 1.40 |
| *SAUSA300_2002* | 132.21 | 147.68 | 683.62 | 629.66 | 5.17 | 4.26 | 4.72 | 0.64 |
| *SAUSA300_0072* | 18.03 | 12.31 | 59.57 | 74.12 | 3.30 | 6.02 | 4.66 | 1.92 |
| *SAUSA300_0992* | 170.67 | 167.90 | 942.05 | 635.77 | 5.52 | 3.79 | 4.65 | 1.23 |
| *rimI* | 13.22 | 21.98 | 73.31 | 82.53 | 5.55 | 3.76 | 4.65 | 1.27 |
| *SAUSA300_1451* | 48.07 | 41.32 | 175.95 | 231.54 | 3.66 | 5.60 | 4.63 | 1.37 |
| *SAUSA300_0697* | 108.17 | 126.58 | 550.75 | 525.73 | 5.09 | 4.15 | 4.62 | 0.66 |
| *SAUSA300_2100* | 663.43 | 722.58 | 3296.24 | 3039.02 | 4.97 | 4.21 | 4.59 | 0.54 |
| *SAUSA300_2349* | 521.61 | 676.87 | 2684.10 | 2596.57 | 5.15 | 3.84 | 4.49 | 0.93 |
| *SAUSA300_2448* | 201.91 | 237.35 | 981.45 | 970.47 | 4.86 | 4.09 | 4.47 | 0.55 |
| *rpsD* | 3910.90 | 3910.92 | 16607.67 | 18046.88 | 4.25 | 4.61 | 4.43 | 0.26 |
| *typA* | 372.58 | 430.74 | 1644.00 | 1906.55 | 4.41 | 4.43 | 4.42 | 0.01 |
| *SAUSA300_0883* | 137.01 | 134.50 | 637.80 | 560.88 | 4.66 | 4.17 | 4.41 | 0.34 |
| *SAUSA300_0071* | 37.26 | 51.86 | 185.11 | 197.91 | 4.97 | 3.82 | 4.39 | 0.81 |
| *SAUSA300_0730* | 312.49 | 273.39 | 1218.79 | 1321.98 | 3.90 | 4.84 | 4.37 | 0.66 |
| *SAUSA300_1725* | 207.92 | 236.47 | 1040.10 | 864.25 | 5.00 | 3.65 | 4.33 | 0.95 |
| *SAUSA300_0669* | 175.47 | 199.55 | 793.59 | 820.69 | 4.52 | 4.11 | 4.32 | 0.29 |
| *SAUSA300_0953* | 12.02 | 11.43 | 44.90 | 55.78 | 3.74 | 4.88 | 4.31 | 0.81 |
| *SAUSA300_2127* | 117.78 | 123.07 | 527.84 | 505.87 | 4.48 | 4.11 | 4.30 | 0.26 |
| *SAUSA300_2475* | 36.06 | 21.10 | 108.13 | 117.68 | 3.00 | 5.58 | 4.29 | 1.82 |
| *tetR* | 222.35 | 254.05 | 1019.94 | 1001.03 | 4.59 | 3.94 | 4.26 | 0.46 |
| *SAUSA300_0230* | 38.46 | 48.35 | 186.03 | 176.52 | 4.84 | 3.65 | 4.24 | 0.84 |
| *ribE* | 14.42 | 14.06 | 59.57 | 61.13 | 4.13 | 4.35 | 4.24 | 0.15 |
| *SAUSA300_2492* | 18.03 | 11.43 | 66.90 | 54.25 | 3.71 | 4.75 | 4.23 | 0.73 |
| *SAUSA300_1747* | 14.42 | 31.65 | 89.81 | 68.77 | 6.23 | 2.17 | 4.20 | 2.87 |
| *pepF* | 361.76 | 432.50 | 1765.88 | 1509.19 | 4.88 | 3.49 | 4.19 | 0.98 |
| *SAUSA300_0917* | 139.42 | 173.17 | 599.32 | 697.67 | 4.30 | 4.03 | 4.16 | 0.19 |
| *SAUSA300_2621* | 145.43 | 123.95 | 543.42 | 564.71 | 3.74 | 4.56 | 4.15 | 0.58 |
| *SAUSA300_0079* | 451.90 | 426.34 | 2029.80 | 1619.23 | 4.49 | 3.80 | 4.14 | 0.49 |
| *hslO* | 174.27 | 203.94 | 775.26 | 780.96 | 4.45 | 3.83 | 4.14 | 0.44 |
| *fabD* | 782.42 | 662.81 | 2893.95 | 3022.20 | 3.70 | 4.56 | 4.13 | 0.61 |
| *pcrB* | 22.84 | 38.68 | 110.88 | 130.67 | 4.86 | 3.38 | 4.12 | 1.04 |
| *SAUSA300_2474* | 66.10 | 49.23 | 230.93 | 233.07 | 3.49 | 4.73 | 4.11 | 0.88 |
| *SAUSA300_1759* | 2.40 | 3.52 | 10.08 | 13.75 | 4.19 | 3.91 | 4.05 | 0.20 |
| *gatB* | 1162.21 | 1158.60 | 4724.89 | 4642.96 | 4.07 | 4.01 | 4.04 | 0.04 |
| *ribBA* | 27.64 | 28.13 | 111.80 | 113.09 | 4.04 | 4.02 | 4.03 | 0.02 |
| *rumA* | 200.71 | 163.50 | 846.74 | 622.02 | 4.22 | 3.80 | 4.01 | 0.29 |
| *SAUSA300_0789* | 51.68 | 44.83 | 218.10 | 170.40 | 4.22 | 3.80 | 4.01 | 0.30 |
| *SAUSA300_2540* | 1201.87 | 1391.55 | 5169.34 | 5175.57 | 4.30 | 3.72 | 4.01 | 0.41 |
| *SAUSA300_1248* | 173.07 | 166.14 | 690.04 | 668.63 | 3.99 | 4.02 | 4.01 | 0.03 |
| *ccrB* | 74.52 | 101.09 | 350.06 | 334.70 | 4.70 | 3.31 | 4.00 | 0.98 |
| *fabG* | 930.25 | 915.98 | 3682.04 | 3690.07 | 3.96 | 4.03 | 3.99 | 0.05 |
| *SAUSA300_1324* | 27.64 | 16.70 | 84.31 | 81.76 | 3.05 | 4.90 | 3.97 | 1.31 |
| *SAUSA300_0987* | 88.94 | 101.97 | 391.30 | 360.68 | 4.40 | 3.54 | 3.97 | 0.61 |
| *SAUSA300_1795* | 612.96 | 580.18 | 2548.47 | 2164.83 | 4.16 | 3.73 | 3.94 | 0.30 |
| *opp-3B* | 38.46 | 43.95 | 150.29 | 174.23 | 3.91 | 3.96 | 3.94 | 0.04 |
| *clpX* | 1134.57 | 1118.16 | 4428.90 | 4435.11 | 3.90 | 3.97 | 3.94 | 0.04 |
| *SAUSA300_2318* | 7.21 | 4.40 | 20.16 | 22.16 | 2.80 | 5.04 | 3.92 | 1.59 |
| *lukD* | 9.61 | 9.67 | 40.32 | 35.15 | 4.19 | 3.64 | 3.91 | 0.39 |
| *fdhD* | 70.91 | 94.06 | 327.15 | 301.07 | 4.61 | 3.20 | 3.91 | 1.00 |
| *SAUSA300_1606* | 164.66 | 124.83 | 613.98 | 501.28 | 3.73 | 4.02 | 3.87 | 0.20 |
| *def* | 133.41 | 162.63 | 568.16 | 566.23 | 4.26 | 3.48 | 3.87 | 0.55 |
| *SAUSA300_1738* | 4.81 | 9.67 | 23.83 | 26.75 | 4.96 | 2.77 | 3.86 | 1.55 |
| *SAUSA300_0986* | 132.21 | 145.04 | 529.67 | 532.61 | 4.01 | 3.67 | 3.84 | 0.24 |
| *glnA* | 3170.55 | 2711.89 | 11517.14 | 10956.35 | 3.63 | 4.04 | 3.84 | 0.29 |
| *SAUSA300_1780* | 122.59 | 118.67 | 459.11 | 465.37 | 3.75 | 3.92 | 3.83 | 0.12 |
| *opuD* | 961.50 | 1122.56 | 3935.88 | 3989.62 | 4.09 | 3.55 | 3.82 | 0.38 |
| *purA* | 93.75 | 111.64 | 407.79 | 360.68 | 4.35 | 3.23 | 3.79 | 0.79 |
| *accB* | 185.09 | 214.49 | 732.19 | 768.73 | 3.96 | 3.58 | 3.77 | 0.26 |
| *SAUSA300_2494* | 2521.53 | 2462.24 | 9434.20 | 9321.84 | 3.74 | 3.79 | 3.76 | 0.03 |
| *hysA* | 21.63 | 23.73 | 77.89 | 92.46 | 3.60 | 3.90 | 3.75 | 0.21 |
| *SAUSA300_1462* | 241.58 | 218.89 | 811.92 | 901.69 | 3.36 | 4.12 | 3.74 | 0.54 |
| *frr* | 413.44 | 412.28 | 1567.94 | 1516.83 | 3.79 | 3.68 | 3.74 | 0.08 |
| *SAUSA300_1375* | 6.01 | 5.27 | 23.83 | 18.34 | 3.96 | 3.48 | 3.72 | 0.34 |
| *dltC* | 149.03 | 164.38 | 607.56 | 552.48 | 4.08 | 3.36 | 3.72 | 0.51 |
| *SAUSA300_2409* | 13.22 | 30.77 | 58.65 | 91.70 | 4.44 | 2.98 | 3.71 | 1.03 |
| *SAUSA300_0025* | 45.67 | 73.84 | 202.52 | 218.55 | 4.43 | 2.96 | 3.70 | 1.04 |
| *SAUSA300_0945* | 40.86 | 68.57 | 186.94 | 191.04 | 4.57 | 2.79 | 3.68 | 1.26 |
| *rpmF* | 896.60 | 833.35 | 3165.20 | 3191.85 | 3.53 | 3.83 | 3.68 | 0.21 |
| *rnhC* | 24.04 | 36.04 | 107.22 | 103.16 | 4.46 | 2.86 | 3.66 | 1.13 |
| *SAUSA300_2237* | 7.21 | 21.98 | 33.91 | 57.31 | 4.70 | 2.61 | 3.65 | 1.48 |
| *rplA* | 3577.98 | 3500.40 | 12706.61 | 12963.00 | 3.55 | 3.70 | 3.63 | 0.11 |
| *ipdC* | 203.12 | 150.32 | 583.74 | 656.40 | 2.87 | 4.37 | 3.62 | 1.06 |
| *rpsJ* | 1968.67 | 2015.68 | 7021.35 | 7321.30 | 3.57 | 3.63 | 3.60 | 0.05 |
| *SAUSA300_0847* | 191.10 | 200.42 | 675.38 | 733.58 | 3.53 | 3.66 | 3.60 | 0.09 |
| *SAUSA300_0408* | 26.44 | 36.92 | 108.13 | 113.86 | 4.09 | 3.08 | 3.59 | 0.71 |
| *dltD* | 1395.38 | 1610.43 | 5238.98 | 5398.70 | 3.75 | 3.35 | 3.55 | 0.28 |
| *SAUSA300_0947* | 197.11 | 173.17 | 640.55 | 667.87 | 3.25 | 3.86 | 3.55 | 0.43 |
| *SAUSA300_0694* | 155.04 | 204.82 | 654.30 | 579.99 | 4.22 | 2.83 | 3.53 | 0.98 |
| *menA* | 158.65 | 128.34 | 501.26 | 495.93 | 3.16 | 3.86 | 3.51 | 0.50 |
| *SAUSA300_1240* | 368.98 | 319.10 | 1291.19 | 1117.95 | 3.50 | 3.50 | 3.50 | 0.00 |
| *SAUSA300_2005* | 7.21 | 16.70 | 30.24 | 46.61 | 4.19 | 2.79 | 3.49 | 0.99 |
| *oxaA* | 1249.95 | 1231.56 | 4320.76 | 4338.83 | 3.46 | 3.52 | 3.49 | 0.05 |
| *glcU* | 180.28 | 226.80 | 680.88 | 719.83 | 3.78 | 3.17 | 3.48 | 0.43 |
| *SAUSA300_0443* | 61.30 | 49.23 | 189.69 | 189.51 | 3.09 | 3.85 | 3.47 | 0.53 |
| *cdr* | 329.31 | 367.45 | 1241.70 | 1163.80 | 3.77 | 3.17 | 3.47 | 0.43 |
| *SAUSA300_0264* | 318.50 | 295.36 | 985.12 | 1134.76 | 3.09 | 3.84 | 3.47 | 0.53 |
| *SAUSA300_0922* | 182.68 | 207.46 | 687.29 | 650.29 | 3.76 | 3.13 | 3.45 | 0.44 |
| *glnR* | 597.33 | 475.57 | 1924.41 | 1726.97 | 3.22 | 3.63 | 3.43 | 0.29 |
| *SAUSA300_1919* | 593.73 | 623.25 | 2257.98 | 1888.97 | 3.80 | 3.03 | 3.42 | 0.55 |
| *SAUSA300_1295* | 2427.79 | 2587.06 | 9239.01 | 7807.30 | 3.81 | 3.02 | 3.41 | 0.56 |
| *pyrH* | 193.50 | 167.02 | 629.56 | 596.04 | 3.25 | 3.57 | 3.41 | 0.22 |
| *glyS* | 1004.77 | 925.65 | 3161.53 | 3395.87 | 3.15 | 3.67 | 3.41 | 0.37 |
| *SAUSA300_2295* | 42.07 | 38.68 | 129.21 | 142.90 | 3.07 | 3.69 | 3.38 | 0.44 |
| *SAUSA300_1450* | 241.58 | 195.15 | 680.88 | 763.38 | 2.82 | 3.91 | 3.37 | 0.77 |
| *SAUSA300_1216* | 93.75 | 107.24 | 326.23 | 344.63 | 3.48 | 3.21 | 3.35 | 0.19 |
| *SAUSA300_2627* | 584.11 | 509.85 | 1778.71 | 1851.53 | 3.05 | 3.63 | 3.34 | 0.41 |
| *gltS* | 69.71 | 44.83 | 174.11 | 187.22 | 2.50 | 4.18 | 3.34 | 1.19 |
| *rpsB* | 3848.40 | 3845.87 | 13181.30 | 12477.77 | 3.43 | 3.24 | 3.33 | 0.13 |
| *SAUSA300_1088* | 64.90 | 58.90 | 205.27 | 202.50 | 3.16 | 3.44 | 3.30 | 0.19 |
| *SAUSA300_1309* | 22.84 | 25.49 | 73.31 | 85.58 | 3.21 | 3.36 | 3.28 | 0.10 |
| *ureG* | 25.24 | 43.95 | 96.22 | 119.97 | 3.81 | 2.73 | 3.27 | 0.77 |
| *SAUSA300_1299* | 221.14 | 225.92 | 801.84 | 650.29 | 3.63 | 2.88 | 3.25 | 0.53 |
| *SAUSA300_0876* | 12.02 | 12.31 | 46.74 | 32.09 | 3.89 | 2.61 | 3.25 | 0.91 |
| *SAUSA300_1180* | 15.62 | 18.46 | 54.07 | 55.78 | 3.46 | 3.02 | 3.24 | 0.31 |
| *gatA* | 727.13 | 734.01 | 2372.52 | 2362.75 | 3.26 | 3.22 | 3.24 | 0.03 |
| *splF* | 76.92 | 69.45 | 224.51 | 246.06 | 2.92 | 3.54 | 3.23 | 0.44 |
| *SAUSA300_0859* | 97.35 | 96.70 | 336.31 | 289.61 | 3.45 | 3.00 | 3.22 | 0.32 |
| *SAUSA300_1890* | 415.85 | 447.44 | 1413.07 | 1353.31 | 3.40 | 3.02 | 3.21 | 0.26 |
| *SAUSA300_0349* | 8.41 | 7.03 | 22.91 | 25.98 | 2.72 | 3.69 | 3.21 | 0.69 |
| *opp-3C* | 48.07 | 25.49 | 109.05 | 104.69 | 2.27 | 4.11 | 3.19 | 1.30 |
| *cysM* | 20.43 | 19.34 | 62.31 | 64.19 | 3.05 | 3.32 | 3.18 | 0.19 |
| *tagG* | 693.48 | 608.31 | 2020.63 | 2095.29 | 2.91 | 3.44 | 3.18 | 0.38 |
| *SAUSA300_0640* | 629.78 | 654.02 | 2061.87 | 2013.53 | 3.27 | 3.08 | 3.18 | 0.14 |
| *SAUSA300_0479* | 5170.47 | 5208.41 | 16843.18 | 16115.88 | 3.26 | 3.09 | 3.18 | 0.12 |
| *rbsD* | 102.16 | 105.49 | 313.40 | 345.39 | 3.07 | 3.27 | 3.17 | 0.15 |
| *SAUSA300_1658* | 103.36 | 83.51 | 293.24 | 291.90 | 2.84 | 3.50 | 3.17 | 0.47 |
| *SAUSA300_0529* | 1805.22 | 2023.59 | 6137.04 | 5912.98 | 3.40 | 2.92 | 3.16 | 0.34 |
| *tagH* | 441.09 | 509.85 | 1556.94 | 1419.79 | 3.53 | 2.78 | 3.16 | 0.53 |
| *SAUSA300_2460* | 332.92 | 369.20 | 1200.47 | 993.39 | 3.61 | 2.69 | 3.15 | 0.65 |
| *udk* | 377.39 | 370.96 | 1227.04 | 1124.83 | 3.25 | 3.03 | 3.14 | 0.16 |
| *SAUSA300_0793* | 24.04 | 45.71 | 99.89 | 97.05 | 4.16 | 2.12 | 3.14 | 1.44 |
| *SAUSA300_1017* | 629.78 | 461.50 | 1619.25 | 1708.63 | 2.57 | 3.70 | 3.14 | 0.80 |
| *mqo* | 14975.36 | 14638.93 | 46327.00 | 46430.39 | 3.09 | 3.17 | 3.13 | 0.06 |
| *clpP* | 475.94 | 527.43 | 1638.50 | 1463.34 | 3.44 | 2.77 | 3.11 | 0.47 |
| *leuS* | 542.05 | 608.31 | 1757.63 | 1804.15 | 3.24 | 2.97 | 3.10 | 0.20 |
| *SAUSA300_0234* | 973.52 | 995.97 | 3208.27 | 2897.65 | 3.30 | 2.91 | 3.10 | 0.27 |
| *SAUSA300_2294* | 45.67 | 44.83 | 136.54 | 143.66 | 2.99 | 3.20 | 3.10 | 0.15 |
| *pgi* | 2373.70 | 2246.87 | 7206.46 | 7001.12 | 3.04 | 3.12 | 3.08 | 0.06 |
| *SAUSA300_1298* | 127.40 | 113.40 | 380.30 | 357.62 | 2.99 | 3.15 | 3.07 | 0.12 |
| *SAUSA300_0073* | 48.07 | 51.86 | 153.95 | 151.30 | 3.20 | 2.92 | 3.06 | 0.20 |
| *gid* | 376.19 | 377.99 | 1150.06 | 1153.86 | 3.06 | 3.05 | 3.05 | 0.00 |
| *SAUSA300_1297* | 40.86 | 49.23 | 132.88 | 140.60 | 3.25 | 2.86 | 3.05 | 0.28 |
| *sucC* | 1241.54 | 1256.17 | 3648.13 | 3978.15 | 2.94 | 3.17 | 3.05 | 0.16 |
| *tgt* | 508.39 | 652.26 | 1721.89 | 1760.60 | 3.39 | 2.70 | 3.04 | 0.49 |
| *SAUSA300_0696* | 133.41 | 131.86 | 418.79 | 386.66 | 3.14 | 2.93 | 3.04 | 0.15 |
| *groES* | 111.77 | 86.15 | 276.75 | 307.95 | 2.48 | 3.57 | 3.03 | 0.78 |
| *dltB* | 926.65 | 1097.06 | 2985.59 | 3021.44 | 3.22 | 2.75 | 2.99 | 0.33 |
| *rplK* | 5151.24 | 5234.78 | 15431.95 | 15539.71 | 3.00 | 2.97 | 2.98 | 0.02 |
| *SAUSA300_2485* | 330.52 | 385.91 | 1063.01 | 1059.87 | 3.22 | 2.75 | 2.98 | 0.33 |
| *SAUSA300_0930* | 63.70 | 72.96 | 197.94 | 207.85 | 3.11 | 2.85 | 2.98 | 0.18 |
| *sspB* | 37.26 | 33.40 | 86.14 | 121.50 | 2.31 | 3.64 | 2.97 | 0.94 |
| *greA* | 526.42 | 465.90 | 1480.88 | 1460.29 | 2.81 | 3.13 | 2.97 | 0.23 |
| *ackA* | 2880.89 | 2545.75 | 7669.24 | 8346.79 | 2.66 | 3.28 | 2.97 | 0.44 |
| *SAUSA300_0920* | 32.45 | 22.86 | 91.64 | 71.07 | 2.82 | 3.11 | 2.97 | 0.20 |
| *SAUSA300_0256* | 729.54 | 799.06 | 2370.69 | 2138.09 | 3.25 | 2.68 | 2.96 | 0.41 |
| *SAUSA300_2560* | 85.33 | 96.70 | 276.75 | 258.28 | 3.24 | 2.67 | 2.96 | 0.40 |
| *SAUSA300_0933* | 493.97 | 507.22 | 1171.14 | 1784.29 | 2.37 | 3.52 | 2.94 | 0.81 |
| *menD* | 277.63 | 270.75 | 811.92 | 801.59 | 2.92 | 2.96 | 2.94 | 0.03 |
| *dltA* | 1156.20 | 1195.52 | 3364.05 | 3547.17 | 2.91 | 2.97 | 2.94 | 0.04 |
| *SAUSA300_1720* | 282.44 | 291.85 | 769.76 | 914.69 | 2.73 | 3.13 | 2.93 | 0.29 |
| *rpsL* | 5891.59 | 6126.15 | 17602.87 | 17502.04 | 2.99 | 2.86 | 2.92 | 0.09 |
| *topA* | 436.28 | 411.40 | 1237.12 | 1235.63 | 2.84 | 3.00 | 2.92 | 0.12 |
| *asnC* | 861.74 | 907.19 | 2636.44 | 2517.10 | 3.06 | 2.77 | 2.92 | 0.20 |
| *SAUSA300_0695* | 496.37 | 505.46 | 1505.62 | 1406.80 | 3.03 | 2.78 | 2.91 | 0.18 |
| *SAUSA300_1254* | 530.03 | 520.40 | 1451.56 | 1596.30 | 2.74 | 3.07 | 2.90 | 0.23 |
| *SAUSA300_1463* | 64.90 | 77.36 | 169.53 | 246.82 | 2.61 | 3.19 | 2.90 | 0.41 |
| *SAUSA300_2537* | 4273.87 | 4039.27 | 11272.47 | 12736.82 | 2.64 | 3.15 | 2.90 | 0.36 |
| *SAUSA300_2445* | 277.63 | 294.48 | 866.90 | 784.02 | 3.12 | 2.66 | 2.89 | 0.33 |
| *rplS* | 3668.12 | 3716.65 | 10650.24 | 10665.98 | 2.90 | 2.87 | 2.89 | 0.02 |
| *SAUSA300_2504* | 592.52 | 571.39 | 1603.68 | 1746.84 | 2.71 | 3.06 | 2.88 | 0.25 |
| *entB* | 61.30 | 60.65 | 182.36 | 168.88 | 2.98 | 2.78 | 2.88 | 0.13 |
| *gatC* | 85.33 | 68.57 | 255.67 | 187.98 | 3.00 | 2.74 | 2.87 | 0.18 |
| *SAUSA300_1369* | 387.00 | 398.21 | 1049.26 | 1205.06 | 2.71 | 3.03 | 2.87 | 0.22 |
| *sak* | 1159.81 | 1219.25 | 3483.18 | 3329.39 | 3.00 | 2.73 | 2.87 | 0.19 |
| *kdpE* | 18.03 | 17.58 | 53.15 | 48.91 | 2.95 | 2.78 | 2.86 | 0.12 |
| *SAUSA300_1495* | 379.79 | 394.70 | 1175.72 | 1034.66 | 3.10 | 2.62 | 2.86 | 0.34 |
| *SAUSA300_2083* | 99.76 | 101.97 | 300.57 | 275.09 | 3.01 | 2.70 | 2.86 | 0.22 |
| *tdk* | 157.45 | 176.69 | 449.95 | 497.46 | 2.86 | 2.82 | 2.84 | 0.03 |
| *SAUSA300_0077* | 10.82 | 13.19 | 29.32 | 38.97 | 2.71 | 2.96 | 2.83 | 0.17 |
| *SAUSA300_0093* | 32.45 | 32.53 | 99.89 | 84.06 | 3.08 | 2.58 | 2.83 | 0.35 |
| *vraR* | 247.59 | 281.30 | 767.02 | 711.42 | 3.10 | 2.53 | 2.81 | 0.40 |
| *engB* | 32.45 | 38.68 | 114.55 | 81.00 | 3.53 | 2.09 | 2.81 | 1.02 |
| *ureF* | 21.63 | 29.01 | 74.23 | 63.42 | 3.43 | 2.19 | 2.81 | 0.88 |
| *pheS* | 167.06 | 189.00 | 512.26 | 476.06 | 3.07 | 2.52 | 2.79 | 0.39 |
| *SAUSA300_2516* | 10.82 | 22.86 | 32.07 | 59.60 | 2.97 | 2.61 | 2.79 | 0.25 |
| *lpdA* | 15248.19 | 14342.69 | 41776.22 | 40361.53 | 2.74 | 2.81 | 2.78 | 0.05 |
| *cyoE* | 903.81 | 940.59 | 2448.58 | 2662.29 | 2.71 | 2.83 | 2.77 | 0.09 |
| *SAUSA300_0291* | 16.83 | 8.79 | 37.57 | 29.04 | 2.23 | 3.30 | 2.77 | 0.76 |
| *SAUSA300_1432* | 10.82 | 16.70 | 37.57 | 34.39 | 3.47 | 2.06 | 2.77 | 1.00 |
| *SAUSA300_1993* | 262.01 | 263.72 | 727.61 | 725.94 | 2.78 | 2.75 | 2.76 | 0.02 |
| *SAUSA300_0327* | 19.23 | 29.89 | 65.98 | 62.66 | 3.43 | 2.10 | 2.76 | 0.94 |
| *rplJ* | 6300.23 | 6042.64 | 16748.79 | 17225.42 | 2.66 | 2.85 | 2.75 | 0.14 |
| *menB* | 1510.76 | 1348.47 | 4036.68 | 3759.61 | 2.67 | 2.79 | 2.73 | 0.08 |
| *sbi* | 1210.29 | 1203.43 | 3472.19 | 3108.55 | 2.87 | 2.58 | 2.73 | 0.20 |
| *SAUSA300_0518* | 169.46 | 196.03 | 478.35 | 514.27 | 2.82 | 2.62 | 2.72 | 0.14 |
| *rpmG* | 1249.95 | 1339.68 | 4082.50 | 2853.33 | 3.27 | 2.13 | 2.70 | 0.80 |
| *SAUSA300_0292* | 57.69 | 48.35 | 159.45 | 126.85 | 2.76 | 2.62 | 2.69 | 0.10 |
| *murC* | 742.76 | 854.44 | 2116.85 | 2154.13 | 2.85 | 2.52 | 2.69 | 0.23 |
| *SAUSA300_0007* | 558.87 | 666.33 | 1631.17 | 1628.40 | 2.92 | 2.44 | 2.68 | 0.34 |
| *mreC* | 217.54 | 250.53 | 620.39 | 628.13 | 2.85 | 2.51 | 2.68 | 0.24 |
| *tsf* | 2543.17 | 2417.41 | 6799.59 | 6483.03 | 2.67 | 2.68 | 2.68 | 0.01 |
| *SAUSA300_2473* | 399.02 | 419.31 | 1051.10 | 1134.76 | 2.63 | 2.71 | 2.67 | 0.05 |
| *SAUSA300_1733* | 24.04 | 39.56 | 72.39 | 91.70 | 3.01 | 2.32 | 2.66 | 0.49 |
| *eutD* | 2718.64 | 2922.86 | 7624.34 | 7354.16 | 2.80 | 2.52 | 2.66 | 0.20 |
| *fmt* | 306.48 | 273.39 | 811.00 | 718.30 | 2.65 | 2.63 | 2.64 | 0.01 |
| *tcaA* | 228.36 | 186.36 | 562.66 | 521.91 | 2.46 | 2.80 | 2.63 | 0.24 |
| *SAUSA300_2519* | 45.67 | 27.25 | 86.14 | 91.70 | 1.89 | 3.36 | 2.63 | 1.05 |
| *SAUSA300_1569* | 462.72 | 489.63 | 1207.80 | 1289.12 | 2.61 | 2.63 | 2.62 | 0.02 |
| *SAUSA300_0233* | 56.49 | 47.47 | 154.87 | 118.44 | 2.74 | 2.50 | 2.62 | 0.17 |
| *pdhA* | 9268.86 | 8850.34 | 23464.07 | 23855.17 | 2.53 | 2.70 | 2.61 | 0.12 |
| *SAUSA300_1875* | 72.11 | 63.29 | 198.86 | 155.89 | 2.76 | 2.46 | 2.61 | 0.21 |
| *hemE* | 240.37 | 237.35 | 582.82 | 660.22 | 2.42 | 2.78 | 2.60 | 0.25 |
| *panB* | 249.99 | 228.55 | 566.33 | 670.16 | 2.27 | 2.93 | 2.60 | 0.47 |
| *lspA* | 56.49 | 75.60 | 176.86 | 154.36 | 3.13 | 2.04 | 2.59 | 0.77 |
| *SAUSA300_1020* | 123.79 | 133.62 | 343.64 | 320.18 | 2.78 | 2.40 | 2.59 | 0.27 |
| *nsaS* | 319.70 | 312.94 | 841.24 | 794.71 | 2.63 | 2.54 | 2.59 | 0.06 |
| *SAUSA300_2147* | 326.91 | 276.02 | 817.42 | 735.87 | 2.50 | 2.67 | 2.58 | 0.12 |
| *SAUSA300_1006* | 70.91 | 59.78 | 174.11 | 162.00 | 2.46 | 2.71 | 2.58 | 0.18 |
| *ileS* | 978.33 | 1054.87 | 2582.38 | 2622.56 | 2.64 | 2.49 | 2.56 | 0.11 |
| *argH* | 66.10 | 101.09 | 219.93 | 181.10 | 3.33 | 1.79 | 2.56 | 1.09 |
| *SAUSA300_1339* | 331.72 | 363.05 | 936.55 | 829.86 | 2.82 | 2.29 | 2.55 | 0.38 |
| *femB* | 723.53 | 779.72 | 1961.07 | 1858.41 | 2.71 | 2.38 | 2.55 | 0.23 |
| *ansA* | 85.33 | 95.82 | 245.59 | 211.67 | 2.88 | 2.21 | 2.54 | 0.47 |
| *gpmA* | 5930.05 | 5736.73 | 14968.26 | 14532.56 | 2.52 | 2.53 | 2.53 | 0.01 |
| *glmS* | 1931.41 | 1735.26 | 4838.52 | 4417.54 | 2.51 | 2.55 | 2.53 | 0.03 |
| *SAUSA300_1703* | 40.86 | 78.24 | 136.54 | 133.73 | 3.34 | 1.71 | 2.53 | 1.15 |
| *rpoE* | 503.59 | 549.41 | 1369.08 | 1278.42 | 2.72 | 2.33 | 2.52 | 0.28 |
| *SAUSA300_0769* | 69.71 | 70.32 | 186.94 | 165.82 | 2.68 | 2.36 | 2.52 | 0.23 |
| *SAUSA300_2406* | 194.70 | 220.64 | 498.51 | 544.84 | 2.56 | 2.47 | 2.51 | 0.06 |
| *SAUSA300_1915* | 16.83 | 36.92 | 52.23 | 71.07 | 3.10 | 1.92 | 2.51 | 0.83 |
| *SAUSA300_0248* | 44.47 | 32.53 | 104.47 | 87.11 | 2.35 | 2.68 | 2.51 | 0.23 |
| *ureE* | 25.24 | 14.06 | 51.32 | 42.03 | 2.03 | 2.99 | 2.51 | 0.68 |
| *SAUSA300_0941* | 14.42 | 22.86 | 36.66 | 56.55 | 2.54 | 2.47 | 2.51 | 0.05 |
| *SAUSA300_0289* | 302.87 | 345.47 | 876.07 | 731.29 | 2.89 | 2.12 | 2.50 | 0.55 |
| *SAUSA300_0940* | 21.63 | 12.31 | 55.90 | 29.80 | 2.58 | 2.42 | 2.50 | 0.11 |
| *vraS* | 204.32 | 239.98 | 569.08 | 530.32 | 2.79 | 2.21 | 2.50 | 0.41 |
| *rplC* | 3140.50 | 3293.83 | 8051.37 | 7997.57 | 2.56 | 2.43 | 2.50 | 0.10 |
| *rho* | 1097.31 | 1154.20 | 2816.97 | 2785.32 | 2.57 | 2.41 | 2.49 | 0.11 |
| *SAUSA300_1613* | 280.04 | 296.24 | 680.88 | 742.75 | 2.43 | 2.51 | 2.47 | 0.05 |
| *tkt* | 2674.17 | 2936.05 | 7051.59 | 6726.03 | 2.64 | 2.29 | 2.46 | 0.24 |
| *panE* | 348.54 | 334.92 | 804.59 | 876.48 | 2.31 | 2.62 | 2.46 | 0.22 |
| *SAUSA300_0571* | 2273.95 | 2128.20 | 5444.25 | 5340.63 | 2.39 | 2.51 | 2.45 | 0.08 |
| *SAUSA300_1732* | 57.69 | 49.23 | 150.29 | 113.09 | 2.61 | 2.30 | 2.45 | 0.22 |
| *SAUSA300_0641* | 38.46 | 49.23 | 103.55 | 108.51 | 2.69 | 2.20 | 2.45 | 0.35 |
| *SAUSA300_0912* | 775.21 | 826.31 | 1965.65 | 1935.59 | 2.54 | 2.34 | 2.44 | 0.14 |
| *nusG* | 467.53 | 469.42 | 1133.57 | 1151.57 | 2.42 | 2.45 | 2.44 | 0.02 |
| *SAUSA300_2052* | 36.06 | 40.44 | 101.72 | 82.53 | 2.82 | 2.04 | 2.43 | 0.55 |
| *SAUSA300_0826* | 60.09 | 94.06 | 188.78 | 158.94 | 3.14 | 1.69 | 2.42 | 1.03 |
| *rbsK* | 379.79 | 333.16 | 832.08 | 879.53 | 2.19 | 2.64 | 2.42 | 0.32 |
| *SAUSA300_0995* | 10536.84 | 10255.96 | 25410.48 | 24736.23 | 2.41 | 2.41 | 2.41 | 0.00 |
| *SAUSA300_1247* | 164.66 | 181.09 | 419.70 | 411.88 | 2.55 | 2.27 | 2.41 | 0.19 |
| *pfkA* | 765.59 | 1155.96 | 2297.38 | 2099.88 | 3.00 | 1.82 | 2.41 | 0.84 |
| *SAUSA300_0918* | 189.90 | 196.03 | 450.86 | 478.36 | 2.37 | 2.44 | 2.41 | 0.05 |
| *SAUSA300_2481* | 5482.95 | 6330.97 | 14600.78 | 13551.40 | 2.66 | 2.14 | 2.40 | 0.37 |
| *SAUSA300_2040* | 254.80 | 280.42 | 599.32 | 686.97 | 2.35 | 2.45 | 2.40 | 0.07 |
| *SAUSA300_1721* | 46.87 | 70.32 | 129.21 | 143.66 | 2.76 | 2.04 | 2.40 | 0.50 |
| *mreD* | 139.42 | 145.04 | 340.90 | 340.81 | 2.45 | 2.35 | 2.40 | 0.07 |
| *panD* | 181.48 | 122.19 | 344.56 | 353.80 | 1.90 | 2.90 | 2.40 | 0.70 |
| *SAUSA300_0517* | 167.06 | 179.33 | 399.54 | 427.16 | 2.39 | 2.38 | 2.39 | 0.01 |
| *SAUSA300_2387* | 52.88 | 60.65 | 133.79 | 135.25 | 2.53 | 2.23 | 2.38 | 0.21 |
| *radA* | 493.97 | 561.72 | 1222.46 | 1280.71 | 2.47 | 2.28 | 2.38 | 0.14 |
| *SAUSA300_0650* | 979.53 | 942.35 | 2203.91 | 2358.16 | 2.25 | 2.50 | 2.38 | 0.18 |
| *gyrA* | 2819.60 | 3023.96 | 6641.97 | 7242.59 | 2.36 | 2.40 | 2.38 | 0.03 |
| *SAUSA300_1071* | 276.43 | 346.35 | 788.09 | 652.58 | 2.85 | 1.88 | 2.37 | 0.68 |
| *ampA* | 346.14 | 285.69 | 703.78 | 771.03 | 2.03 | 2.70 | 2.37 | 0.47 |
| *SAUSA300_1570* | 157.45 | 142.41 | 344.56 | 362.21 | 2.19 | 2.54 | 2.37 | 0.25 |
| *yajC* | 305.28 | 287.45 | 797.26 | 608.26 | 2.61 | 2.12 | 2.36 | 0.35 |
| *accC* | 504.79 | 508.09 | 1142.73 | 1250.91 | 2.26 | 2.46 | 2.36 | 0.14 |
| *SAUSA300_1888* | 272.83 | 259.32 | 649.72 | 605.21 | 2.38 | 2.33 | 2.36 | 0.03 |
| *SAUSA300_0531* | 6190.86 | 6110.32 | 14821.63 | 14138.26 | 2.39 | 2.31 | 2.35 | 0.06 |
| *SAUSA300_1346* | 651.42 | 709.40 | 1639.42 | 1546.64 | 2.52 | 2.18 | 2.35 | 0.24 |
| *rbfA* | 486.76 | 456.23 | 1152.81 | 1053.76 | 2.37 | 2.31 | 2.34 | 0.04 |
| *SAUSA300_0590* | 352.15 | 399.97 | 854.07 | 897.87 | 2.43 | 2.24 | 2.34 | 0.13 |
| *trxB* | 747.57 | 811.37 | 1823.61 | 1804.15 | 2.44 | 2.22 | 2.33 | 0.15 |
| *rpsO* | 4335.16 | 4474.40 | 10904.08 | 9596.93 | 2.52 | 2.14 | 2.33 | 0.26 |
| *SAUSA300_0489* | 2424.18 | 2486.85 | 5612.87 | 5820.51 | 2.32 | 2.34 | 2.33 | 0.02 |
| *ssb* | 4746.20 | 5720.90 | 12374.88 | 11718.97 | 2.61 | 2.05 | 2.33 | 0.40 |
| *SAUSA300_1534* | 1747.53 | 1710.64 | 4113.66 | 3915.49 | 2.35 | 2.29 | 2.32 | 0.05 |
| *SAUSA300_0942* | 44.47 | 50.11 | 109.97 | 108.51 | 2.47 | 2.17 | 2.32 | 0.22 |
| *SAUSA300_0090* | 14.42 | 30.77 | 42.15 | 52.73 | 2.92 | 1.71 | 2.32 | 0.85 |
| *pdhB* | 6855.49 | 6210.54 | 15086.47 | 14971.19 | 2.20 | 2.41 | 2.31 | 0.15 |
| *SAUSA300_0135* | 1671.81 | 1561.21 | 3769.10 | 3651.86 | 2.25 | 2.34 | 2.30 | 0.06 |
| *SAUSA300_2559* | 288.45 | 279.54 | 666.21 | 635.77 | 2.31 | 2.27 | 2.29 | 0.02 |
| *rplU* | 4391.65 | 4816.35 | 10862.84 | 10153.23 | 2.47 | 2.11 | 2.29 | 0.26 |
| *rplL* | 3425.34 | 3489.86 | 7940.49 | 7828.70 | 2.32 | 2.24 | 2.28 | 0.05 |
| *hlgB* | 169.46 | 115.16 | 311.57 | 313.30 | 1.84 | 2.72 | 2.28 | 0.62 |
| *SAUSA300_2552* | 40.86 | 33.40 | 94.39 | 74.89 | 2.31 | 2.24 | 2.28 | 0.05 |
| *pcrA* | 304.07 | 299.76 | 613.06 | 757.27 | 2.02 | 2.53 | 2.27 | 0.36 |
| *SAUSA300_0519* | 120.19 | 155.59 | 298.74 | 319.41 | 2.49 | 2.05 | 2.27 | 0.31 |
| *tcaB* | 72.11 | 69.45 | 153.04 | 167.35 | 2.12 | 2.41 | 2.27 | 0.20 |
| *dnaG* | 710.31 | 745.44 | 1634.83 | 1637.57 | 2.30 | 2.20 | 2.25 | 0.07 |
| *SAUSA300_1918* | 90.14 | 94.06 | 220.85 | 192.57 | 2.45 | 2.05 | 2.25 | 0.28 |
| *rpsR* | 1456.67 | 1645.59 | 3603.23 | 3323.28 | 2.47 | 2.02 | 2.25 | 0.32 |
| *ureD* | 79.32 | 56.26 | 142.96 | 151.30 | 1.80 | 2.69 | 2.25 | 0.63 |
| *fmtC* | 1162.21 | 1354.63 | 2707.01 | 2912.17 | 2.33 | 2.15 | 2.24 | 0.13 |
| *SAUSA300_1797* | 70.91 | 81.75 | 152.12 | 190.27 | 2.15 | 2.33 | 2.24 | 0.13 |
| *SAUSA300_0031* | 48.07 | 23.73 | 78.81 | 67.25 | 1.64 | 2.83 | 2.24 | 0.84 |
| *coaD* | 82.93 | 94.94 | 185.11 | 212.43 | 2.23 | 2.24 | 2.23 | 0.00 |
| *SAUSA300_0261* | 28.84 | 43.95 | 69.65 | 90.17 | 2.41 | 2.05 | 2.23 | 0.26 |
| *ptsG* | 1026.40 | 991.58 | 2239.65 | 2262.64 | 2.18 | 2.28 | 2.23 | 0.07 |
| *SAUSA300_0628* | 60.09 | 81.75 | 157.62 | 149.77 | 2.62 | 1.83 | 2.23 | 0.56 |
| *SAUSA300_0557* | 21.63 | 20.22 | 46.74 | 45.85 | 2.16 | 2.27 | 2.21 | 0.08 |
| *SAUSA300_1101* | 383.40 | 403.49 | 922.80 | 803.88 | 2.41 | 1.99 | 2.20 | 0.29 |
| *SAUSA300_1171* | 62.50 | 43.95 | 87.97 | 131.43 | 1.41 | 2.99 | 2.20 | 1.12 |
| *SAUSA300_0638* | 40.86 | 37.80 | 80.64 | 90.93 | 1.97 | 2.41 | 2.19 | 0.31 |
| *SAUSA300_1285* | 411.04 | 334.04 | 746.85 | 853.55 | 1.82 | 2.56 | 2.19 | 0.52 |
| *rex* | 451.90 | 487.00 | 1005.28 | 1042.30 | 2.22 | 2.14 | 2.18 | 0.06 |
| *SAUSA300_1533* | 2157.37 | 2098.31 | 4682.73 | 4581.83 | 2.17 | 2.18 | 2.18 | 0.01 |
| *SAUSA300_0516* | 56.49 | 93.18 | 158.53 | 143.66 | 2.81 | 1.54 | 2.17 | 0.89 |
| *sucD* | 2129.72 | 1863.60 | 4216.29 | 4397.67 | 1.98 | 2.36 | 2.17 | 0.27 |
| *pyk* | 8470.81 | 9086.81 | 19580.43 | 18426.66 | 2.31 | 2.03 | 2.17 | 0.20 |
| *nupC* | 1348.50 | 1296.61 | 2763.82 | 2941.21 | 2.05 | 2.27 | 2.16 | 0.15 |
| *SAUSA300_1325* | 21.63 | 29.89 | 51.32 | 58.08 | 2.37 | 1.94 | 2.16 | 0.30 |
| *pbp3* | 413.44 | 388.54 | 835.74 | 889.47 | 2.02 | 2.29 | 2.16 | 0.19 |
| *rpsF* | 3969.79 | 4688.01 | 9468.10 | 8911.49 | 2.39 | 1.90 | 2.14 | 0.34 |
| *ribH* | 19.23 | 27.25 | 40.32 | 59.60 | 2.10 | 2.19 | 2.14 | 0.06 |
| *SAUSA300_1158* | 215.14 | 253.17 | 488.43 | 501.28 | 2.27 | 1.98 | 2.13 | 0.21 |
| *SAUSA300_0732* | 102.16 | 99.33 | 197.94 | 229.24 | 1.94 | 2.31 | 2.12 | 0.26 |
| *SAUSA300_1374* | 325.71 | 321.73 | 685.46 | 683.91 | 2.10 | 2.13 | 2.12 | 0.01 |
| *SAUSA300_0671* | 307.68 | 366.57 | 767.02 | 635.77 | 2.49 | 1.73 | 2.11 | 0.54 |
| *hlgC* | 70.91 | 86.15 | 169.53 | 158.18 | 2.39 | 1.84 | 2.11 | 0.39 |
| *cinA* | 60.09 | 64.17 | 130.13 | 132.20 | 2.17 | 2.06 | 2.11 | 0.07 |
| *murE* | 176.68 | 203.06 | 394.96 | 400.41 | 2.24 | 1.97 | 2.10 | 0.19 |
| *SAUSA300_2483* | 582.91 | 571.39 | 1184.89 | 1236.39 | 2.03 | 2.16 | 2.10 | 0.09 |
| *SAUSA300_1004* | 78.12 | 102.85 | 154.87 | 226.95 | 1.98 | 2.21 | 2.09 | 0.16 |
| *SAUSA300_0620* | 9270.06 | 10692.85 | 19867.26 | 21807.25 | 2.14 | 2.04 | 2.09 | 0.07 |
| *SAUSA300_1876* | 85.33 | 85.27 | 188.78 | 167.35 | 2.21 | 1.96 | 2.09 | 0.18 |
| *cdsA* | 219.94 | 225.04 | 449.03 | 475.30 | 2.04 | 2.11 | 2.08 | 0.05 |
| *rpsI* | 2569.61 | 2590.58 | 5398.43 | 5305.48 | 2.10 | 2.05 | 2.07 | 0.04 |
| *SAUSA300_2212* | 66.10 | 47.47 | 114.55 | 114.62 | 1.73 | 2.41 | 2.07 | 0.48 |
| *SAUSA300_2377* | 391.81 | 393.82 | 815.58 | 810.76 | 2.08 | 2.06 | 2.07 | 0.02 |
| *SAUSA300_2085* | 701.89 | 729.62 | 1320.51 | 1647.50 | 1.88 | 2.26 | 2.07 | 0.27 |
| *ctsR* | 271.62 | 243.50 | 553.50 | 508.16 | 2.04 | 2.09 | 2.06 | 0.03 |
| *ung* | 174.27 | 141.53 | 287.75 | 349.98 | 1.65 | 2.47 | 2.06 | 0.58 |
| *SAUSA300_0341* | 128.60 | 190.76 | 298.74 | 342.34 | 2.32 | 1.79 | 2.06 | 0.37 |
| *folD* | 1411.00 | 1318.59 | 2739.08 | 2861.73 | 1.94 | 2.17 | 2.06 | 0.16 |
| *recX* | 22.84 | 29.01 | 60.48 | 42.03 | 2.65 | 1.45 | 2.05 | 0.85 |
| *SAUSA300_0575* | 99.76 | 101.09 | 211.69 | 197.91 | 2.12 | 1.96 | 2.04 | 0.12 |
| *cysE* | 81.73 | 88.78 | 180.53 | 165.82 | 2.21 | 1.87 | 2.04 | 0.24 |
| *SAUSA300_1223* | 1656.18 | 1433.74 | 3084.56 | 3158.99 | 1.86 | 2.20 | 2.03 | 0.24 |
| *SAUSA300_0039* | 84.13 | 80.87 | 152.12 | 181.87 | 1.81 | 2.25 | 2.03 | 0.31 |
| *SAUSA300_1470* | 469.93 | 625.01 | 1144.57 | 1011.73 | 2.44 | 1.62 | 2.03 | 0.58 |
| *prfC* | 612.96 | 551.17 | 1154.65 | 1195.13 | 1.88 | 2.17 | 2.03 | 0.20 |
| *SAUSA300_1602* | 2096.07 | 2193.25 | 4473.80 | 4201.29 | 2.13 | 1.92 | 2.02 | 0.15 |
| *SAUSA300_1706* | 170.67 | 166.14 | 309.74 | 369.85 | 1.81 | 2.23 | 2.02 | 0.29 |
| *SAUSA300_1687* | 1402.59 | 1384.51 | 2652.02 | 2975.59 | 1.89 | 2.15 | 2.02 | 0.18 |
| *SAUSA300_0649* | 679.06 | 678.63 | 1253.62 | 1484.74 | 1.85 | 2.19 | 2.02 | 0.24 |
| *SAUSA300_1988* | 47149.55 | 48847.43 | 98841.44 | 94389.08 | 2.10 | 1.93 | 2.01 | 0.12 |
| *SAUSA300_0958* | 709.11 | 671.60 | 1382.83 | 1392.28 | 1.95 | 2.07 | 2.01 | 0.09 |
| *trx* | 431.47 | 479.09 | 1033.68 | 777.90 | 2.40 | 1.62 | 2.01 | 0.55 |
| *SAUSA300_1373* | 133.41 | 181.96 | 299.66 | 320.94 | 2.25 | 1.76 | 2.00 | 0.34 |
| *pnbA* | 1634.55 | 1432.86 | 3188.11 | 2941.97 | 1.95 | 2.05 | 2.00 | 0.07 |
| *SAUSA300_0858* | 998.76 | 1108.49 | 2125.10 | 2076.19 | 2.13 | 1.87 | 2.00 | 0.18 |
| *SAUSA300_0670* | 252.39 | 239.10 | 489.35 | 492.88 | 1.94 | 2.06 | 2.00 | 0.09 |
| *SAUSA300_2004* | 51.68 | 41.32 | 86.14 | 96.28 | 1.67 | 2.33 | 2.00 | 0.47 |
| *SAUSA300_0081* | 88.94 | 100.21 | 189.69 | 186.45 | 2.13 | 1.86 | 2.00 | 0.19 |
| *panC* | 365.37 | 434.25 | 764.27 | 824.52 | 2.09 | 1.90 | 2.00 | 0.14 |
| *secF* | 3019.11 | 3124.17 | 6124.21 | 6118.53 | 2.03 | 1.96 | 1.99 | 0.05 |
| *SAUSA300_2325* | 169.46 | 164.38 | 367.47 | 298.78 | 2.17 | 1.82 | 1.99 | 0.25 |
| *SAUSA300_1086* | 624.97 | 694.45 | 1278.36 | 1337.26 | 2.05 | 1.93 | 1.99 | 0.08 |
| *SAUSA300_1532* | 2428.99 | 2283.79 | 4921.91 | 4435.11 | 2.03 | 1.94 | 1.98 | 0.06 |
| *SAUSA300_2408* | 36.06 | 46.59 | 74.23 | 87.88 | 2.06 | 1.89 | 1.97 | 0.12 |
| *htrA* | 84.13 | 116.91 | 213.52 | 163.53 | 2.54 | 1.40 | 1.97 | 0.81 |
| *SAUSA300_1119* | 795.64 | 738.41 | 1385.58 | 1616.94 | 1.74 | 2.19 | 1.97 | 0.32 |
| *SAUSA300_1585* | 485.56 | 461.50 | 946.63 | 909.34 | 1.95 | 1.97 | 1.96 | 0.01 |
| *SAUSA300_2378* | 582.91 | 521.28 | 1145.48 | 1018.61 | 1.97 | 1.95 | 1.96 | 0.01 |
| *rpmA* | 2889.31 | 3125.93 | 5988.59 | 5724.23 | 2.07 | 1.83 | 1.95 | 0.17 |
| *polC* | 1421.82 | 1261.45 | 2686.84 | 2507.17 | 1.89 | 1.99 | 1.94 | 0.07 |
| *SAUSA300_1019* | 110.57 | 174.93 | 260.25 | 265.16 | 2.35 | 1.52 | 1.93 | 0.59 |
| *prfA* | 519.21 | 466.78 | 968.62 | 933.02 | 1.87 | 2.00 | 1.93 | 0.09 |
| *SAUSA300_2528* | 66.10 | 78.24 | 132.88 | 144.42 | 2.01 | 1.85 | 1.93 | 0.12 |
| *SAUSA300_1692* | 395.42 | 397.33 | 834.83 | 693.08 | 2.11 | 1.74 | 1.93 | 0.26 |
| *SAUSA300_1550* | 316.09 | 383.27 | 642.39 | 698.43 | 2.03 | 1.82 | 1.93 | 0.15 |
| *SAUSA300_1571* | 39.66 | 44.83 | 83.39 | 77.94 | 2.10 | 1.74 | 1.92 | 0.26 |
| *rpsU* | 3059.97 | 3125.93 | 6104.05 | 5764.73 | 1.99 | 1.84 | 1.92 | 0.11 |
| *SAUSA300_1662* | 44.47 | 64.17 | 97.14 | 105.45 | 2.18 | 1.64 | 1.91 | 0.38 |
| *SAUSA300_1025* | 86.53 | 94.06 | 165.87 | 179.57 | 1.92 | 1.91 | 1.91 | 0.01 |
| *SAUSA300_1197* | 122.59 | 145.04 | 261.17 | 240.71 | 2.13 | 1.66 | 1.89 | 0.33 |
| *SAUSA300_2264* | 1240.33 | 1159.48 | 2312.04 | 2212.21 | 1.86 | 1.91 | 1.89 | 0.03 |
| *dps* | 1511.96 | 1567.36 | 3265.08 | 2503.35 | 2.16 | 1.60 | 1.88 | 0.40 |
| *SAUSA300_1895* | 2096.07 | 2130.83 | 4051.34 | 3881.87 | 1.93 | 1.82 | 1.88 | 0.08 |
| *rplD* | 3271.50 | 3216.47 | 6084.81 | 6078.03 | 1.86 | 1.89 | 1.87 | 0.02 |
| *secE* | 175.47 | 191.63 | 352.81 | 333.17 | 2.01 | 1.74 | 1.87 | 0.19 |
| *SAUSA300_2094* | 205.52 | 236.47 | 392.21 | 434.80 | 1.91 | 1.84 | 1.87 | 0.05 |
| *tagX* | 145.43 | 110.76 | 222.68 | 245.29 | 1.53 | 2.21 | 1.87 | 0.48 |
| *SAUSA300_0957* | 403.83 | 423.71 | 784.43 | 758.80 | 1.94 | 1.79 | 1.87 | 0.11 |
| *ribF* | 249.99 | 254.93 | 457.28 | 484.47 | 1.83 | 1.90 | 1.86 | 0.05 |
| *SAUSA300_2069* | 102.16 | 107.24 | 198.86 | 191.04 | 1.95 | 1.78 | 1.86 | 0.12 |
| *hup* | 13361.24 | 13643.84 | 26158.25 | 24114.98 | 1.96 | 1.77 | 1.86 | 0.13 |
| *SAUSA300_1884* | 624.97 | 705.00 | 1289.36 | 1169.91 | 2.06 | 1.66 | 1.86 | 0.29 |
| *SAUSA300_2433* | 194.70 | 168.78 | 327.15 | 343.10 | 1.68 | 2.03 | 1.86 | 0.25 |
| *SAUSA300_2480* | 81.73 | 117.79 | 186.94 | 167.35 | 2.29 | 1.42 | 1.85 | 0.61 |
| *rplB* | 7468.45 | 6980.59 | 13558.85 | 13202.95 | 1.82 | 1.89 | 1.85 | 0.05 |
| *rnc* | 39.66 | 43.07 | 80.64 | 71.83 | 2.03 | 1.67 | 1.85 | 0.26 |
| *SAUSA300_2284* | 69.71 | 80.87 | 132.88 | 144.42 | 1.91 | 1.79 | 1.85 | 0.09 |
| *SAUSA300_0830* | 94.95 | 107.24 | 175.03 | 197.91 | 1.84 | 1.85 | 1.84 | 0.00 |
| *SAUSA300_2580* | 272.83 | 286.57 | 508.59 | 522.68 | 1.86 | 1.82 | 1.84 | 0.03 |
| *SAUSA300_2071* | 725.93 | 693.58 | 1271.94 | 1341.08 | 1.75 | 1.93 | 1.84 | 0.13 |
| *SAUSA300_1069* | 48.07 | 58.90 | 98.97 | 95.52 | 2.06 | 1.62 | 1.84 | 0.31 |
| *cysK* | 6730.50 | 7155.52 | 12660.79 | 12843.03 | 1.88 | 1.79 | 1.84 | 0.06 |
| *fba* | 7437.20 | 7028.94 | 13699.98 | 12818.58 | 1.84 | 1.82 | 1.83 | 0.01 |
| *SAUSA300_2562* | 197.11 | 204.82 | 404.13 | 330.88 | 2.05 | 1.62 | 1.83 | 0.31 |
| *uppS* | 323.30 | 317.34 | 591.99 | 577.70 | 1.83 | 1.82 | 1.83 | 0.01 |
| *rplW* | 1294.42 | 1225.41 | 2329.45 | 2258.06 | 1.80 | 1.84 | 1.82 | 0.03 |
| *SAUSA300_2332* | 194.70 | 170.54 | 314.32 | 343.10 | 1.61 | 2.01 | 1.81 | 0.28 |
| *SAUSA300_2163* | 74.52 | 81.75 | 143.87 | 136.78 | 1.93 | 1.67 | 1.80 | 0.18 |
| *SAUSA300_2286* | 92.54 | 109.00 | 208.94 | 146.72 | 2.26 | 1.35 | 1.80 | 0.64 |
| *SAUSA300_1979* | 134.61 | 126.58 | 183.28 | 283.50 | 1.36 | 2.24 | 1.80 | 0.62 |
| *mscL* | 592.52 | 694.45 | 1152.81 | 1143.93 | 1.95 | 1.65 | 1.80 | 0.21 |
| *SAUSA300_2642* | 205.52 | 303.27 | 429.79 | 453.90 | 2.09 | 1.50 | 1.79 | 0.42 |
| *femA* | 474.74 | 483.48 | 847.66 | 868.84 | 1.79 | 1.80 | 1.79 | 0.01 |
| *rpoD* | 1272.79 | 1315.95 | 2259.81 | 2371.92 | 1.78 | 1.80 | 1.79 | 0.02 |
| *SAUSA300_0622* | 72.11 | 86.15 | 135.63 | 145.95 | 1.88 | 1.69 | 1.79 | 0.13 |
| *SAUSA300_1691* | 342.53 | 366.57 | 655.22 | 608.26 | 1.91 | 1.66 | 1.79 | 0.18 |
| *SAUSA300_0453* | 579.30 | 566.11 | 1058.43 | 986.51 | 1.83 | 1.74 | 1.78 | 0.06 |
| *SAUSA300_1543* | 54.08 | 79.12 | 119.13 | 107.74 | 2.20 | 1.36 | 1.78 | 0.59 |
| *SAUSA300_2450* | 81.73 | 91.42 | 156.70 | 150.54 | 1.92 | 1.65 | 1.78 | 0.19 |
| *SAUSA300_2407* | 52.88 | 58.90 | 88.89 | 110.80 | 1.68 | 1.88 | 1.78 | 0.14 |
| *gnd* | 2880.89 | 2852.54 | 5201.41 | 5007.46 | 1.81 | 1.76 | 1.78 | 0.04 |
| *SAUSA300_0618* | 6599.50 | 7098.38 | 11908.44 | 12453.32 | 1.80 | 1.75 | 1.78 | 0.04 |
| *int* | 62.50 | 50.11 | 92.56 | 103.92 | 1.48 | 2.07 | 1.78 | 0.42 |
| *SAUSA300_1800* | 320.90 | 314.70 | 534.25 | 592.98 | 1.66 | 1.88 | 1.77 | 0.16 |
| *SAUSA300_1090* | 393.01 | 430.74 | 702.87 | 753.45 | 1.79 | 1.75 | 1.77 | 0.03 |
| *SAUSA300_0035* | 677.86 | 647.86 | 1161.06 | 1176.79 | 1.71 | 1.82 | 1.76 | 0.07 |
| *SAUSA300_0566* | 485.56 | 654.02 | 925.55 | 1056.82 | 1.91 | 1.62 | 1.76 | 0.21 |
| *SAUSA300_0508* | 161.05 | 157.35 | 277.67 | 282.73 | 1.72 | 1.80 | 1.76 | 0.05 |
| *SAUSA300_0288* | 197.11 | 197.79 | 356.47 | 337.75 | 1.81 | 1.71 | 1.76 | 0.07 |
| *SAUSA300_0278* | 622.57 | 697.09 | 1182.14 | 1127.12 | 1.90 | 1.62 | 1.76 | 0.20 |
| *spsA* | 117.78 | 109.00 | 209.85 | 188.74 | 1.78 | 1.73 | 1.76 | 0.04 |
| *SAUSA300_1466* | 681.46 | 583.69 | 1056.59 | 1145.46 | 1.55 | 1.96 | 1.76 | 0.29 |
| *vga* | 97.35 | 101.09 | 190.61 | 156.65 | 1.96 | 1.55 | 1.75 | 0.29 |
| *metB* | 146.63 | 153.83 | 260.25 | 265.92 | 1.77 | 1.73 | 1.75 | 0.03 |
| *SAUSA300_1042* | 144.22 | 138.89 | 252.92 | 241.47 | 1.75 | 1.74 | 1.75 | 0.01 |
| *ychF* | 324.51 | 389.42 | 617.64 | 618.20 | 1.90 | 1.59 | 1.75 | 0.22 |
| *ccrA* | 126.20 | 167.02 | 231.85 | 275.86 | 1.84 | 1.65 | 1.74 | 0.13 |
| *SAUSA300_2080* | 84.13 | 72.96 | 131.04 | 140.60 | 1.56 | 1.93 | 1.74 | 0.26 |
| *SAUSA300_2455* | 414.65 | 405.25 | 681.79 | 745.81 | 1.64 | 1.84 | 1.74 | 0.14 |
| *cysS* | 344.94 | 346.35 | 618.56 | 582.28 | 1.79 | 1.68 | 1.74 | 0.08 |
| *eno* | 9170.31 | 9208.12 | 16058.76 | 15857.60 | 1.75 | 1.72 | 1.74 | 0.02 |
| *SAUSA300_1359* | 405.03 | 461.50 | 731.28 | 768.73 | 1.81 | 1.67 | 1.74 | 0.10 |
| *ulaA* | 387.00 | 215.37 | 491.18 | 473.77 | 1.27 | 2.20 | 1.73 | 0.66 |
| *SAUSA300_1083* | 1240.33 | 1177.94 | 2063.70 | 2109.81 | 1.66 | 1.79 | 1.73 | 0.09 |
| *pbpA* | 1209.09 | 1238.59 | 2243.31 | 1979.91 | 1.86 | 1.60 | 1.73 | 0.18 |
| *SAUSA300_0731* | 191.10 | 212.73 | 339.98 | 353.04 | 1.78 | 1.66 | 1.72 | 0.08 |
| *pheT* | 1364.13 | 1367.81 | 2307.46 | 2389.49 | 1.69 | 1.75 | 1.72 | 0.04 |
| *SAUSA300_1850* | 522.82 | 532.71 | 923.72 | 889.47 | 1.77 | 1.67 | 1.72 | 0.07 |
| *SAUSA300_2297* | 198.31 | 216.25 | 341.81 | 369.85 | 1.72 | 1.71 | 1.72 | 0.01 |
| *SAUSA300_0619* | 3949.36 | 4417.26 | 6986.53 | 7306.02 | 1.77 | 1.65 | 1.71 | 0.08 |
| *nagA* | 174.27 | 210.09 | 316.15 | 337.75 | 1.81 | 1.61 | 1.71 | 0.15 |
| *SAUSA300_2330* | 117.78 | 117.79 | 203.44 | 198.68 | 1.73 | 1.69 | 1.71 | 0.03 |
| *hemH* | 325.71 | 339.32 | 524.17 | 611.32 | 1.61 | 1.80 | 1.71 | 0.14 |
| *ligA* | 432.67 | 519.52 | 822.92 | 777.90 | 1.90 | 1.50 | 1.70 | 0.29 |
| *SAUSA300_0376* | 123.79 | 87.91 | 179.61 | 171.17 | 1.45 | 1.95 | 1.70 | 0.35 |
| *SAUSA300_1975* | 682.66 | 875.54 | 1379.16 | 1205.83 | 2.02 | 1.38 | 1.70 | 0.45 |
| *xseA* | 729.54 | 789.39 | 1286.61 | 1289.12 | 1.76 | 1.63 | 1.70 | 0.09 |
| *SAUSA300_2578* | 1222.31 | 1242.99 | 1950.99 | 2237.43 | 1.60 | 1.80 | 1.70 | 0.14 |
| *rpmE2* | 3596.01 | 3699.07 | 6435.78 | 5934.37 | 1.79 | 1.60 | 1.70 | 0.13 |
| *SAUSA300_1786* | 338.93 | 399.97 | 624.98 | 618.20 | 1.84 | 1.55 | 1.69 | 0.21 |
| *SAUSA300_1894* | 185.09 | 207.46 | 321.65 | 339.28 | 1.74 | 1.64 | 1.69 | 0.07 |
| *rplM* | 2258.32 | 2219.62 | 3858.90 | 3687.78 | 1.71 | 1.66 | 1.69 | 0.03 |
| *argR* | 88.94 | 67.69 | 112.72 | 142.13 | 1.27 | 2.10 | 1.68 | 0.59 |
| *SAUSA300_1351* | 2956.61 | 2913.19 | 5020.88 | 4835.53 | 1.70 | 1.66 | 1.68 | 0.03 |
| *moaB* | 231.96 | 260.20 | 414.21 | 405.76 | 1.79 | 1.56 | 1.67 | 0.16 |
| *SAUSA300_1336* | 150.23 | 216.25 | 295.08 | 298.02 | 1.96 | 1.38 | 1.67 | 0.41 |
| *SAUSA300_1707* | 121.39 | 120.43 | 170.45 | 233.07 | 1.40 | 1.94 | 1.67 | 0.38 |
| *SAUSA300_1515* | 167.06 | 157.35 | 280.41 | 261.34 | 1.68 | 1.66 | 1.67 | 0.01 |
| *SAUSA300_1877* | 133.41 | 181.09 | 268.50 | 239.94 | 2.01 | 1.33 | 1.67 | 0.49 |
| *isaB* | 618.97 | 627.65 | 1141.82 | 932.26 | 1.84 | 1.49 | 1.67 | 0.25 |
| *SAUSA300_0183* | 818.48 | 932.68 | 1488.21 | 1406.03 | 1.82 | 1.51 | 1.66 | 0.22 |
| *pheA* | 1080.49 | 1098.82 | 1902.42 | 1714.75 | 1.76 | 1.56 | 1.66 | 0.14 |
| *SAUSA300_1085* | 222.35 | 230.31 | 372.05 | 379.02 | 1.67 | 1.65 | 1.66 | 0.02 |
| *SAUSA300_0866* | 127.40 | 132.74 | 218.10 | 213.20 | 1.71 | 1.61 | 1.66 | 0.07 |
| *SAUSA300_1728* | 108.17 | 130.98 | 197.02 | 194.86 | 1.82 | 1.49 | 1.65 | 0.24 |
| *scrB* | 335.32 | 319.10 | 516.84 | 563.94 | 1.54 | 1.77 | 1.65 | 0.16 |
| *SAUSA300_2585* | 153.84 | 182.84 | 295.99 | 252.17 | 1.92 | 1.38 | 1.65 | 0.39 |
| *SAUSA300_0637* | 84.13 | 79.99 | 129.21 | 141.37 | 1.54 | 1.77 | 1.65 | 0.16 |
| *rpmH* | 2718.64 | 2608.16 | 4751.46 | 4047.69 | 1.75 | 1.55 | 1.65 | 0.14 |
| *SAUSA300_2381* | 94.95 | 104.61 | 201.61 | 123.03 | 2.12 | 1.18 | 1.65 | 0.67 |
| *SAUSA300_2468* | 67.30 | 65.05 | 123.71 | 94.75 | 1.84 | 1.46 | 1.65 | 0.27 |
| *SAUSA300_1274* | 63.70 | 63.29 | 103.55 | 105.45 | 1.63 | 1.67 | 1.65 | 0.03 |
| *SAUSA300_1082* | 1044.43 | 1036.41 | 1705.40 | 1715.51 | 1.63 | 1.66 | 1.64 | 0.02 |
| *SAUSA300_0254* | 725.93 | 918.61 | 1207.80 | 1483.21 | 1.66 | 1.61 | 1.64 | 0.03 |
| *SAUSA300_0574* | 475.94 | 437.77 | 745.02 | 747.34 | 1.57 | 1.71 | 1.64 | 0.10 |
| *scpA* | 126.20 | 167.90 | 246.51 | 220.84 | 1.95 | 1.32 | 1.63 | 0.45 |
| *secA* | 2566.00 | 2480.70 | 4118.24 | 4126.40 | 1.60 | 1.66 | 1.63 | 0.04 |
| *SAUSA300_0541* | 93.75 | 65.05 | 136.54 | 117.68 | 1.46 | 1.81 | 1.63 | 0.25 |
| *birA* | 135.81 | 154.71 | 230.01 | 243.00 | 1.69 | 1.57 | 1.63 | 0.09 |
| *SAUSA300_1100* | 290.85 | 289.21 | 514.09 | 432.51 | 1.77 | 1.50 | 1.63 | 0.19 |
| *gltX* | 890.59 | 771.81 | 1358.08 | 1337.26 | 1.52 | 1.73 | 1.63 | 0.15 |
| *SAUSA300_0180* | 201.91 | 194.27 | 274.00 | 368.32 | 1.36 | 1.90 | 1.63 | 0.38 |
| *tuf* | 41013.98 | 38784.87 | 64301.00 | 64338.96 | 1.57 | 1.66 | 1.61 | 0.06 |
| *SAUSA300_1551* | 112.98 | 137.13 | 201.61 | 197.15 | 1.78 | 1.44 | 1.61 | 0.25 |
| *SAUSA300_1584* | 634.59 | 602.15 | 990.61 | 997.21 | 1.56 | 1.66 | 1.61 | 0.07 |
| *SAUSA300_1260* | 149.03 | 147.68 | 252.01 | 224.66 | 1.69 | 1.52 | 1.61 | 0.12 |
| *SAUSA300_2162* | 99.76 | 89.66 | 145.71 | 156.65 | 1.46 | 1.75 | 1.60 | 0.20 |
| *SAUSA300_1806* | 138.22 | 171.42 | 251.09 | 238.41 | 1.82 | 1.39 | 1.60 | 0.30 |
| *SAUSA300_2214* | 454.31 | 404.37 | 708.37 | 665.57 | 1.56 | 1.65 | 1.60 | 0.06 |
| *SAUSA300_2267* | 91.34 | 100.21 | 145.71 | 161.24 | 1.60 | 1.61 | 1.60 | 0.01 |
| *SAUSA300_1348* | 161.05 | 153.83 | 224.51 | 276.62 | 1.39 | 1.80 | 1.60 | 0.29 |
| *SAUSA300_1688* | 112.98 | 98.45 | 142.96 | 189.51 | 1.27 | 1.92 | 1.60 | 0.47 |
| *SAUSA300_1478* | 249.99 | 183.72 | 348.23 | 328.58 | 1.39 | 1.79 | 1.59 | 0.28 |
| *gyrB* | 1653.78 | 1813.49 | 2715.25 | 2789.14 | 1.64 | 1.54 | 1.59 | 0.07 |
| *holA* | 94.95 | 89.66 | 140.21 | 152.07 | 1.48 | 1.70 | 1.59 | 0.16 |
| *kdpD* | 132.21 | 150.32 | 212.60 | 234.59 | 1.61 | 1.56 | 1.58 | 0.03 |
| *thiD* | 706.70 | 674.24 | 1092.33 | 1093.50 | 1.55 | 1.62 | 1.58 | 0.05 |
| *SAUSA300_0874* | 170.67 | 168.78 | 251.09 | 285.03 | 1.47 | 1.69 | 1.58 | 0.15 |
| *SAUSA300_2070* | 258.40 | 266.35 | 399.54 | 429.45 | 1.55 | 1.61 | 1.58 | 0.05 |
| *SAUSA300_0554* | 207.92 | 167.90 | 249.26 | 327.82 | 1.20 | 1.95 | 1.58 | 0.53 |
| *SAUSA300_1084* | 438.68 | 435.13 | 679.96 | 696.14 | 1.55 | 1.60 | 1.57 | 0.04 |
| *SAUSA300_2113* | 539.64 | 583.69 | 821.08 | 948.31 | 1.52 | 1.62 | 1.57 | 0.07 |
| *SAUSA300_0857* | 311.29 | 312.94 | 498.51 | 479.12 | 1.60 | 1.53 | 1.57 | 0.05 |
| *mnhA* | 2162.17 | 2095.67 | 3383.30 | 3275.90 | 1.56 | 1.56 | 1.56 | 0.00 |
| *SAUSA300_1338* | 713.91 | 741.92 | 1115.24 | 1159.98 | 1.56 | 1.56 | 1.56 | 0.00 |
| *SAUSA300_1673* | 236.77 | 211.85 | 331.73 | 363.73 | 1.40 | 1.72 | 1.56 | 0.22 |
| *nusB* | 326.91 | 356.90 | 522.34 | 541.02 | 1.60 | 1.52 | 1.56 | 0.06 |
| *SAUSA300_0687* | 921.84 | 986.30 | 1402.07 | 1569.56 | 1.52 | 1.59 | 1.56 | 0.05 |
| *SAUSA300_2174* | 109.37 | 113.40 | 164.95 | 181.87 | 1.51 | 1.60 | 1.56 | 0.07 |
| *SAUSA300_0381* | 108.17 | 126.58 | 183.28 | 178.05 | 1.69 | 1.41 | 1.55 | 0.20 |
| *SAUSA300_0726* | 216.34 | 187.24 | 304.24 | 317.12 | 1.41 | 1.69 | 1.55 | 0.20 |
| *SAUSA300_1699* | 341.33 | 370.08 | 558.08 | 540.25 | 1.64 | 1.46 | 1.55 | 0.12 |
| *SAUSA300_2622* | 103.36 | 107.24 | 161.28 | 164.29 | 1.56 | 1.53 | 1.55 | 0.02 |
| *SAUSA300_2125* | 555.27 | 550.29 | 845.82 | 862.72 | 1.52 | 1.57 | 1.55 | 0.03 |
| *murA* | 2645.33 | 2532.56 | 3944.13 | 4043.87 | 1.49 | 1.60 | 1.54 | 0.07 |
| *SAUSA300_2322* | 497.58 | 534.47 | 839.41 | 748.10 | 1.69 | 1.40 | 1.54 | 0.20 |
| *SAUSA300_0495* | 98.55 | 115.16 | 164.03 | 162.76 | 1.66 | 1.41 | 1.54 | 0.18 |
| *SAUSA300_0362* | 141.82 | 103.73 | 197.02 | 174.99 | 1.39 | 1.69 | 1.54 | 0.21 |
| *moeB* | 402.63 | 465.02 | 657.05 | 669.39 | 1.63 | 1.44 | 1.54 | 0.14 |
| *SAUSA300_1654* | 909.82 | 950.26 | 1509.29 | 1341.84 | 1.66 | 1.41 | 1.54 | 0.17 |
| *galM* | 294.46 | 312.94 | 431.62 | 499.75 | 1.47 | 1.60 | 1.53 | 0.09 |
| *SAUSA300_0231* | 159.85 | 217.13 | 278.58 | 285.79 | 1.74 | 1.32 | 1.53 | 0.30 |
| *SAUSA300_1172* | 281.24 | 292.73 | 433.45 | 443.97 | 1.54 | 1.52 | 1.53 | 0.02 |
| *ctaA* | 1389.37 | 1392.43 | 2172.75 | 2076.19 | 1.56 | 1.49 | 1.53 | 0.05 |
| *SAUSA300_0828* | 183.89 | 237.35 | 325.32 | 302.60 | 1.77 | 1.27 | 1.52 | 0.35 |
| *tag* | 143.02 | 157.35 | 244.68 | 208.61 | 1.71 | 1.33 | 1.52 | 0.27 |
| *SAUSA300_0725* | 147.83 | 158.23 | 230.01 | 233.83 | 1.56 | 1.48 | 1.52 | 0.06 |
| *SAUSA300_2394* | 284.84 | 254.05 | 438.95 | 379.02 | 1.54 | 1.49 | 1.52 | 0.03 |
| *SAUSA300_0745* | 106.97 | 99.33 | 126.46 | 183.40 | 1.18 | 1.85 | 1.51 | 0.47 |
| *SAUSA300_0746* | 293.26 | 288.33 | 427.95 | 452.38 | 1.46 | 1.57 | 1.51 | 0.08 |
| *SAUSA300_0033* | 4510.64 | 4723.17 | 7150.56 | 6795.57 | 1.59 | 1.44 | 1.51 | 0.10 |
| *SAUSA300_0899* | 1139.38 | 1093.55 | 1717.31 | 1658.20 | 1.51 | 1.52 | 1.51 | 0.01 |
| *recN* | 368.98 | 337.56 | 510.43 | 550.19 | 1.38 | 1.63 | 1.51 | 0.17 |
| *pbp4* | 224.75 | 216.25 | 297.83 | 364.50 | 1.33 | 1.69 | 1.51 | 0.25 |
| *lipA* | 1497.54 | 1409.13 | 2054.54 | 2306.20 | 1.37 | 1.64 | 1.50 | 0.19 |
| *rluB* | 192.30 | 216.25 | 275.83 | 340.05 | 1.43 | 1.57 | 1.50 | 0.10 |
| *SAUSA300_1494* | 394.21 | 487.00 | 623.14 | 693.85 | 1.58 | 1.42 | 1.50 | 0.11 |
| *folC* | 1070.87 | 1223.65 | 1620.17 | 1807.97 | 1.51 | 1.48 | 1.50 | 0.03 |
| *hemL* | 2829.21 | 2870.12 | 4285.94 | 4231.09 | 1.51 | 1.47 | 1.49 | 0.03 |
| *SAUSA300_2068* | 2066.02 | 1874.15 | 2881.12 | 2980.94 | 1.39 | 1.59 | 1.49 | 0.14 |
| *fusA* | 38093.43 | 37007.42 | 55652.15 | 56404.05 | 1.46 | 1.52 | 1.49 | 0.04 |
| *mnhB* | 355.75 | 356.02 | 537.92 | 521.91 | 1.51 | 1.47 | 1.49 | 0.03 |
| *mnhF* | 115.38 | 117.79 | 182.36 | 164.29 | 1.58 | 1.39 | 1.49 | 0.13 |
| *ksgA* | 126.20 | 151.20 | 200.69 | 205.56 | 1.59 | 1.36 | 1.47 | 0.16 |
| *engA* | 896.60 | 923.89 | 1377.33 | 1304.40 | 1.54 | 1.41 | 1.47 | 0.09 |
| *glpT* | 175.47 | 162.63 | 252.01 | 245.29 | 1.44 | 1.51 | 1.47 | 0.05 |
| *lysS* | 2219.86 | 2201.16 | 3248.59 | 3255.27 | 1.46 | 1.48 | 1.47 | 0.01 |
| *SAUSA300_1118* | 187.49 | 185.48 | 235.51 | 310.24 | 1.26 | 1.67 | 1.46 | 0.29 |
| *nagE* | 892.99 | 905.43 | 1227.96 | 1402.98 | 1.38 | 1.55 | 1.46 | 0.12 |
| *SAUSA300_0429* | 168.26 | 180.21 | 280.41 | 226.19 | 1.67 | 1.26 | 1.46 | 0.29 |
| *SAUSA300_1332* | 264.41 | 272.51 | 431.62 | 349.98 | 1.63 | 1.28 | 1.46 | 0.25 |
| *SAUSA300_0924* | 546.85 | 643.47 | 866.90 | 855.08 | 1.59 | 1.33 | 1.46 | 0.18 |
| *hemG* | 1474.70 | 1689.55 | 2191.08 | 2409.36 | 1.49 | 1.43 | 1.46 | 0.04 |
| *SAUSA300_1155* | 406.23 | 369.20 | 586.49 | 541.78 | 1.44 | 1.47 | 1.46 | 0.02 |
| *xerC* | 362.97 | 384.15 | 583.74 | 498.22 | 1.61 | 1.30 | 1.45 | 0.22 |
| *murB* | 251.19 | 260.20 | 369.30 | 372.90 | 1.47 | 1.43 | 1.45 | 0.03 |
| *grpE* | 638.20 | 684.79 | 932.88 | 983.46 | 1.46 | 1.44 | 1.45 | 0.02 |
| *SAUSA300_2586* | 236.77 | 298.88 | 390.38 | 372.90 | 1.65 | 1.25 | 1.45 | 0.28 |
| *SAUSA300_1519* | 307.68 | 306.79 | 475.60 | 414.17 | 1.55 | 1.35 | 1.45 | 0.14 |
| *SAUSA300_0754* | 181.48 | 236.47 | 317.99 | 268.98 | 1.75 | 1.14 | 1.44 | 0.43 |
| *prs* | 1504.75 | 1371.33 | 2004.14 | 2134.27 | 1.33 | 1.56 | 1.44 | 0.16 |
| *SAUSA300_2112* | 502.38 | 536.22 | 730.36 | 766.44 | 1.45 | 1.43 | 1.44 | 0.02 |
| *gltT* | 1448.26 | 1628.89 | 2243.31 | 2166.36 | 1.55 | 1.33 | 1.44 | 0.15 |
| *SAUSA300_0703* | 5895.20 | 5620.69 | 8174.17 | 8381.94 | 1.39 | 1.49 | 1.44 | 0.07 |
| *spsB* | 159.85 | 169.66 | 253.84 | 216.25 | 1.59 | 1.27 | 1.43 | 0.22 |
| *SAUSA300_2360* | 203.12 | 209.22 | 307.91 | 280.44 | 1.52 | 1.34 | 1.43 | 0.12 |
| *SAUSA300_1516* | 219.94 | 182.84 | 279.50 | 288.08 | 1.27 | 1.58 | 1.42 | 0.22 |
| *SAUSA300_0430* | 241.58 | 261.96 | 373.89 | 340.05 | 1.55 | 1.30 | 1.42 | 0.18 |
| *mnhG* | 402.63 | 450.96 | 663.46 | 539.49 | 1.65 | 1.20 | 1.42 | 0.32 |
| *dnaN* | 1889.35 | 1915.46 | 2639.19 | 2770.80 | 1.40 | 1.45 | 1.42 | 0.04 |
| *SAUSA300_1465* | 972.32 | 809.61 | 1359.00 | 1157.68 | 1.40 | 1.43 | 1.41 | 0.02 |
| *arcA* | 792.04 | 845.65 | 1129.90 | 1176.79 | 1.43 | 1.39 | 1.41 | 0.02 |
| *SAUSA300_0512* | 443.49 | 425.46 | 591.99 | 629.66 | 1.33 | 1.48 | 1.41 | 0.10 |
| *glpP* | 164.66 | 180.21 | 264.84 | 217.02 | 1.61 | 1.20 | 1.41 | 0.29 |
| *metK* | 1118.95 | 992.46 | 1460.72 | 1493.91 | 1.31 | 1.51 | 1.41 | 0.14 |
| *valS* | 1703.06 | 1704.49 | 2398.18 | 2381.08 | 1.41 | 1.40 | 1.40 | 0.01 |
| *SAUSA300_1460* | 271.62 | 239.98 | 351.89 | 362.21 | 1.30 | 1.51 | 1.40 | 0.15 |
| *SAUSA300_1690* | 256.00 | 219.76 | 356.47 | 310.24 | 1.39 | 1.41 | 1.40 | 0.01 |
| *SAUSA300_0217* | 246.38 | 246.14 | 347.31 | 342.34 | 1.41 | 1.39 | 1.40 | 0.01 |
| *rpsA* | 5714.92 | 5255.88 | 8021.13 | 7260.17 | 1.40 | 1.38 | 1.39 | 0.02 |
| *SAUSA300_1906* | 594.93 | 610.07 | 838.49 | 838.27 | 1.41 | 1.37 | 1.39 | 0.02 |
| *SAUSA300_1322* | 645.41 | 583.69 | 820.17 | 881.83 | 1.27 | 1.51 | 1.39 | 0.17 |
| *SAUSA300_1705* | 332.92 | 334.92 | 457.28 | 471.48 | 1.37 | 1.41 | 1.39 | 0.02 |
| *SAUSA300_0656* | 540.84 | 554.68 | 760.60 | 761.86 | 1.41 | 1.37 | 1.39 | 0.02 |
| *glyA* | 4259.44 | 4055.09 | 5622.95 | 5911.45 | 1.32 | 1.46 | 1.39 | 0.10 |
| *SAUSA300_1474* | 290.85 | 244.38 | 342.73 | 388.19 | 1.18 | 1.59 | 1.38 | 0.29 |
| *SAUSA300_2102* | 325.71 | 268.11 | 409.62 | 403.47 | 1.26 | 1.50 | 1.38 | 0.17 |
| *SAUSA300_2397* | 293.26 | 338.44 | 425.20 | 443.97 | 1.45 | 1.31 | 1.38 | 0.10 |
| *SAUSA300_0655* | 761.99 | 699.73 | 994.28 | 1019.37 | 1.30 | 1.46 | 1.38 | 0.11 |
| *cobI* | 524.02 | 540.62 | 708.37 | 761.09 | 1.35 | 1.41 | 1.38 | 0.04 |
| *gpsA* | 1135.77 | 1235.95 | 1676.99 | 1583.31 | 1.48 | 1.28 | 1.38 | 0.14 |
| *rpsS* | 1618.93 | 1658.78 | 2246.98 | 2250.42 | 1.39 | 1.36 | 1.37 | 0.02 |
| *aldA2* | 419.45 | 388.54 | 608.48 | 500.52 | 1.45 | 1.29 | 1.37 | 0.11 |
| *rpoB* | 17779.34 | 17365.77 | 23184.58 | 24811.12 | 1.30 | 1.43 | 1.37 | 0.09 |
| *SAUSA300_1070* | 179.08 | 187.24 | 257.50 | 242.23 | 1.44 | 1.29 | 1.37 | 0.10 |
| *mvaD* | 305.28 | 341.95 | 425.20 | 456.96 | 1.39 | 1.34 | 1.36 | 0.04 |
| *glmM* | 1574.46 | 1411.77 | 2118.69 | 1950.11 | 1.35 | 1.38 | 1.36 | 0.03 |
| *SAUSA300_1337* | 766.80 | 859.72 | 1096.91 | 1111.84 | 1.43 | 1.29 | 1.36 | 0.10 |
| *SAUSA300_1785* | 633.39 | 695.33 | 912.72 | 889.47 | 1.44 | 1.28 | 1.36 | 0.11 |
| *recR* | 971.11 | 922.13 | 1266.45 | 1289.88 | 1.30 | 1.40 | 1.35 | 0.07 |
| *SAUSA300_0555* | 451.90 | 363.93 | 529.67 | 550.19 | 1.17 | 1.51 | 1.34 | 0.24 |
| *efp* | 2150.15 | 2173.91 | 2871.96 | 2925.92 | 1.34 | 1.35 | 1.34 | 0.01 |
| *SAUSA300_0526* | 281.24 | 261.08 | 331.73 | 391.24 | 1.18 | 1.50 | 1.34 | 0.23 |
| *prmA* | 534.83 | 482.60 | 714.78 | 644.94 | 1.34 | 1.34 | 1.34 | 0.00 |
| *dnaX* | 831.70 | 850.05 | 1139.07 | 1104.96 | 1.37 | 1.30 | 1.33 | 0.05 |
| *SAUSA300_0046* | 510.80 | 491.39 | 740.44 | 596.04 | 1.45 | 1.21 | 1.33 | 0.17 |
| *mnhC* | 366.57 | 373.60 | 493.93 | 486.76 | 1.35 | 1.30 | 1.33 | 0.03 |
| *ftsY* | 264.41 | 268.99 | 350.98 | 354.56 | 1.33 | 1.32 | 1.32 | 0.01 |
| *SAUSA300_2282* | 364.17 | 353.38 | 444.45 | 502.81 | 1.22 | 1.42 | 1.32 | 0.14 |
| *rplV* | 1132.17 | 1135.74 | 1464.39 | 1528.30 | 1.29 | 1.35 | 1.32 | 0.04 |
| *SAUSA300_1902* | 449.50 | 428.98 | 552.58 | 599.09 | 1.23 | 1.40 | 1.31 | 0.12 |
| *SAUSA300_2643* | 580.51 | 596.88 | 793.59 | 750.39 | 1.37 | 1.26 | 1.31 | 0.08 |
| *queA* | 419.45 | 516.01 | 615.81 | 595.27 | 1.47 | 1.15 | 1.31 | 0.22 |
| *SAUSA300_0844* | 2203.04 | 1940.08 | 2601.62 | 2780.73 | 1.18 | 1.43 | 1.31 | 0.18 |
| *nusA* | 593.73 | 516.01 | 743.19 | 688.50 | 1.25 | 1.33 | 1.29 | 0.06 |
| *SAUSA300_2259* | 757.18 | 792.03 | 1018.11 | 980.40 | 1.34 | 1.24 | 1.29 | 0.08 |
| *SAUSA300_0733* | 1044.43 | 1183.21 | 1424.06 | 1439.65 | 1.36 | 1.22 | 1.29 | 0.10 |
| *recF* | 1081.69 | 1075.97 | 1363.58 | 1418.26 | 1.26 | 1.32 | 1.29 | 0.04 |
| *SAUSA300_0648* | 384.60 | 406.12 | 511.34 | 506.63 | 1.33 | 1.25 | 1.29 | 0.06 |
| *saeS* | 1467.49 | 1566.48 | 2141.60 | 1739.20 | 1.46 | 1.11 | 1.28 | 0.25 |
| *mnhD* | 1703.06 | 1815.25 | 2361.53 | 2136.56 | 1.39 | 1.18 | 1.28 | 0.15 |
| *serS* | 1763.15 | 1330.01 | 1901.50 | 1973.79 | 1.08 | 1.48 | 1.28 | 0.29 |
| *SAUSA300_2589* | 6913.18 | 6737.97 | 8685.51 | 8778.53 | 1.26 | 1.30 | 1.28 | 0.03 |
| *SAUSA300_1464* | 1478.31 | 1388.91 | 1921.66 | 1742.26 | 1.30 | 1.25 | 1.28 | 0.03 |
| *SAUSA300_2359* | 1278.79 | 1359.90 | 1624.75 | 1703.29 | 1.27 | 1.25 | 1.26 | 0.01 |
| *SAUSA300_0468* | 1140.58 | 1367.81 | 1520.29 | 1619.23 | 1.33 | 1.18 | 1.26 | 0.11 |
| *SAUSA300_0252* | 3658.51 | 3685.89 | 4502.21 | 4738.48 | 1.23 | 1.29 | 1.26 | 0.04 |
| *SAUSA300_1252* | 755.98 | 839.50 | 1006.19 | 993.39 | 1.33 | 1.18 | 1.26 | 0.10 |
| *SAUSA300_0617* | 1230.72 | 1207.82 | 1460.72 | 1532.12 | 1.19 | 1.27 | 1.23 | 0.06 |
| *rplI* | 1180.24 | 1091.79 | 1440.56 | 1345.66 | 1.22 | 1.23 | 1.23 | 0.01 |
| *mecA* | 24972.56 | 23084.03 | 30145.45 | 27487.93 | 1.21 | 1.19 | 1.20 | 0.01 |
| *rpsC* | 3166.94 | 3154.06 | 3740.69 | 3823.80 | 1.18 | 1.21 | 1.20 | 0.02 |
| *SAUSA300_0989* | 3787.11 | 3380.85 | 4191.55 | 4322.78 | 1.11 | 1.28 | 1.19 | 0.12 |
| *pnpA* | 2471.05 | 2488.61 | 3101.97 | 2791.43 | 1.26 | 1.12 | 1.19 | 0.09 |
| *SAUSA300_1642* | 3318.38 | 3318.44 | 3882.73 | 3956.76 | 1.17 | 1.19 | 1.18 | 0.02 |
| *atpD* | 21574.86 | 21146.59 | 18305.74 | 17880.30 | -1.18 | -1.18 | -1.18 | 0.00 |
| *rpoA* | 9271.26 | 9590.51 | 7881.84 | 8041.89 | -1.18 | -1.19 | -1.18 | 0.01 |
| *SAUSA300_2077* | 2479.47 | 2622.23 | 2135.18 | 2123.57 | -1.16 | -1.23 | -1.20 | 0.05 |
| *rpsM* | 2921.76 | 2996.70 | 2428.42 | 2467.43 | -1.20 | -1.21 | -1.21 | 0.01 |
| *zwf* | 1459.08 | 1522.53 | 1242.62 | 1204.30 | -1.17 | -1.26 | -1.22 | 0.06 |
| *rplO* | 3801.53 | 3808.95 | 3156.03 | 3025.26 | -1.20 | -1.26 | -1.23 | 0.04 |
| *SAUSA300_0022* | 3064.78 | 2653.87 | 2268.97 | 2366.57 | -1.35 | -1.12 | -1.24 | 0.16 |
| *rpsK* | 2682.58 | 2605.52 | 2185.58 | 2070.08 | -1.23 | -1.26 | -1.24 | 0.02 |
| *SAUSA300_1168* | 3812.35 | 3476.67 | 2931.52 | 2907.58 | -1.30 | -1.20 | -1.25 | 0.07 |
| *SAUSA300_0980* | 2398.94 | 2442.02 | 1999.56 | 1880.57 | -1.20 | -1.30 | -1.25 | 0.07 |
| *SAUSA300_2582* | 963.90 | 923.89 | 757.85 | 748.86 | -1.27 | -1.23 | -1.25 | 0.03 |
| *ppaC* | 3902.49 | 3224.38 | 2893.03 | 2783.79 | -1.35 | -1.16 | -1.25 | 0.13 |
| *SAUSA300_1651* | 931.45 | 1002.12 | 734.94 | 796.24 | -1.27 | -1.26 | -1.26 | 0.01 |
| *murF* | 1076.88 | 1085.64 | 876.07 | 801.59 | -1.23 | -1.35 | -1.29 | 0.09 |
| *obgE* | 1073.27 | 1065.42 | 824.75 | 822.99 | -1.30 | -1.29 | -1.30 | 0.00 |
| *SAUSA300_0013* | 628.58 | 535.35 | 452.69 | 441.68 | -1.39 | -1.21 | -1.30 | 0.12 |
| *atpF* | 4630.82 | 4799.65 | 3581.24 | 3655.68 | -1.29 | -1.31 | -1.30 | 0.01 |
| *pepT* | 1189.86 | 1201.67 | 869.65 | 966.65 | -1.37 | -1.24 | -1.31 | 0.09 |
| *SAUSA300_0181* | 5406.03 | 5496.74 | 4128.32 | 4217.33 | -1.31 | -1.30 | -1.31 | 0.00 |
| *era* | 695.89 | 715.55 | 611.23 | 485.23 | -1.14 | -1.47 | -1.31 | 0.24 |
| *SAUSA300_0630* | 3983.01 | 3691.16 | 2882.95 | 2994.70 | -1.38 | -1.23 | -1.31 | 0.11 |
| *rpmJ* | 624.97 | 653.14 | 537.92 | 449.32 | -1.16 | -1.45 | -1.31 | 0.21 |
| *rplE* | 3216.22 | 3241.08 | 2476.08 | 2459.79 | -1.30 | -1.32 | -1.31 | 0.01 |
| *SAUSA300_0426* | 347.34 | 357.78 | 260.25 | 277.39 | -1.33 | -1.29 | -1.31 | 0.03 |
| *pepS* | 525.22 | 480.84 | 372.97 | 395.06 | -1.41 | -1.22 | -1.31 | 0.14 |
| *glpQ* | 635.79 | 727.86 | 476.52 | 560.88 | -1.33 | -1.30 | -1.32 | 0.03 |
| *atpA* | 12683.39 | 12434.26 | 9636.72 | 9447.92 | -1.32 | -1.32 | -1.32 | 0.00 |
| *infA* | 2392.93 | 2617.83 | 1900.59 | 1903.49 | -1.26 | -1.38 | -1.32 | 0.08 |
| *ftsA* | 5728.14 | 5811.45 | 4277.69 | 4484.78 | -1.34 | -1.30 | -1.32 | 0.03 |
| *SAUSA300_0182* | 552.86 | 616.22 | 449.03 | 438.62 | -1.23 | -1.40 | -1.32 | 0.12 |
| *rpmD* | 1658.59 | 1568.24 | 1208.71 | 1227.22 | -1.37 | -1.28 | -1.33 | 0.07 |
| *SAUSA300_0483* | 727.13 | 772.69 | 526.01 | 608.26 | -1.38 | -1.27 | -1.33 | 0.08 |
| *SAUSA300_0485* | 499.98 | 500.18 | 420.62 | 336.99 | -1.19 | -1.48 | -1.34 | 0.21 |
| *SAUSA300_2556* | 374.98 | 367.45 | 284.08 | 271.27 | -1.32 | -1.35 | -1.34 | 0.02 |
| *gcvH* | 823.28 | 799.06 | 623.14 | 583.04 | -1.32 | -1.37 | -1.35 | 0.03 |
| *SAUSA300_0536* | 1972.28 | 1865.36 | 1458.89 | 1386.93 | -1.35 | -1.34 | -1.35 | 0.00 |
| *SAUSA300_2554* | 456.71 | 463.26 | 319.82 | 360.68 | -1.43 | -1.28 | -1.36 | 0.10 |
| *ebpS* | 1437.44 | 1412.64 | 1098.75 | 1004.85 | -1.31 | -1.41 | -1.36 | 0.07 |
| *mobB* | 374.98 | 530.07 | 335.40 | 331.64 | -1.12 | -1.60 | -1.36 | 0.34 |
| *infB* | 4600.78 | 4607.14 | 3517.09 | 3248.39 | -1.31 | -1.42 | -1.36 | 0.08 |
| *clfB* | 3678.94 | 3708.74 | 2852.71 | 2571.36 | -1.29 | -1.44 | -1.37 | 0.11 |
| *SAUSA300_2128* | 413.44 | 483.48 | 311.57 | 343.87 | -1.33 | -1.41 | -1.37 | 0.06 |
| *sun* | 713.91 | 690.94 | 505.85 | 519.62 | -1.41 | -1.33 | -1.37 | 0.06 |
| *modC* | 961.50 | 864.99 | 727.61 | 609.03 | -1.32 | -1.42 | -1.37 | 0.07 |
| *SAUSA300_0021* | 3312.37 | 3214.71 | 2273.55 | 2491.89 | -1.46 | -1.29 | -1.37 | 0.12 |
| *recA* | 2144.14 | 1912.83 | 1556.02 | 1396.86 | -1.38 | -1.37 | -1.37 | 0.01 |
| *qoxC* | 24342.78 | 23629.93 | 17440.67 | 17343.10 | -1.40 | -1.36 | -1.38 | 0.02 |
| *SAUSA300_2423* | 42021.15 | 37950.64 | 28448.30 | 29329.52 | -1.48 | -1.29 | -1.39 | 0.13 |
| *SAUSA300_0939* | 346.14 | 317.34 | 238.26 | 240.71 | -1.45 | -1.32 | -1.39 | 0.10 |
| *srrA* | 1396.58 | 1255.29 | 970.45 | 938.37 | -1.44 | -1.34 | -1.39 | 0.07 |
| *rpsE* | 4475.78 | 4480.55 | 3266.92 | 3147.53 | -1.37 | -1.42 | -1.40 | 0.04 |
| *SAUSA300_1497* | 870.16 | 768.30 | 551.66 | 631.19 | -1.58 | -1.22 | -1.40 | 0.25 |
| *SAUSA300_2129* | 772.81 | 833.35 | 540.67 | 609.03 | -1.43 | -1.37 | -1.40 | 0.04 |
| *qoxD* | 9033.29 | 8705.30 | 6251.59 | 6393.63 | -1.44 | -1.36 | -1.40 | 0.06 |
| *rpoZ* | 263.21 | 285.69 | 189.69 | 200.97 | -1.39 | -1.42 | -1.40 | 0.02 |
| *SAUSA300_1003* | 2207.84 | 2166.00 | 1619.25 | 1490.85 | -1.36 | -1.45 | -1.41 | 0.06 |
| *rexA* | 2099.68 | 2216.10 | 1488.21 | 1574.14 | -1.41 | -1.41 | -1.41 | 0.00 |
| *glpK* | 1437.44 | 1288.70 | 972.29 | 959.01 | -1.48 | -1.34 | -1.41 | 0.10 |
| *atpH* | 3879.65 | 3861.70 | 2805.98 | 2679.10 | -1.38 | -1.44 | -1.41 | 0.04 |
| *fruA* | 1699.45 | 1628.89 | 1164.73 | 1192.83 | -1.46 | -1.37 | -1.41 | 0.07 |
| *SAUSA300_0176* | 286.05 | 363.93 | 242.84 | 220.84 | -1.18 | -1.65 | -1.41 | 0.33 |
| *SAUSA300_1231* | 387.00 | 370.96 | 260.25 | 275.86 | -1.49 | -1.34 | -1.42 | 0.10 |
| *moaE* | 286.05 | 407.88 | 227.26 | 259.05 | -1.26 | -1.57 | -1.42 | 0.22 |
| *SAUSA300_2583* | 900.20 | 868.51 | 614.90 | 632.71 | -1.46 | -1.37 | -1.42 | 0.06 |
| *tpiA* | 2771.52 | 2856.06 | 1950.07 | 2013.53 | -1.42 | -1.42 | -1.42 | 0.00 |
| *SAUSA300_1272* | 323.30 | 290.09 | 206.19 | 227.72 | -1.57 | -1.27 | -1.42 | 0.21 |
| *plc* | 5330.32 | 5883.53 | 4164.98 | 3764.19 | -1.28 | -1.56 | -1.42 | 0.20 |
| *SAUSA300_1693* | 289.65 | 251.41 | 214.43 | 168.11 | -1.35 | -1.50 | -1.42 | 0.10 |
| *SAUSA300_0211* | 257.20 | 262.84 | 189.69 | 174.23 | -1.36 | -1.51 | -1.43 | 0.11 |
| *fruB* | 235.57 | 229.43 | 175.03 | 149.01 | -1.35 | -1.54 | -1.44 | 0.14 |
| *SAUSA300_0693* | 437.48 | 374.48 | 297.83 | 263.63 | -1.47 | -1.42 | -1.44 | 0.03 |
| *folA* | 638.20 | 516.89 | 414.21 | 382.84 | -1.54 | -1.35 | -1.45 | 0.13 |
| *SAUSA300_0535* | 1245.14 | 1198.15 | 863.24 | 825.28 | -1.44 | -1.45 | -1.45 | 0.01 |
| *glk* | 687.47 | 741.92 | 517.76 | 473.01 | -1.33 | -1.57 | -1.45 | 0.17 |
| *SAUSA300_0353* | 435.08 | 367.45 | 264.84 | 290.38 | -1.64 | -1.27 | -1.45 | 0.27 |
| *SAUSA300_2273* | 420.66 | 409.64 | 268.50 | 304.90 | -1.57 | -1.34 | -1.46 | 0.16 |
| *qoxB* | 82810.39 | 80902.24 | 56177.24 | 56028.09 | -1.47 | -1.44 | -1.46 | 0.02 |
| *gudB* | 3186.17 | 2912.32 | 2139.76 | 2035.69 | -1.49 | -1.43 | -1.46 | 0.04 |
| *SAUSA300_0104* | 374.98 | 383.27 | 267.58 | 252.17 | -1.40 | -1.52 | -1.46 | 0.08 |
| *SAUSA300_0884* | 329.31 | 225.04 | 212.60 | 163.53 | -1.55 | -1.38 | -1.46 | 0.12 |
| *SAUSA300_0175* | 350.95 | 344.59 | 215.35 | 265.92 | -1.63 | -1.30 | -1.46 | 0.24 |
| *rplF* | 3737.83 | 3518.86 | 2434.84 | 2510.23 | -1.54 | -1.40 | -1.47 | 0.09 |
| *rexB* | 1026.40 | 1181.45 | 752.35 | 751.16 | -1.36 | -1.57 | -1.47 | 0.15 |
| *ipk* | 2281.16 | 2398.95 | 1525.78 | 1663.55 | -1.50 | -1.44 | -1.47 | 0.04 |
| *SAUSA300_2309* | 171.87 | 184.60 | 120.96 | 121.50 | -1.42 | -1.52 | -1.47 | 0.07 |
| *dnaI* | 1753.54 | 1725.59 | 1208.71 | 1157.68 | -1.45 | -1.49 | -1.47 | 0.03 |
| *rpsH* | 2562.40 | 2284.67 | 1676.07 | 1616.94 | -1.53 | -1.41 | -1.47 | 0.08 |
| *SAUSA300_1899* | 698.29 | 651.38 | 445.36 | 470.72 | -1.57 | -1.38 | -1.48 | 0.13 |
| *SAUSA300_0250* | 843.72 | 922.13 | 558.08 | 640.36 | -1.51 | -1.44 | -1.48 | 0.05 |
| *estA* | 328.11 | 279.54 | 190.61 | 226.19 | -1.72 | -1.24 | -1.48 | 0.34 |
| *SAUSA300_1064* | 2247.51 | 1995.46 | 1377.33 | 1504.61 | -1.63 | -1.33 | -1.48 | 0.22 |
| *polA* | 1431.43 | 1526.04 | 978.70 | 1010.97 | -1.46 | -1.51 | -1.49 | 0.03 |
| *SAUSA300_1112* | 262.01 | 259.32 | 187.86 | 164.29 | -1.39 | -1.58 | -1.49 | 0.13 |
| *SAUSA300_2389* | 533.63 | 646.11 | 367.47 | 421.81 | -1.45 | -1.53 | -1.49 | 0.06 |
| *SAUSA300_2331* | 1061.26 | 970.48 | 690.04 | 670.92 | -1.54 | -1.45 | -1.49 | 0.06 |
| *SAUSA300_1224* | 237.97 | 254.93 | 173.20 | 158.18 | -1.37 | -1.61 | -1.49 | 0.17 |
| *uvrB* | 588.92 | 628.53 | 405.04 | 408.82 | -1.45 | -1.54 | -1.50 | 0.06 |
| *gpmI* | 6171.63 | 6446.12 | 4203.46 | 4206.63 | -1.47 | -1.53 | -1.50 | 0.05 |
| *rplR* | 2828.01 | 2873.64 | 1981.23 | 1817.14 | -1.43 | -1.58 | -1.50 | 0.11 |
| *SAUSA300_1623* | 179.08 | 177.57 | 111.80 | 126.08 | -1.60 | -1.41 | -1.51 | 0.14 |
| *ezrA* | 6319.46 | 5659.37 | 4033.93 | 3911.67 | -1.57 | -1.45 | -1.51 | 0.08 |
| *SAUSA300_1787* | 761.99 | 747.20 | 520.51 | 480.65 | -1.46 | -1.55 | -1.51 | 0.06 |
| *atpG* | 9017.67 | 8794.09 | 6184.69 | 5631.01 | -1.46 | -1.56 | -1.51 | 0.07 |
| *cdd* | 218.74 | 188.12 | 127.38 | 144.42 | -1.72 | -1.30 | -1.51 | 0.29 |
| *SAUSA300_0236* | 421.86 | 418.43 | 278.58 | 275.86 | -1.51 | -1.52 | -1.52 | 0.00 |
| *mfd* | 1014.38 | 1128.71 | 720.28 | 693.08 | -1.41 | -1.63 | -1.52 | 0.16 |
| *arcR* | 543.25 | 551.17 | 327.15 | 399.65 | -1.66 | -1.38 | -1.52 | 0.20 |
| *SAUSA300_0833* | 442.29 | 385.91 | 268.50 | 276.62 | -1.65 | -1.40 | -1.52 | 0.18 |
| *recQ* | 1610.51 | 1584.94 | 1104.25 | 995.68 | -1.46 | -1.59 | -1.53 | 0.09 |
| *SAUSA300_0753* | 425.46 | 469.42 | 278.58 | 306.42 | -1.53 | -1.53 | -1.53 | 0.00 |
| *rpiA* | 374.98 | 319.10 | 261.17 | 196.39 | -1.44 | -1.62 | -1.53 | 0.13 |
| *SAUSA300_2517* | 203.12 | 173.17 | 116.38 | 131.43 | -1.75 | -1.32 | -1.53 | 0.30 |
| *SAUSA300_0834* | 1245.14 | 1286.06 | 822.92 | 829.10 | -1.51 | -1.55 | -1.53 | 0.03 |
| *SAUSA300_2261* | 600.94 | 580.18 | 413.29 | 359.91 | -1.45 | -1.61 | -1.53 | 0.11 |
| *SAUSA300_1509* | 549.26 | 607.43 | 372.97 | 379.78 | -1.47 | -1.60 | -1.54 | 0.09 |
| *SAUSA300_2026* | 1953.05 | 2102.70 | 1336.09 | 1305.16 | -1.46 | -1.61 | -1.54 | 0.11 |
| *SAUSA300_2384* | 737.95 | 803.46 | 517.76 | 481.41 | -1.43 | -1.67 | -1.55 | 0.17 |
| *SAUSA300_1859* | 343.74 | 334.92 | 203.44 | 237.65 | -1.69 | -1.41 | -1.55 | 0.20 |
| *SAUSA300_2130* | 2043.19 | 1942.72 | 1287.52 | 1283.77 | -1.59 | -1.51 | -1.55 | 0.05 |
| *SAUSA300_1852* | 307.68 | 277.78 | 163.12 | 227.72 | -1.89 | -1.22 | -1.55 | 0.47 |
| *ahpF* | 3864.03 | 3954.00 | 2686.84 | 2370.39 | -1.44 | -1.67 | -1.55 | 0.16 |
| *rpsP* | 1703.06 | 1889.97 | 1204.13 | 1116.42 | -1.41 | -1.69 | -1.55 | 0.20 |
| *SAUSA300_0219* | 147.83 | 141.53 | 88.89 | 97.81 | -1.66 | -1.45 | -1.56 | 0.15 |
| *SAUSA300_0486* | 2116.50 | 2129.95 | 1372.75 | 1352.54 | -1.54 | -1.57 | -1.56 | 0.02 |
| *fabZ* | 442.29 | 481.72 | 271.25 | 322.47 | -1.63 | -1.49 | -1.56 | 0.10 |
| *SAUSA300_1536* | 524.02 | 566.11 | 335.40 | 360.68 | -1.56 | -1.57 | -1.57 | 0.01 |
| *SAUSA300_0631* | 2050.40 | 2179.18 | 1338.84 | 1344.90 | -1.53 | -1.62 | -1.58 | 0.06 |
| *SAUSA300_1261* | 247.59 | 200.42 | 150.29 | 132.96 | -1.65 | -1.51 | -1.58 | 0.10 |
| *fhuB* | 133.41 | 130.10 | 86.14 | 81.00 | -1.55 | -1.61 | -1.58 | 0.04 |
| *SAUSA300_0441* | 318.50 | 264.60 | 175.95 | 196.39 | -1.81 | -1.35 | -1.58 | 0.33 |
| *deoB* | 2297.98 | 2077.21 | 1413.07 | 1350.25 | -1.63 | -1.54 | -1.58 | 0.06 |
| *SAUSA300_1318* | 697.09 | 758.63 | 445.36 | 472.24 | -1.57 | -1.61 | -1.59 | 0.03 |
| *SAUSA300_0383* | 895.40 | 912.46 | 568.16 | 571.58 | -1.58 | -1.60 | -1.59 | 0.01 |
| *galU* | 325.71 | 381.51 | 240.09 | 209.38 | -1.36 | -1.82 | -1.59 | 0.33 |
| *SAUSA300_1971* | 174.27 | 184.60 | 111.80 | 113.09 | -1.56 | -1.63 | -1.60 | 0.05 |
| *SAUSA300_2584* | 1200.67 | 1247.38 | 730.36 | 801.59 | -1.64 | -1.56 | -1.60 | 0.06 |
| *SAUSA300_0218* | 260.81 | 263.72 | 165.87 | 162.00 | -1.57 | -1.63 | -1.60 | 0.04 |
| *qoxA* | 50854.93 | 52274.88 | 32397.01 | 32005.57 | -1.57 | -1.63 | -1.60 | 0.04 |
| *speG* | 227.15 | 228.55 | 129.21 | 157.41 | -1.76 | -1.45 | -1.60 | 0.22 |
| *arcC* | 3305.16 | 2867.48 | 1844.69 | 2009.71 | -1.79 | -1.43 | -1.61 | 0.26 |
| *SAUSA300_1303* | 554.06 | 465.90 | 333.56 | 298.78 | -1.66 | -1.56 | -1.61 | 0.07 |
| *recJ* | 1380.95 | 1335.29 | 827.50 | 856.61 | -1.67 | -1.56 | -1.61 | 0.08 |
| *thyA* | 301.67 | 348.11 | 232.76 | 179.57 | -1.30 | -1.94 | -1.62 | 0.45 |
| *sarA* | 2602.06 | 2362.90 | 1753.05 | 1344.14 | -1.48 | -1.76 | -1.62 | 0.19 |
| *xpt* | 92.54 | 91.42 | 54.98 | 57.31 | -1.68 | -1.60 | -1.64 | 0.06 |
| *ndk* | 341.33 | 359.53 | 210.77 | 216.25 | -1.62 | -1.66 | -1.64 | 0.03 |
| *nrdF* | 5784.62 | 5744.64 | 3529.00 | 3471.52 | -1.64 | -1.65 | -1.65 | 0.01 |
| *SAUSA300_2542* | 2075.64 | 2051.72 | 1247.20 | 1257.02 | -1.66 | -1.63 | -1.65 | 0.02 |
| *ftsK* | 2374.90 | 2407.74 | 1516.62 | 1381.58 | -1.57 | -1.74 | -1.65 | 0.13 |
| *SAUSA300_2379* | 603.34 | 552.93 | 358.31 | 338.52 | -1.68 | -1.63 | -1.66 | 0.04 |
| *SAUSA300_2076* | 2908.54 | 2746.17 | 1696.23 | 1700.99 | -1.71 | -1.61 | -1.66 | 0.07 |
| *SAUSA300_0645* | 143.02 | 118.67 | 83.39 | 73.36 | -1.72 | -1.62 | -1.67 | 0.07 |
| *brnQ* | 1281.20 | 1261.45 | 822.92 | 706.07 | -1.56 | -1.79 | -1.67 | 0.16 |
| *SAUSA300_1012* | 556.47 | 537.10 | 339.98 | 314.06 | -1.64 | -1.71 | -1.67 | 0.05 |
| *SAUSA300_0177* | 954.29 | 989.82 | 583.74 | 576.93 | -1.63 | -1.72 | -1.68 | 0.06 |
| *topB* | 598.53 | 566.99 | 340.90 | 354.56 | -1.76 | -1.60 | -1.68 | 0.11 |
| *pbuX* | 223.55 | 231.19 | 123.71 | 149.01 | -1.81 | -1.55 | -1.68 | 0.18 |
| *SAUSA300_1695* | 2719.84 | 2667.06 | 1587.18 | 1613.88 | -1.71 | -1.65 | -1.68 | 0.04 |
| *SAUSA300_0761* | 491.57 | 463.26 | 276.75 | 285.03 | -1.78 | -1.63 | -1.70 | 0.11 |
| *arlR* | 192.30 | 238.22 | 109.97 | 143.66 | -1.75 | -1.66 | -1.70 | 0.06 |
| *SAUSA300_1930* | 49.28 | 65.93 | 32.99 | 34.39 | -1.49 | -1.92 | -1.71 | 0.30 |
| *ccpA* | 1418.21 | 1323.86 | 827.50 | 777.90 | -1.71 | -1.70 | -1.71 | 0.01 |
| *ispD* | 296.86 | 290.09 | 156.70 | 190.27 | -1.89 | -1.52 | -1.71 | 0.26 |
| *SAUSA300_1434* | 110.57 | 116.04 | 65.98 | 65.72 | -1.68 | -1.77 | -1.72 | 0.06 |
| *fumC* | 1100.92 | 960.81 | 581.91 | 613.61 | -1.89 | -1.57 | -1.73 | 0.23 |
| *SAUSA300_2363* | 260.81 | 220.64 | 140.21 | 136.02 | -1.86 | -1.62 | -1.74 | 0.17 |
| *murG* | 233.16 | 217.13 | 109.97 | 158.94 | -2.12 | -1.37 | -1.74 | 0.53 |
| *SAUSA300_1183* | 646.61 | 698.85 | 385.80 | 382.07 | -1.68 | -1.83 | -1.75 | 0.11 |
| *rarD* | 111.77 | 125.71 | 69.65 | 65.72 | -1.60 | -1.91 | -1.76 | 0.22 |
| *SAUSA300_1050* | 524.02 | 494.91 | 280.41 | 299.55 | -1.87 | -1.65 | -1.76 | 0.15 |
| *SAUSA300_1068* | 811.27 | 912.46 | 487.52 | 491.35 | -1.66 | -1.86 | -1.76 | 0.14 |
| *purR* | 2496.29 | 2378.73 | 1408.49 | 1358.65 | -1.77 | -1.75 | -1.76 | 0.02 |
| *secG* | 639.40 | 687.42 | 419.70 | 340.81 | -1.52 | -2.02 | -1.77 | 0.35 |
| *dgkA* | 143.02 | 108.12 | 62.31 | 86.35 | -2.30 | -1.25 | -1.77 | 0.74 |
| *SAUSA300_1453* | 239.17 | 278.66 | 133.79 | 158.18 | -1.79 | -1.76 | -1.77 | 0.02 |
| *SAUSA300_2639* | 36861.50 | 36646.12 | 22250.78 | 19346.70 | -1.66 | -1.89 | -1.78 | 0.17 |
| *SAUSA300_0981* | 394.21 | 329.65 | 213.52 | 193.33 | -1.85 | -1.71 | -1.78 | 0.10 |
| *SAUSA300_2209* | 133.41 | 98.45 | 65.98 | 64.19 | -2.02 | -1.53 | -1.78 | 0.35 |
| *dat* | 1224.71 | 1169.15 | 690.04 | 656.40 | -1.77 | -1.78 | -1.78 | 0.00 |
| *SAUSA300_2461* | 155.04 | 167.90 | 92.56 | 88.64 | -1.68 | -1.89 | -1.78 | 0.15 |
| *rocD* | 1057.65 | 1013.55 | 623.14 | 537.96 | -1.70 | -1.88 | -1.79 | 0.13 |
| *SAUSA300_1914* | 296.86 | 282.18 | 144.79 | 184.16 | -2.05 | -1.53 | -1.79 | 0.37 |
| *uvrC* | 492.77 | 428.98 | 257.50 | 256.75 | -1.91 | -1.67 | -1.79 | 0.17 |
| *ybaK* | 206.72 | 164.38 | 110.88 | 95.52 | -1.86 | -1.72 | -1.79 | 0.10 |
| *hemA* | 6794.20 | 6396.02 | 3630.72 | 3722.93 | -1.87 | -1.72 | -1.79 | 0.11 |
| *spxA* | 34219.78 | 34843.18 | 21296.82 | 17560.88 | -1.61 | -1.98 | -1.80 | 0.27 |
| *SAUSA300_1353* | 211.53 | 220.64 | 122.80 | 117.68 | -1.72 | -1.87 | -1.80 | 0.11 |
| *mgtE* | 353.35 | 371.84 | 209.85 | 193.33 | -1.68 | -1.92 | -1.80 | 0.17 |
| *phoR* | 974.72 | 890.48 | 509.51 | 516.56 | -1.91 | -1.72 | -1.82 | 0.13 |
| *SAUSA300_2251* | 263.21 | 265.48 | 149.37 | 141.37 | -1.76 | -1.88 | -1.82 | 0.08 |
| *SAUSA300_0646* | 319.70 | 312.94 | 178.70 | 168.88 | -1.79 | -1.85 | -1.82 | 0.05 |
| *SAUSA300_1998* | 305.28 | 291.85 | 157.62 | 170.40 | -1.94 | -1.71 | -1.82 | 0.16 |
| *lctP* | 676.66 | 650.50 | 338.15 | 393.54 | -2.00 | -1.65 | -1.83 | 0.25 |
| *SAUSA300_0642* | 129.80 | 155.59 | 72.39 | 83.29 | -1.79 | -1.87 | -1.83 | 0.05 |
| *SAUSA300_1051* | 830.50 | 774.45 | 413.29 | 465.37 | -2.01 | -1.66 | -1.84 | 0.24 |
| *hsdM* | 124.99 | 125.71 | 60.48 | 77.94 | -2.07 | -1.61 | -1.84 | 0.32 |
| *SAUSA300_0716* | 9885.42 | 9968.50 | 5311.38 | 5416.28 | -1.86 | -1.84 | -1.85 | 0.01 |
| *SAUSA300_1107* | 1886.94 | 1519.01 | 1056.59 | 789.36 | -1.79 | -1.92 | -1.86 | 0.10 |
| *rbgA* | 82.93 | 68.57 | 45.82 | 35.91 | -1.81 | -1.91 | -1.86 | 0.07 |
| *frp* | 159.85 | 162.63 | 88.89 | 84.06 | -1.80 | -1.93 | -1.87 | 0.10 |
| *SAUSA300_0666* | 753.58 | 726.10 | 374.80 | 417.99 | -2.01 | -1.74 | -1.87 | 0.19 |
| *SAUSA300_0704* | 629.78 | 641.71 | 317.99 | 362.97 | -1.98 | -1.77 | -1.87 | 0.15 |
| *ilvE* | 1554.02 | 1598.13 | 783.51 | 904.75 | -1.98 | -1.77 | -1.87 | 0.15 |
| *SAUSA300_1182* | 789.63 | 870.27 | 439.87 | 443.21 | -1.80 | -1.96 | -1.88 | 0.12 |
| *SAUSA300_1067* | 1575.66 | 1749.32 | 921.88 | 852.02 | -1.71 | -2.05 | -1.88 | 0.24 |
| *SAUSA300_2146* | 306.48 | 260.20 | 141.12 | 162.76 | -2.17 | -1.60 | -1.89 | 0.41 |
| *SAUSA300_0658* | 884.58 | 788.51 | 447.20 | 438.62 | -1.98 | -1.80 | -1.89 | 0.13 |
| *hutH* | 144.22 | 128.34 | 72.39 | 71.83 | -1.99 | -1.79 | -1.89 | 0.15 |
| *SAUSA300_0679* | 117.78 | 116.04 | 66.90 | 57.31 | -1.76 | -2.02 | -1.89 | 0.19 |
| *glvC* | 320.90 | 216.25 | 151.20 | 129.14 | -2.12 | -1.67 | -1.90 | 0.32 |
| *SAUSA300_0537* | 176.68 | 160.87 | 87.06 | 90.93 | -2.03 | -1.77 | -1.90 | 0.18 |
| *SAUSA300_2401* | 118.99 | 128.34 | 87.06 | 52.73 | -1.37 | -2.43 | -1.90 | 0.75 |
| *SAUSA300_0279* | 156.24 | 170.54 | 100.80 | 75.65 | -1.55 | -2.25 | -1.90 | 0.50 |
| *SAUSA300_1863* | 1152.60 | 1009.16 | 541.58 | 601.38 | -2.13 | -1.68 | -1.90 | 0.32 |
| *SAUSA300_2456* | 139.42 | 150.32 | 70.56 | 81.76 | -1.98 | -1.84 | -1.91 | 0.10 |
| *SAUSA300_0651* | 1411.00 | 1281.66 | 693.70 | 719.06 | -2.03 | -1.78 | -1.91 | 0.18 |
| *SAUSA300_0406* | 84.13 | 74.72 | 41.24 | 42.03 | -2.04 | -1.78 | -1.91 | 0.19 |
| *SAUSA300_0689* | 740.35 | 670.72 | 349.14 | 392.77 | -2.12 | -1.71 | -1.91 | 0.29 |
| *SAUSA300_1842* | 6331.48 | 6064.61 | 3740.69 | 2838.81 | -1.69 | -2.14 | -1.91 | 0.31 |
| *SAUSA300_2137* | 231.96 | 225.04 | 109.05 | 132.20 | -2.13 | -1.70 | -1.91 | 0.30 |
| *SAUSA300_2435* | 216.34 | 189.88 | 100.80 | 112.33 | -2.15 | -1.69 | -1.92 | 0.32 |
| *SAUSA300_1631* | 1679.02 | 1587.58 | 864.15 | 831.39 | -1.94 | -1.91 | -1.93 | 0.02 |
| *truB* | 133.41 | 103.73 | 62.31 | 60.37 | -2.14 | -1.72 | -1.93 | 0.30 |
| *SAUSA300_2581* | 539.64 | 523.04 | 308.82 | 247.58 | -1.75 | -2.11 | -1.93 | 0.26 |
| *pknB* | 1592.48 | 1675.48 | 843.08 | 836.74 | -1.89 | -2.00 | -1.95 | 0.08 |
| *SAUSA300_1354* | 1592.48 | 1498.79 | 790.84 | 796.24 | -2.01 | -1.88 | -1.95 | 0.09 |
| *SAUSA300_0812* | 138.22 | 131.86 | 76.98 | 62.66 | -1.80 | -2.10 | -1.95 | 0.22 |
| *SAUSA300_2169* | 558.87 | 595.12 | 271.25 | 321.71 | -2.06 | -1.85 | -1.96 | 0.15 |
| *cbf1* | 2081.65 | 2191.49 | 1170.23 | 1027.78 | -1.78 | -2.13 | -1.96 | 0.25 |
| *SAUSA300_1437* | 947.08 | 937.07 | 478.35 | 479.12 | -1.98 | -1.96 | -1.97 | 0.02 |
| *SAUSA300_1194* | 87.74 | 73.84 | 42.15 | 39.74 | -2.08 | -1.86 | -1.97 | 0.16 |
| *SAUSA300_1333* | 879.77 | 853.56 | 473.77 | 406.53 | -1.86 | -2.10 | -1.98 | 0.17 |
| *SAUSA300_0195* | 1247.55 | 987.18 | 579.16 | 547.13 | -2.15 | -1.80 | -1.98 | 0.25 |
| *SAUSA300_1967* | 40.86 | 46.59 | 21.99 | 22.16 | -1.86 | -2.10 | -1.98 | 0.17 |
| *SAUSA300_2576* | 507.19 | 429.86 | 214.43 | 266.69 | -2.37 | -1.61 | -1.99 | 0.53 |
| *SAUSA300_0171* | 99.76 | 114.28 | 46.74 | 60.37 | -2.13 | -1.89 | -2.01 | 0.17 |
| *SAUSA300_2136* | 330.52 | 301.52 | 156.70 | 155.89 | -2.11 | -1.93 | -2.02 | 0.12 |
| *SAUSA300_0346* | 60.09 | 53.62 | 30.24 | 25.98 | -1.99 | -2.06 | -2.03 | 0.05 |
| *SAUSA300_2376* | 54.08 | 64.17 | 31.16 | 27.51 | -1.74 | -2.33 | -2.03 | 0.42 |
| *SAUSA300_2548* | 61.30 | 70.32 | 27.49 | 38.21 | -2.23 | -1.84 | -2.04 | 0.28 |
| *SAUSA300_0382* | 2392.93 | 2322.47 | 1079.50 | 1250.91 | -2.22 | -1.86 | -2.04 | 0.25 |
| *SAUSA300_2218* | 575.70 | 675.12 | 325.32 | 291.90 | -1.77 | -2.31 | -2.04 | 0.38 |
| *SAUSA300_0451* | 117.78 | 114.28 | 61.40 | 52.73 | -1.92 | -2.17 | -2.04 | 0.18 |
| *SAUSA300_0385* | 2056.41 | 1979.64 | 1110.66 | 883.35 | -1.85 | -2.24 | -2.05 | 0.28 |
| *SAUSA300_0814* | 344.94 | 353.38 | 178.70 | 162.76 | -1.93 | -2.17 | -2.05 | 0.17 |
| *proC* | 306.48 | 367.45 | 162.20 | 165.82 | -1.89 | -2.22 | -2.05 | 0.23 |
| *phnE* | 131.00 | 121.31 | 55.90 | 68.01 | -2.34 | -1.78 | -2.06 | 0.40 |
| *norA* | 241.58 | 297.12 | 115.46 | 145.19 | -2.09 | -2.05 | -2.07 | 0.03 |
| *sucA* | 11058.45 | 9361.96 | 4845.85 | 5031.91 | -2.28 | -1.86 | -2.07 | 0.30 |
| *SAUSA300_1984* | 217.54 | 216.25 | 115.46 | 94.75 | -1.88 | -2.28 | -2.08 | 0.28 |
| *opuCd* | 1985.50 | 1991.94 | 982.37 | 926.91 | -2.02 | -2.15 | -2.09 | 0.09 |
| *SAUSA300_0197* | 371.38 | 350.74 | 187.86 | 159.71 | -1.98 | -2.20 | -2.09 | 0.16 |
| *hemX* | 646.61 | 748.08 | 346.39 | 323.23 | -1.87 | -2.31 | -2.09 | 0.32 |
| *SAUSA300_2131* | 670.65 | 667.20 | 370.22 | 281.21 | -1.81 | -2.37 | -2.09 | 0.40 |
| *SAUSA300_2099* | 1816.03 | 1933.93 | 801.84 | 1005.62 | -2.26 | -1.92 | -2.09 | 0.24 |
| *SAUSA300_2254* | 2989.06 | 2390.16 | 1350.75 | 1205.83 | -2.21 | -1.98 | -2.10 | 0.16 |
| *tmk* | 114.18 | 147.68 | 57.73 | 66.48 | -1.98 | -2.22 | -2.10 | 0.17 |
| *SAUSA300_2355* | 68.51 | 58.90 | 25.66 | 38.21 | -2.67 | -1.54 | -2.11 | 0.80 |
| *SAUSA300_0602* | 18562.96 | 16387.38 | 8801.89 | 7720.19 | -2.11 | -2.12 | -2.12 | 0.01 |
| *putP* | 1076.88 | 952.90 | 459.11 | 504.34 | -2.35 | -1.89 | -2.12 | 0.32 |
| *SAUSA300_0246* | 365.37 | 409.64 | 167.70 | 198.68 | -2.18 | -2.06 | -2.12 | 0.08 |
| *SAUSA300_1054* | 206.72 | 158.23 | 93.47 | 77.18 | -2.21 | -2.05 | -2.13 | 0.11 |
| *agrD* | 4504.63 | 4627.36 | 2301.96 | 2007.42 | -1.96 | -2.31 | -2.13 | 0.25 |
| *glpF* | 1104.52 | 1177.94 | 574.57 | 502.81 | -1.92 | -2.34 | -2.13 | 0.30 |
| *SAUSA300_1352* | 100.96 | 145.04 | 54.98 | 59.60 | -1.84 | -2.43 | -2.13 | 0.42 |
| *SAUSA300_0209* | 98.55 | 116.91 | 45.82 | 55.02 | -2.15 | -2.12 | -2.14 | 0.02 |
| *lytN* | 58.89 | 57.14 | 36.66 | 21.40 | -1.61 | -2.67 | -2.14 | 0.75 |
| *SAUSA300_1898* | 117.78 | 141.53 | 65.06 | 57.31 | -1.81 | -2.47 | -2.14 | 0.47 |
| *SAUSA300_1530* | 233.16 | 289.21 | 136.54 | 111.57 | -1.71 | -2.59 | -2.15 | 0.63 |
| *glpD* | 9713.55 | 9820.82 | 4556.27 | 4476.38 | -2.13 | -2.19 | -2.16 | 0.04 |
| *opuCa* | 874.96 | 879.94 | 372.97 | 443.97 | -2.35 | -1.98 | -2.16 | 0.26 |
| *SAUSA300_2383* | 292.06 | 283.06 | 117.30 | 153.59 | -2.49 | -1.84 | -2.17 | 0.46 |
| *SAUSA300_0352* | 300.47 | 272.51 | 137.46 | 126.85 | -2.19 | -2.15 | -2.17 | 0.03 |
| *SAUSA300_0421* | 39.66 | 38.68 | 21.99 | 15.28 | -1.80 | -2.53 | -2.17 | 0.51 |
| *opuCb* | 677.86 | 679.51 | 331.73 | 296.49 | -2.04 | -2.29 | -2.17 | 0.18 |
| *agrB* | 19946.32 | 18704.57 | 9377.38 | 8461.41 | -2.13 | -2.21 | -2.17 | 0.06 |
| *arcD* | 528.82 | 666.33 | 239.18 | 311.77 | -2.21 | -2.14 | -2.17 | 0.05 |
| *treP* | 13360.04 | 13402.98 | 6085.72 | 6161.32 | -2.20 | -2.18 | -2.19 | 0.01 |
| *SAUSA300_2624* | 274.03 | 278.66 | 130.13 | 123.03 | -2.11 | -2.27 | -2.19 | 0.11 |
| *hemC* | 1271.58 | 1107.61 | 506.76 | 589.16 | -2.51 | -1.88 | -2.19 | 0.44 |
| *hsdR* | 1120.15 | 1095.30 | 496.68 | 512.74 | -2.26 | -2.14 | -2.20 | 0.08 |
| *pckA* | 3082.81 | 2709.25 | 1352.59 | 1273.07 | -2.28 | -2.13 | -2.20 | 0.11 |
| *SAUSA300_0598* | 149.03 | 144.17 | 55.90 | 82.53 | -2.67 | -1.75 | -2.21 | 0.65 |
| *SAUSA300_1910* | 483.15 | 505.46 | 228.18 | 219.31 | -2.12 | -2.30 | -2.21 | 0.13 |
| *ftsL* | 368.98 | 413.16 | 183.28 | 171.17 | -2.01 | -2.41 | -2.21 | 0.28 |
| *SAUSA300_0028* | 86.53 | 69.45 | 31.16 | 42.03 | -2.78 | -1.65 | -2.21 | 0.80 |
| *SAUSA300_0384* | 652.62 | 637.32 | 303.32 | 279.68 | -2.15 | -2.28 | -2.22 | 0.09 |
| *SAUSA300_0551* | 1120.15 | 1042.56 | 498.51 | 476.06 | -2.25 | -2.19 | -2.22 | 0.04 |
| *trmB* | 2872.48 | 2664.42 | 1276.53 | 1214.23 | -2.25 | -2.19 | -2.22 | 0.04 |
| *SAUSA300_0265* | 106.97 | 159.11 | 50.40 | 68.01 | -2.12 | -2.34 | -2.23 | 0.15 |
| *sucB* | 4811.11 | 3840.60 | 1956.49 | 1915.72 | -2.46 | -2.00 | -2.23 | 0.32 |
| *SAUSA300_2351* | 417.05 | 348.11 | 189.69 | 153.59 | -2.20 | -2.27 | -2.23 | 0.05 |
| *phoH* | 356.96 | 455.35 | 192.44 | 174.23 | -1.85 | -2.61 | -2.23 | 0.54 |
| *hsdS* | 175.47 | 153.83 | 76.06 | 71.07 | -2.31 | -2.16 | -2.24 | 0.10 |
| *opuCc* | 1804.01 | 1911.07 | 842.16 | 819.93 | -2.14 | -2.33 | -2.24 | 0.13 |
| *SAUSA300_0098* | 149.03 | 181.09 | 75.14 | 72.59 | -1.98 | -2.49 | -2.24 | 0.36 |
| *SAUSA300_0552* | 521.61 | 501.06 | 219.93 | 236.89 | -2.37 | -2.12 | -2.24 | 0.18 |
| *SAUSA300_0632* | 628.58 | 601.27 | 260.25 | 289.61 | -2.42 | -2.08 | -2.25 | 0.24 |
| *SAUSA300_2101* | 42.07 | 41.32 | 17.41 | 19.87 | -2.42 | -2.08 | -2.25 | 0.24 |
| *SAUSA300_1864* | 507.19 | 546.77 | 194.27 | 289.61 | -2.61 | -1.89 | -2.25 | 0.51 |
| *SAUSA300_1904* | 747.57 | 773.57 | 388.55 | 299.55 | -1.92 | -2.58 | -2.25 | 0.47 |
| *sgtB* | 842.51 | 705.88 | 370.22 | 310.24 | -2.28 | -2.28 | -2.28 | 0.00 |
| *SAUSA300_1716* | 43.27 | 49.23 | 26.58 | 16.81 | -1.63 | -2.93 | -2.28 | 0.92 |
| *lysA* | 394.21 | 347.23 | 154.87 | 169.64 | -2.55 | -2.05 | -2.30 | 0.35 |
| *thiE* | 81.73 | 70.32 | 33.91 | 32.09 | -2.41 | -2.19 | -2.30 | 0.15 |
| *est* | 1117.74 | 1140.14 | 507.68 | 474.54 | -2.20 | -2.40 | -2.30 | 0.14 |
| *SAUSA300_0097* | 352.15 | 378.87 | 144.79 | 173.46 | -2.43 | -2.18 | -2.31 | 0.18 |
| *arcB* | 431.47 | 465.90 | 190.61 | 197.91 | -2.26 | -2.35 | -2.31 | 0.06 |
| *SAUSA300_1698* | 1176.64 | 1115.52 | 493.02 | 498.22 | -2.39 | -2.24 | -2.31 | 0.10 |
| *argS* | 1658.59 | 1597.25 | 701.95 | 705.31 | -2.36 | -2.26 | -2.31 | 0.07 |
| *SAUSA300_1913* | 551.66 | 520.40 | 224.51 | 239.18 | -2.46 | -2.18 | -2.32 | 0.20 |
| *treR* | 2840.03 | 3166.36 | 1244.45 | 1345.66 | -2.28 | -2.35 | -2.32 | 0.05 |
| *SAUSA300_1980* | 173.07 | 165.26 | 90.72 | 60.37 | -1.91 | -2.74 | -2.32 | 0.59 |
| *SAUSA300_0055* | 182.68 | 124.83 | 77.89 | 54.25 | -2.35 | -2.30 | -2.32 | 0.03 |
| *SAUSA300_0678* | 149.03 | 158.23 | 57.73 | 76.41 | -2.58 | -2.07 | -2.33 | 0.36 |
| *SAUSA300_0125* | 48.07 | 65.93 | 20.16 | 29.04 | -2.38 | -2.27 | -2.33 | 0.08 |
| *SAUSA300_0139* | 659.83 | 559.08 | 250.17 | 275.86 | -2.64 | -2.03 | -2.33 | 0.43 |
| *SAUSA300_1560* | 56.49 | 64.17 | 30.24 | 22.92 | -1.87 | -2.80 | -2.33 | 0.66 |
| *SAUSA300_0419* | 34.85 | 37.80 | 13.75 | 17.58 | -2.54 | -2.15 | -2.34 | 0.27 |
| *agrC* | 24032.69 | 21646.78 | 10230.54 | 9194.99 | -2.35 | -2.35 | -2.35 | 0.00 |
| *SAUSA300_0474* | 4243.82 | 4382.98 | 1924.41 | 1748.37 | -2.21 | -2.51 | -2.36 | 0.21 |
| *rpoF* | 2217.46 | 2184.46 | 927.38 | 940.67 | -2.39 | -2.32 | -2.36 | 0.05 |
| *SAUSA300_1677* | 3108.05 | 2907.04 | 1286.61 | 1253.20 | -2.42 | -2.32 | -2.37 | 0.07 |
| *SAUSA300_0136* | 13100.44 | 10927.56 | 5518.48 | 4608.58 | -2.37 | -2.37 | -2.37 | 0.00 |
| *agrA* | 17446.42 | 14955.39 | 7318.26 | 6330.97 | -2.38 | -2.36 | -2.37 | 0.02 |
| *SAUSA300_2337* | 855.73 | 851.81 | 354.64 | 364.50 | -2.41 | -2.34 | -2.37 | 0.05 |
| *SAUSA300_0550* | 1257.16 | 1257.05 | 531.50 | 524.97 | -2.37 | -2.39 | -2.38 | 0.02 |
| *SAUSA300_1912* | 182.68 | 206.58 | 75.14 | 87.88 | -2.43 | -2.35 | -2.39 | 0.06 |
| *SAUSA300_0652* | 632.19 | 629.40 | 271.25 | 255.23 | -2.33 | -2.47 | -2.40 | 0.10 |
| *SAUSA300_2592* | 352.15 | 397.33 | 155.79 | 156.65 | -2.26 | -2.54 | -2.40 | 0.20 |
| *SAUSA300_1862* | 1167.02 | 1151.56 | 455.44 | 514.27 | -2.56 | -2.24 | -2.40 | 0.23 |
| *mraW* | 1886.94 | 1838.11 | 775.26 | 773.32 | -2.43 | -2.38 | -2.41 | 0.04 |
| *pflA* | 91539.60 | 78638.67 | 35353.27 | 35054.52 | -2.59 | -2.24 | -2.42 | 0.24 |
| *SAUSA300_0146* | 40.86 | 37.80 | 16.49 | 16.05 | -2.48 | -2.36 | -2.42 | 0.09 |
| *rsbU* | 823.28 | 756.87 | 299.66 | 361.44 | -2.75 | -2.09 | -2.42 | 0.46 |
| *efb* | 28.84 | 30.77 | 14.66 | 10.70 | -1.97 | -2.88 | -2.42 | 0.64 |
| *SAUSA300_1788* | 2338.85 | 2259.18 | 1018.11 | 886.41 | -2.30 | -2.55 | -2.42 | 0.18 |
| *guaA* | 4660.87 | 4503.41 | 1899.67 | 1880.57 | -2.45 | -2.39 | -2.42 | 0.04 |
| *potB* | 123.79 | 109.88 | 51.32 | 45.08 | -2.41 | -2.44 | -2.42 | 0.02 |
| *sdrH* | 246.38 | 195.15 | 93.47 | 87.88 | -2.64 | -2.22 | -2.43 | 0.29 |
| *aur* | 394.21 | 366.57 | 141.12 | 177.28 | -2.79 | -2.07 | -2.43 | 0.51 |
| *sirA* | 44.47 | 26.37 | 13.75 | 16.05 | -3.24 | -1.64 | -2.44 | 1.13 |
| *SAUSA300_1230* | 105.76 | 79.99 | 46.74 | 30.57 | -2.26 | -2.62 | -2.44 | 0.25 |
| *murI* | 667.04 | 633.80 | 259.34 | 274.33 | -2.57 | -2.31 | -2.44 | 0.19 |
| *SAUSA300_2213* | 1054.04 | 1083.00 | 456.36 | 416.46 | -2.31 | -2.60 | -2.46 | 0.21 |
| *SAUSA300_0667* | 284.84 | 234.71 | 110.88 | 100.10 | -2.57 | -2.34 | -2.46 | 0.16 |
| *SAUSA300_2321* | 52.88 | 73.84 | 27.49 | 24.45 | -1.92 | -3.02 | -2.47 | 0.78 |
| *SAUSA300_2260* | 425.46 | 405.25 | 170.45 | 165.06 | -2.50 | -2.46 | -2.48 | 0.03 |
| *clpB* | 2263.13 | 2249.51 | 985.12 | 847.44 | -2.30 | -2.65 | -2.48 | 0.25 |
| *SAUSA300_2168* | 424.26 | 331.40 | 149.37 | 155.89 | -2.84 | -2.13 | -2.48 | 0.51 |
| *SAUSA300_2497* | 534.83 | 461.50 | 180.53 | 223.13 | -2.96 | -2.07 | -2.52 | 0.63 |
| *SAUSA300_1005* | 342.53 | 399.97 | 125.54 | 169.64 | -2.73 | -2.36 | -2.54 | 0.26 |
| *nixA* | 128.60 | 119.55 | 62.31 | 38.97 | -2.06 | -3.07 | -2.57 | 0.71 |
| *nrdR* | 722.33 | 675.99 | 269.42 | 275.09 | -2.68 | -2.46 | -2.57 | 0.16 |
| *SAUSA300_0644* | 187.49 | 146.80 | 66.90 | 61.90 | -2.80 | -2.37 | -2.59 | 0.30 |
| *potC* | 140.62 | 140.65 | 44.90 | 68.01 | -3.13 | -2.07 | -2.60 | 0.75 |
| *SAUSA300_1436* | 50.48 | 35.16 | 17.41 | 15.28 | -2.90 | -2.30 | -2.60 | 0.42 |
| *SAUSA300_0192* | 1338.89 | 1190.24 | 515.01 | 456.96 | -2.60 | -2.60 | -2.60 | 0.00 |
| *SAUSA300_0785* | 775.21 | 693.58 | 280.41 | 282.73 | -2.76 | -2.45 | -2.61 | 0.22 |
| *smpB* | 925.44 | 810.49 | 339.06 | 324.76 | -2.73 | -2.50 | -2.61 | 0.17 |
| *argF* | 1650.17 | 1492.64 | 585.57 | 616.67 | -2.82 | -2.42 | -2.62 | 0.28 |
| *SAUSA300_1683* | 1910.98 | 1831.08 | 684.54 | 747.34 | -2.79 | -2.45 | -2.62 | 0.24 |
| *SAUSA300_0194* | 1366.53 | 1065.42 | 449.03 | 474.54 | -3.04 | -2.25 | -2.64 | 0.56 |
| *SAUSA300_0356* | 269.22 | 297.12 | 100.80 | 113.09 | -2.67 | -2.63 | -2.65 | 0.03 |
| *lytH* | 1138.18 | 1257.05 | 482.94 | 424.87 | -2.36 | -2.96 | -2.66 | 0.43 |
| *srrB* | 3015.50 | 3079.34 | 1128.99 | 1153.86 | -2.67 | -2.67 | -2.67 | 0.00 |
| *rnr* | 5468.53 | 5030.84 | 2015.13 | 1908.08 | -2.71 | -2.64 | -2.68 | 0.05 |
| *sufB* | 4773.85 | 4410.23 | 1623.84 | 1811.79 | -2.94 | -2.43 | -2.69 | 0.36 |
| *SAUSA300_2288* | 34.85 | 39.56 | 10.08 | 20.63 | -3.46 | -1.92 | -2.69 | 1.09 |
| *SAUSA300_0475* | 4525.06 | 4615.93 | 1788.79 | 1622.29 | -2.53 | -2.85 | -2.69 | 0.22 |
| *SAUSA300_1792* | 4282.28 | 4265.18 | 1549.61 | 1625.34 | -2.76 | -2.62 | -2.69 | 0.10 |
| *SAUSA300_1214* | 46.87 | 43.07 | 16.49 | 16.81 | -2.84 | -2.56 | -2.70 | 0.20 |
| *sufC* | 1604.50 | 1669.33 | 599.32 | 609.79 | -2.68 | -2.74 | -2.71 | 0.04 |
| *pth* | 108.17 | 77.36 | 30.24 | 42.03 | -3.58 | -1.84 | -2.71 | 1.23 |
| *SAUSA300_1874* | 2364.09 | 2363.78 | 953.04 | 801.59 | -2.48 | -2.95 | -2.71 | 0.33 |
| *nirR* | 200.71 | 176.69 | 72.39 | 66.48 | -2.77 | -2.66 | -2.72 | 0.08 |
| *argJ* | 61.30 | 79.12 | 42.15 | 19.87 | -1.45 | -3.98 | -2.72 | 1.79 |
| *SAUSA300_1685* | 6337.49 | 5949.46 | 2337.70 | 2179.35 | -2.71 | -2.73 | -2.72 | 0.01 |
| *SAUSA300_1944* | 24.04 | 28.13 | 9.16 | 9.93 | -2.62 | -2.83 | -2.73 | 0.15 |
| *pflB* | 269533.68 | 223632.95 | 90347.45 | 90257.33 | -2.98 | -2.48 | -2.73 | 0.36 |
| *SAUSA300_0721* | 48.07 | 35.16 | 14.66 | 16.05 | -3.28 | -2.19 | -2.74 | 0.77 |
| *SAUSA300_2138* | 420.66 | 426.34 | 134.71 | 178.81 | -3.12 | -2.38 | -2.75 | 0.52 |
| *nrdI* | 1718.68 | 1680.76 | 593.82 | 638.83 | -2.89 | -2.63 | -2.76 | 0.19 |
| *rsbW* | 471.13 | 468.54 | 156.70 | 185.69 | -3.01 | -2.52 | -2.76 | 0.34 |
| *SAUSA300_0665* | 33.65 | 47.47 | 10.08 | 20.63 | -3.34 | -2.30 | -2.82 | 0.73 |
| *aroA* | 1177.84 | 1117.28 | 415.12 | 395.83 | -2.84 | -2.82 | -2.83 | 0.01 |
| *SAUSA300_0856* | 57.69 | 86.15 | 28.41 | 23.69 | -2.03 | -3.64 | -2.83 | 1.14 |
| *SAUSA300_1653* | 2301.59 | 2245.11 | 816.50 | 779.43 | -2.82 | -2.88 | -2.85 | 0.04 |
| *SAUSA300_1684* | 2225.87 | 2080.73 | 776.18 | 733.58 | -2.87 | -2.84 | -2.85 | 0.02 |
| *SAUSA300_1916* | 1162.21 | 1073.33 | 440.78 | 349.22 | -2.64 | -3.07 | -2.86 | 0.31 |
| *miaA* | 294.46 | 282.18 | 95.30 | 106.98 | -3.09 | -2.64 | -2.86 | 0.32 |
| *SAUSA300_0681* | 79.32 | 59.78 | 24.74 | 23.69 | -3.21 | -2.52 | -2.86 | 0.48 |
| *SAUSA300_1997* | 99.76 | 109.00 | 33.91 | 38.97 | -2.94 | -2.80 | -2.87 | 0.10 |
| *fhuA* | 752.37 | 710.28 | 274.00 | 236.89 | -2.75 | -3.00 | -2.87 | 0.18 |
| *dtd* | 933.86 | 1033.77 | 357.39 | 330.11 | -2.61 | -3.13 | -2.87 | 0.37 |
| *ald* | 15942.87 | 17088.87 | 5608.28 | 5877.83 | -2.84 | -2.91 | -2.88 | 0.05 |
| *SAUSA300_0458* | 182.68 | 169.66 | 58.65 | 64.19 | -3.11 | -2.64 | -2.88 | 0.33 |
| *SAUSA300_0750* | 1663.39 | 1528.68 | 516.84 | 590.69 | -3.22 | -2.59 | -2.90 | 0.45 |
| *SAUSA300_1793* | 733.14 | 740.17 | 284.08 | 228.48 | -2.58 | -3.24 | -2.91 | 0.47 |
| *SAUSA300_0283* | 152.64 | 130.98 | 40.32 | 64.19 | -3.79 | -2.04 | -2.91 | 1.23 |
| *SAUSA300_0688* | 275.23 | 264.60 | 87.97 | 97.81 | -3.13 | -2.71 | -2.92 | 0.30 |
| *hfq* | 199.51 | 192.51 | 65.98 | 68.01 | -3.02 | -2.83 | -2.93 | 0.14 |
| *SAUSA300_0168* | 85.33 | 59.78 | 31.16 | 19.10 | -2.74 | -3.13 | -2.93 | 0.28 |
| *SAUSA300_0653* | 191.10 | 179.33 | 69.65 | 57.31 | -2.74 | -3.13 | -2.94 | 0.27 |
| *SAUSA300_0657* | 337.73 | 348.11 | 116.38 | 116.15 | -2.90 | -3.00 | -2.95 | 0.07 |
| *SAUSA300_0739* | 37.26 | 21.10 | 9.16 | 11.46 | -4.07 | -1.84 | -2.95 | 1.57 |
| *SAUSA300_0302* | 26.44 | 33.40 | 10.08 | 9.93 | -2.62 | -3.36 | -2.99 | 0.52 |
| *treC* | 7283.36 | 7650.43 | 2392.68 | 2569.07 | -3.04 | -2.98 | -3.01 | 0.05 |
| *purQ* | 22.84 | 32.53 | 6.41 | 12.99 | -3.56 | -2.50 | -3.03 | 0.75 |
| *SAUSA300_0420* | 181.48 | 175.81 | 75.14 | 48.14 | -2.42 | -3.65 | -3.03 | 0.87 |
| *SAUSA300_2620* | 1193.46 | 1122.56 | 433.45 | 338.52 | -2.75 | -3.32 | -3.03 | 0.40 |
| *SAUSA300_0329* | 533.63 | 593.36 | 171.36 | 200.21 | -3.11 | -2.96 | -3.04 | 0.11 |
| *SAUSA300_2491* | 3903.69 | 3209.44 | 1239.87 | 1091.97 | -3.15 | -2.94 | -3.04 | 0.15 |
| *SAUSA300_0303* | 72.11 | 83.51 | 32.99 | 21.40 | -2.19 | -3.90 | -3.04 | 1.21 |
| *murQ* | 536.04 | 464.14 | 137.46 | 207.85 | -3.90 | -2.23 | -3.07 | 1.18 |
| *SAUSA300_0301* | 27.64 | 32.53 | 7.33 | 13.75 | -3.77 | -2.36 | -3.07 | 0.99 |
| *SAUSA300_0718* | 61.30 | 36.04 | 14.66 | 18.34 | -4.18 | -1.97 | -3.07 | 1.57 |
| *SAUSA300_2447* | 882.18 | 810.49 | 277.67 | 271.27 | -3.18 | -2.99 | -3.08 | 0.13 |
| *purH* | 347.34 | 311.19 | 127.38 | 90.17 | -2.73 | -3.45 | -3.09 | 0.51 |
| *purD* | 507.19 | 487.00 | 187.86 | 137.55 | -2.70 | -3.54 | -3.12 | 0.59 |
| *SAUSA300_1803* | 754.78 | 713.79 | 236.43 | 233.83 | -3.19 | -3.05 | -3.12 | 0.10 |
| *aroB* | 223.55 | 216.25 | 59.57 | 85.58 | -3.75 | -2.53 | -3.14 | 0.87 |
| *SAUSA300_0247* | 949.48 | 950.26 | 315.24 | 289.61 | -3.01 | -3.28 | -3.15 | 0.19 |
| *SAUSA300_2339* | 697.09 | 642.59 | 192.44 | 236.12 | -3.62 | -2.72 | -3.17 | 0.64 |
| *cidB* | 686.27 | 637.32 | 188.78 | 233.83 | -3.64 | -2.73 | -3.18 | 0.64 |
| *SAUSA300_0668* | 699.49 | 646.11 | 239.18 | 187.98 | -2.92 | -3.44 | -3.18 | 0.36 |
| *rsbV* | 473.54 | 537.10 | 146.62 | 170.40 | -3.23 | -3.15 | -3.19 | 0.05 |
| *SAUSA300_2436* | 173.07 | 185.48 | 50.40 | 62.66 | -3.43 | -2.96 | -3.20 | 0.33 |
| *SAUSA300_1911* | 761.99 | 718.19 | 246.51 | 215.49 | -3.09 | -3.33 | -3.21 | 0.17 |
| *SAUSA300_1804* | 2629.70 | 2429.71 | 784.43 | 790.89 | -3.35 | -3.07 | -3.21 | 0.20 |
| *SAUSA300_0316* | 63.70 | 50.11 | 17.41 | 17.58 | -3.66 | -2.85 | -3.25 | 0.57 |
| *SAUSA300_1702* | 465.13 | 418.43 | 161.28 | 115.39 | -2.88 | -3.63 | -3.26 | 0.52 |
| *guaB* | 2258.32 | 2049.08 | 656.13 | 667.10 | -3.44 | -3.07 | -3.26 | 0.26 |
| *hutU* | 359.36 | 334.92 | 103.55 | 106.98 | -3.47 | -3.13 | -3.30 | 0.24 |
| *SAUSA300_2041* | 1843.68 | 1981.39 | 747.77 | 479.12 | -2.47 | -4.14 | -3.30 | 1.18 |
| *SAUSA300_2626* | 93.75 | 123.95 | 32.99 | 32.86 | -2.84 | -3.77 | -3.31 | 0.66 |
| *SAUSA300_0674* | 1503.55 | 1433.74 | 447.20 | 437.86 | -3.36 | -3.27 | -3.32 | 0.06 |
| *SAUSA300_0709* | 207.92 | 230.31 | 66.90 | 64.95 | -3.11 | -3.55 | -3.33 | 0.31 |
| *aroC* | 284.84 | 240.86 | 71.48 | 89.41 | -3.99 | -2.69 | -3.34 | 0.91 |
| *SAUSA300_1669* | 104.56 | 74.72 | 27.49 | 25.98 | -3.80 | -2.88 | -3.34 | 0.66 |
| *SAUSA300_0595* | 151.44 | 160.87 | 40.32 | 55.02 | -3.76 | -2.92 | -3.34 | 0.59 |
| *SAUSA300_2248* | 209.13 | 174.93 | 51.32 | 66.48 | -4.08 | -2.63 | -3.35 | 1.02 |
| *SAUSA300_0749* | 1347.30 | 1298.37 | 390.38 | 393.54 | -3.45 | -3.30 | -3.38 | 0.11 |
| *SAUSA300_2614* | 64.90 | 81.75 | 21.08 | 22.16 | -3.08 | -3.69 | -3.38 | 0.43 |
| *SAUSA300_2518* | 562.48 | 494.91 | 175.95 | 138.31 | -3.20 | -3.58 | -3.39 | 0.27 |
| *SAUSA300_2311* | 198.31 | 365.69 | 81.56 | 84.06 | -2.43 | -4.35 | -3.39 | 1.36 |
| *SAUSA300_1184* | 40.86 | 61.53 | 11.91 | 18.34 | -3.43 | -3.36 | -3.39 | 0.05 |
| *potD* | 469.93 | 505.46 | 148.45 | 137.55 | -3.17 | -3.67 | -3.42 | 0.36 |
| *SAUSA300_0409* | 72.11 | 67.69 | 32.99 | 14.52 | -2.19 | -4.66 | -3.42 | 1.75 |
| *SAUSA300_2338* | 1641.76 | 1657.02 | 472.86 | 489.05 | -3.47 | -3.39 | -3.43 | 0.06 |
| *sdhB* | 6409.60 | 5443.12 | 1710.89 | 1742.26 | -3.75 | -3.12 | -3.44 | 0.44 |
| *SAUSA300_2310* | 230.76 | 526.56 | 113.63 | 108.51 | -2.03 | -4.85 | -3.44 | 2.00 |
| *SAUSA300_0351* | 158.65 | 177.57 | 37.57 | 66.48 | -4.22 | -2.67 | -3.45 | 1.10 |
| *phnC* | 69.71 | 55.38 | 18.33 | 17.58 | -3.80 | -3.15 | -3.48 | 0.46 |
| *scrR* | 135.81 | 128.34 | 32.07 | 46.61 | -4.23 | -2.75 | -3.49 | 1.05 |
| *SAUSA300_1903* | 168.26 | 137.13 | 37.57 | 54.25 | -4.48 | -2.53 | -3.50 | 1.38 |
| *SAUSA300_0817* | 687.47 | 668.08 | 197.94 | 188.74 | -3.47 | -3.54 | -3.51 | 0.05 |
| *SAUSA300_2466* | 15.62 | 38.68 | 5.50 | 9.17 | -2.84 | -4.22 | -3.53 | 0.97 |
| *hutI* | 698.29 | 519.52 | 176.86 | 165.06 | -3.95 | -3.15 | -3.55 | 0.57 |
| *SAUSA300_2236* | 3139.30 | 3472.27 | 972.29 | 895.58 | -3.23 | -3.88 | -3.55 | 0.46 |
| *cidC* | 3279.92 | 3123.29 | 981.45 | 827.57 | -3.34 | -3.77 | -3.56 | 0.31 |
| *SAUSA300_0673* | 1068.47 | 1008.28 | 275.83 | 309.48 | -3.87 | -3.26 | -3.57 | 0.44 |
| *SAUSA300_2056* | 241.58 | 233.83 | 70.56 | 61.13 | -3.42 | -3.82 | -3.62 | 0.28 |
| *nrdG* | 13953.77 | 14296.10 | 3976.20 | 3797.05 | -3.51 | -3.77 | -3.64 | 0.18 |
| *SAUSA300_1897* | 842.51 | 864.11 | 219.02 | 249.88 | -3.85 | -3.46 | -3.65 | 0.27 |
| *SAUSA300_0392* | 34.85 | 15.82 | 7.33 | 6.11 | -4.75 | -2.59 | -3.67 | 1.53 |
| *SAUSA300_0300* | 20.43 | 32.53 | 6.41 | 7.64 | -3.19 | -4.26 | -3.72 | 0.76 |
| *sufD* | 3144.10 | 2927.26 | 751.44 | 875.71 | -4.18 | -3.34 | -3.76 | 0.59 |
| *phoP* | 151.44 | 127.46 | 29.32 | 52.73 | -5.16 | -2.42 | -3.79 | 1.94 |
| *SAUSA300_0200* | 545.65 | 563.48 | 140.21 | 152.07 | -3.89 | -3.71 | -3.80 | 0.13 |
| *SAUSA300_1770* | 25.24 | 25.49 | 7.33 | 6.11 | -3.44 | -4.17 | -3.81 | 0.51 |
| *SAUSA300_0579* | 75.72 | 73.84 | 21.99 | 17.58 | -3.44 | -4.20 | -3.82 | 0.54 |
| *SAUSA300_0821* | 1150.19 | 1096.18 | 290.49 | 292.67 | -3.96 | -3.75 | -3.85 | 0.15 |
| *SAUSA300_1590* | 4068.35 | 3777.31 | 1039.18 | 976.58 | -3.91 | -3.87 | -3.89 | 0.03 |
| *sufS* | 2861.66 | 2551.02 | 655.22 | 745.81 | -4.37 | -3.42 | -3.89 | 0.67 |
| *SAUSA300_2257* | 5388.01 | 5034.36 | 1491.88 | 1192.83 | -3.61 | -4.22 | -3.92 | 0.43 |
| *SAUSA300_2258* | 28412.32 | 26734.76 | 6894.89 | 7142.49 | -4.12 | -3.74 | -3.93 | 0.27 |
| *SAUSA300_0675* | 282.44 | 289.21 | 96.22 | 58.08 | -2.94 | -4.98 | -3.96 | 1.45 |
| *purM* | 194.70 | 164.38 | 46.74 | 43.56 | -4.17 | -3.77 | -3.97 | 0.28 |
| *SAUSA300_0223* | 39.66 | 33.40 | 12.83 | 6.88 | -3.09 | -4.86 | -3.97 | 1.25 |
| *sdhA* | 7988.86 | 7284.74 | 1832.77 | 2001.30 | -4.36 | -3.64 | -4.00 | 0.51 |
| *SAUSA300_1438* | 73.31 | 58.02 | 24.74 | 11.46 | -2.96 | -5.06 | -4.01 | 1.48 |
| *SAUSA300_0027* | 578.10 | 487.88 | 162.20 | 109.27 | -3.56 | -4.46 | -4.01 | 0.64 |
| *SAUSA300_0676* | 2552.78 | 2158.08 | 569.99 | 607.50 | -4.48 | -3.55 | -4.02 | 0.65 |
| *SAUSA300_2398* | 447.10 | 445.68 | 117.30 | 104.69 | -3.81 | -4.26 | -4.03 | 0.32 |
| *SAUSA300_0309* | 538.44 | 443.92 | 112.72 | 133.73 | -4.78 | -3.32 | -4.05 | 1.03 |
| *SAUSA300_2632* | 1225.91 | 1218.37 | 360.14 | 258.28 | -3.40 | -4.72 | -4.06 | 0.93 |
| *SAUSA300_1229* | 3858.02 | 3618.20 | 929.22 | 909.34 | -4.15 | -3.98 | -4.07 | 0.12 |
| *SAUSA300_2279* | 72.11 | 49.23 | 17.41 | 12.23 | -4.14 | -4.03 | -4.08 | 0.08 |
| *SAUSA300_1671* | 567.28 | 578.42 | 144.79 | 135.25 | -3.92 | -4.28 | -4.10 | 0.25 |
| *SAUSA300_2454* | 699.49 | 718.19 | 193.36 | 152.83 | -3.62 | -4.70 | -4.16 | 0.76 |
| *SAUSA300_2132* | 1361.72 | 1412.64 | 319.82 | 343.87 | -4.26 | -4.11 | -4.18 | 0.11 |
| *fmtB* | 520.41 | 448.32 | 110.88 | 120.74 | -4.69 | -3.71 | -4.20 | 0.69 |
| *SAUSA300_0147* | 439.89 | 465.02 | 128.29 | 93.23 | -3.43 | -4.99 | -4.21 | 1.10 |
| *ggt* | 527.62 | 540.62 | 106.30 | 153.59 | -4.96 | -3.52 | -4.24 | 1.02 |
| *sdrC* | 195.91 | 140.65 | 35.74 | 45.85 | -5.48 | -3.07 | -4.27 | 1.71 |
| *SAUSA300_0804* | 19.23 | 19.34 | 5.50 | 3.82 | -3.50 | -5.06 | -4.28 | 1.11 |
| *sdhC* | 1306.44 | 1262.33 | 358.31 | 256.75 | -3.65 | -4.92 | -4.28 | 0.90 |
| *hutG* | 1073.27 | 1010.92 | 252.01 | 234.59 | -4.26 | -4.31 | -4.28 | 0.04 |
| *nrdD* | 27284.96 | 26787.50 | 6071.06 | 6534.99 | -4.49 | -4.10 | -4.30 | 0.28 |
| *SAUSA300_0169* | 74.52 | 62.41 | 21.08 | 12.23 | -3.54 | -5.10 | -4.32 | 1.11 |
| *SAUSA300_1929* | 9.61 | 18.46 | 3.67 | 3.06 | -2.62 | -6.04 | -4.33 | 2.42 |
| *SAUSA300_1986* | 530.03 | 421.07 | 118.21 | 98.58 | -4.48 | -4.27 | -4.38 | 0.15 |
| *serA* | 129.80 | 152.08 | 24.74 | 42.79 | -5.25 | -3.55 | -4.40 | 1.20 |
| *SAUSA300_0145* | 126.20 | 88.78 | 28.41 | 19.87 | -4.44 | -4.47 | -4.46 | 0.02 |
| *SAUSA300_0438* | 1926.61 | 1875.91 | 406.88 | 448.55 | -4.74 | -4.18 | -4.46 | 0.39 |
| *SAUSA300_0106* | 186.29 | 218.89 | 52.23 | 40.50 | -3.57 | -5.40 | -4.49 | 1.30 |
| *lip* | 1544.41 | 1366.05 | 312.49 | 338.52 | -4.94 | -4.04 | -4.49 | 0.64 |
| *SAUSA300_2097* | 390.61 | 406.12 | 93.47 | 84.06 | -4.18 | -4.83 | -4.51 | 0.46 |
| *SAUSA300_1259* | 55.29 | 42.19 | 7.33 | 28.27 | -7.54 | -1.49 | -4.52 | 4.28 |
| *SAUSA300_2324* | 133.41 | 138.01 | 25.66 | 35.91 | -5.20 | -3.84 | -4.52 | 0.96 |
| *SAUSA300_1056* | 18.03 | 15.82 | 3.67 | 3.82 | -4.92 | -4.14 | -4.53 | 0.55 |
| *SAUSA300_0122* | 16.83 | 27.25 | 2.75 | 9.17 | -6.12 | -2.97 | -4.55 | 2.23 |
| *SAUSA300_2496* | 544.45 | 502.82 | 115.46 | 113.86 | -4.72 | -4.42 | -4.57 | 0.21 |
| *SAUSA300_0201* | 64.90 | 94.06 | 12.83 | 22.92 | -5.06 | -4.10 | -4.58 | 0.68 |
| *SAUSA300_2575* | 895.40 | 857.96 | 169.53 | 220.07 | -5.28 | -3.90 | -4.59 | 0.98 |
| *SAUSA300_0206* | 596.13 | 573.15 | 142.04 | 114.62 | -4.20 | -5.00 | -4.60 | 0.57 |
| *fnbA* | 156.24 | 153.83 | 32.07 | 35.15 | -4.87 | -4.38 | -4.62 | 0.35 |
| *SAUSA300_0664* | 246.38 | 260.20 | 70.56 | 45.08 | -3.49 | -5.77 | -4.63 | 1.61 |
| *hisD* | 28.84 | 18.46 | 4.58 | 6.11 | -6.30 | -3.02 | -4.66 | 2.32 |
| *SAUSA300_0586* | 24.04 | 10.55 | 3.67 | 3.82 | -6.56 | -2.76 | -4.66 | 2.68 |
| *SAUSA300_0026* | 263.21 | 276.02 | 73.31 | 48.14 | -3.59 | -5.73 | -4.66 | 1.52 |
| *gltC* | 18.03 | 43.95 | 8.25 | 6.11 | -2.19 | -7.19 | -4.69 | 3.54 |
| *purN* | 115.38 | 69.45 | 24.74 | 14.52 | -4.66 | -4.78 | -4.72 | 0.08 |
| *yfiA* | 10491.17 | 9291.63 | 2604.37 | 1699.46 | -4.03 | -5.47 | -4.75 | 1.02 |
| *SAUSA300_0308* | 466.33 | 420.19 | 87.06 | 100.87 | -5.36 | -4.17 | -4.76 | 0.84 |
| *SAUSA300_0222* | 1704.26 | 1871.51 | 344.56 | 408.05 | -4.95 | -4.59 | -4.77 | 0.25 |
| *SAUSA300_0538* | 1471.09 | 1205.19 | 267.58 | 291.14 | -5.50 | -4.14 | -4.82 | 0.96 |
| *SAUSA300_0199* | 317.29 | 246.14 | 50.40 | 71.07 | -6.30 | -3.46 | -4.88 | 2.00 |
| *SAUSA300_0677* | 4830.34 | 4387.37 | 920.97 | 965.88 | -5.24 | -4.54 | -4.89 | 0.50 |
| *SAUSA300_2416* | 459.12 | 397.33 | 98.97 | 76.41 | -4.64 | -5.20 | -4.92 | 0.40 |
| *SAUSA300_0172* | 20.43 | 28.13 | 8.25 | 3.82 | -2.48 | -7.36 | -4.92 | 3.45 |
| *sirC* | 689.88 | 692.70 | 141.12 | 139.07 | -4.89 | -4.98 | -4.93 | 0.07 |
| *narK* | 2493.89 | 2389.28 | 528.75 | 460.78 | -4.72 | -5.19 | -4.95 | 0.33 |
| *SAUSA300_0809* | 124.99 | 108.12 | 24.74 | 22.16 | -5.05 | -4.88 | -4.97 | 0.12 |
| *SAUSA300_0299* | 34.85 | 36.04 | 5.50 | 9.93 | -6.34 | -3.63 | -4.98 | 1.92 |
| *SAUSA300_0720* | 18.03 | 19.34 | 3.67 | 3.82 | -4.92 | -5.06 | -4.99 | 0.10 |
| *SAUSA300_0312* | 32.45 | 32.53 | 8.25 | 5.35 | -3.93 | -6.08 | -5.01 | 1.52 |
| *SAUSA300_2315* | 2241.50 | 2196.76 | 498.51 | 396.59 | -4.50 | -5.54 | -5.02 | 0.74 |
| *SAUSA300_1510* | 164.66 | 128.34 | 32.99 | 25.22 | -4.99 | -5.09 | -5.04 | 0.07 |
| *hisC* | 143.02 | 141.53 | 34.82 | 23.69 | -4.11 | -5.97 | -5.04 | 1.32 |
| *SAUSA300_2399* | 164.66 | 177.57 | 33.91 | 33.62 | -4.86 | -5.28 | -5.07 | 0.30 |
| *narI* | 643.00 | 601.27 | 120.96 | 124.56 | -5.32 | -4.83 | -5.07 | 0.35 |
| *hlgA* | 145.43 | 87.91 | 29.32 | 16.81 | -4.96 | -5.23 | -5.09 | 0.19 |
| *SAUSA300_0314* | 283.64 | 263.72 | 49.48 | 58.08 | -5.73 | -4.54 | -5.14 | 0.84 |
| *SAUSA300_2616* | 44.47 | 70.32 | 7.33 | 16.05 | -6.07 | -4.38 | -5.22 | 1.19 |
| *SAUSA300_0235* | 64966.15 | 66679.10 | 13173.97 | 12024.63 | -4.93 | -5.55 | -5.24 | 0.43 |
| *ddh* | 14897.24 | 12577.55 | 2910.44 | 2336.76 | -5.12 | -5.38 | -5.25 | 0.19 |
| *SAUSA300_2415* | 108.17 | 128.34 | 17.41 | 29.04 | -6.21 | -4.42 | -5.32 | 1.27 |
| *purF* | 206.72 | 203.94 | 27.49 | 62.66 | -7.52 | -3.25 | -5.39 | 3.02 |
| *SAUSA300_0108* | 824.49 | 690.94 | 160.37 | 122.26 | -5.14 | -5.65 | -5.40 | 0.36 |
| *SAUSA300_2164* | 419.45 | 413.16 | 65.98 | 90.93 | -6.36 | -4.54 | -5.45 | 1.28 |
| *lysP* | 3644.08 | 4432.20 | 763.35 | 721.36 | -4.77 | -6.14 | -5.46 | 0.97 |
| *SAUSA300_0114* | 1078.08 | 953.78 | 217.18 | 156.65 | -4.96 | -6.09 | -5.53 | 0.80 |
| *SAUSA300_0712* | 4400.06 | 4809.32 | 833.00 | 829.10 | -5.28 | -5.80 | -5.54 | 0.37 |
| *SAUSA300_0070* | 636.99 | 693.58 | 132.88 | 110.04 | -4.79 | -6.30 | -5.55 | 1.07 |
| *acsA* | 563.68 | 417.55 | 96.22 | 79.47 | -5.86 | -5.25 | -5.56 | 0.43 |
| *SAUSA300_0105* | 183.89 | 160.87 | 36.66 | 25.98 | -5.02 | -6.19 | -5.60 | 0.83 |
| *purL* | 229.56 | 236.47 | 38.49 | 45.08 | -5.96 | -5.24 | -5.60 | 0.51 |
| *SAUSA300_0198* | 306.48 | 245.26 | 50.40 | 47.38 | -6.08 | -5.18 | -5.63 | 0.64 |
| *epiE* | 200.71 | 195.15 | 47.65 | 27.51 | -4.21 | -7.09 | -5.65 | 2.04 |
| *hisA* | 19.23 | 21.98 | 4.58 | 3.06 | -4.20 | -7.19 | -5.69 | 2.12 |
| *SAUSA300_1232* | 1437.44 | 1546.26 | 266.67 | 252.17 | -5.39 | -6.13 | -5.76 | 0.52 |
| *SAUSA300_1328* | 15335.92 | 18450.52 | 2979.17 | 2731.06 | -5.15 | -6.76 | -5.95 | 1.14 |
| *SAUSA300_1856* | 1257.16 | 1059.26 | 172.28 | 219.31 | -7.30 | -4.83 | -6.06 | 1.74 |
| *SAUSA300_2574* | 62.50 | 45.71 | 8.25 | 9.93 | -7.58 | -4.60 | -6.09 | 2.10 |
| *crtN* | 837.71 | 828.07 | 147.54 | 124.56 | -5.68 | -6.65 | -6.16 | 0.69 |
| *SAUSA300_1934* | 19.23 | 14.06 | 1.83 | 7.64 | -10.49 | -1.84 | -6.17 | 6.12 |
| *SAUSA300_0067* | 3635.67 | 3266.58 | 608.48 | 508.92 | -5.98 | -6.42 | -6.20 | 0.31 |
| *SAUSA300_0786* | 405.03 | 323.49 | 74.23 | 45.85 | -5.46 | -7.06 | -6.26 | 1.13 |
| *SAUSA300_2276* | 143.02 | 145.04 | 17.41 | 33.62 | -8.21 | -4.31 | -6.26 | 2.76 |
| *mtlA* | 361.76 | 305.91 | 54.07 | 51.20 | -6.69 | -5.98 | -6.33 | 0.51 |
| *SAUSA300_2502* | 102.16 | 123.95 | 38.49 | 12.23 | -2.65 | -10.14 | -6.40 | 5.29 |
| *SAUSA300_1582* | 1404.99 | 1319.46 | 265.75 | 175.75 | -5.29 | -7.51 | -6.40 | 1.57 |
| *SAUSA300_0982* | 1652.58 | 1515.49 | 259.34 | 233.07 | -6.37 | -6.50 | -6.44 | 0.09 |
| *SAUSA300_1329* | 27727.25 | 33113.19 | 4869.68 | 4608.58 | -5.69 | -7.19 | -6.44 | 1.05 |
| *clfA* | 6997.32 | 6435.58 | 1156.48 | 939.14 | -6.05 | -6.85 | -6.45 | 0.57 |
| *SAUSA300_2543* | 217.54 | 181.96 | 32.99 | 28.27 | -6.59 | -6.44 | -6.52 | 0.11 |
| *SAUSA300_2344* | 2734.27 | 2690.79 | 400.46 | 421.05 | -6.83 | -6.39 | -6.61 | 0.31 |
| *SAUSA300_0129* | 8548.94 | 7242.55 | 1331.51 | 1056.05 | -6.42 | -6.86 | -6.64 | 0.31 |
| *SAUSA300_0208* | 57.69 | 50.11 | 7.33 | 9.17 | -7.87 | -5.46 | -6.67 | 1.70 |
| *purK* | 72.11 | 58.90 | 14.66 | 6.88 | -4.92 | -8.56 | -6.74 | 2.58 |
| *atl* | 4202.96 | 4271.34 | 602.98 | 651.05 | -6.97 | -6.56 | -6.77 | 0.29 |
| *SAUSA300_1771* | 439.89 | 393.82 | 62.31 | 60.37 | -7.06 | -6.52 | -6.79 | 0.38 |
| *uhpT* | 4706.54 | 3331.63 | 597.48 | 574.64 | -7.88 | -5.80 | -6.84 | 1.47 |
| *SAUSA300_0111* | 162.25 | 123.95 | 21.08 | 19.87 | -7.70 | -6.24 | -6.97 | 1.03 |
| *SAUSA300_1581* | 1026.40 | 899.28 | 167.70 | 114.62 | -6.12 | -7.85 | -6.98 | 1.22 |
| *SAUSA300_2453* | 610.55 | 495.79 | 70.56 | 92.46 | -8.65 | -5.36 | -7.01 | 2.33 |
| *SAUSA300_2418* | 1088.90 | 946.74 | 155.79 | 133.73 | -6.99 | -7.08 | -7.03 | 0.06 |
| *SAUSA300_0805* | 21.63 | 23.73 | 2.75 | 3.82 | -7.87 | -6.21 | -7.04 | 1.17 |
| *SAUSA300_0719* | 33.65 | 38.68 | 8.25 | 3.82 | -4.08 | -10.12 | -7.10 | 4.27 |
| *acuC* | 432.67 | 400.85 | 56.82 | 60.37 | -7.62 | -6.64 | -7.13 | 0.69 |
| *epiG* | 293.26 | 249.65 | 32.07 | 48.14 | -9.14 | -5.19 | -7.16 | 2.80 |
| *SAUSA300_0374* | 6163.21 | 6161.31 | 1132.65 | 690.03 | -5.44 | -8.93 | -7.19 | 2.47 |
| *asp23* | 27919.55 | 23357.42 | 4087.08 | 3038.25 | -6.83 | -7.69 | -7.26 | 0.61 |
| *mtlD* | 905.01 | 923.89 | 118.21 | 134.49 | -7.66 | -6.87 | -7.26 | 0.56 |
| *SAUSA300_0012* | 313.69 | 350.74 | 40.32 | 51.96 | -7.78 | -6.75 | -7.26 | 0.73 |
| *SAUSA300_0952* | 372.58 | 359.53 | 48.57 | 51.96 | -7.67 | -6.92 | -7.30 | 0.53 |
| *SAUSA300_0816* | 16252.95 | 15045.94 | 2717.09 | 1674.25 | -5.98 | -8.99 | -7.48 | 2.12 |
| *SAUSA300_2086* | 219.94 | 265.48 | 30.24 | 34.39 | -7.27 | -7.72 | -7.50 | 0.32 |
| *SAUSA300_2343* | 3877.25 | 3844.99 | 559.91 | 469.19 | -6.92 | -8.20 | -7.56 | 0.90 |
| *SAUSA300_2629* | 38.46 | 47.47 | 4.58 | 6.88 | -8.39 | -6.90 | -7.65 | 1.05 |
| *SAUSA300_0540* | 801.65 | 814.89 | 96.22 | 116.91 | -8.33 | -6.97 | -7.65 | 0.96 |
| *SAUSA300_2538* | 615.36 | 468.54 | 78.81 | 61.13 | -7.81 | -7.66 | -7.74 | 0.10 |
| *epiP* | 207.92 | 213.61 | 43.07 | 19.87 | -4.83 | -10.75 | -7.79 | 4.19 |
| *SAUSA300_0784* | 274.03 | 252.29 | 33.91 | 33.62 | -8.08 | -7.50 | -7.79 | 0.41 |
| *SAUSA300_1966* | 36.06 | 41.32 | 4.58 | 5.35 | -7.87 | -7.72 | -7.80 | 0.10 |
| *SAUSA300_2106* | 1569.65 | 1373.09 | 192.44 | 183.40 | -8.16 | -7.49 | -7.82 | 0.47 |
| *SAUSA300_0782* | 1318.46 | 1120.80 | 178.70 | 131.43 | -7.38 | -8.53 | -7.95 | 0.81 |
| *SAUSA300_0604* | 718.72 | 751.59 | 110.88 | 78.71 | -6.48 | -9.55 | -8.02 | 2.17 |
| *SAUSA300_1935* | 8.41 | 5.27 | 0.92 | 0.76 | -9.18 | -6.90 | -8.04 | 1.61 |
| *SAUSA300_1976* | 438.68 | 362.17 | 47.65 | 50.43 | -9.21 | -7.18 | -8.19 | 1.43 |
| *SAUSA300_0311* | 26.44 | 16.70 | 1.83 | 8.41 | -14.43 | -1.99 | -8.21 | 8.80 |
| *SAUSA300_2275* | 1516.77 | 1293.09 | 189.69 | 153.59 | -8.00 | -8.42 | -8.21 | 0.30 |
| *SAUSA300_0372* | 2343.66 | 2119.41 | 287.75 | 252.17 | -8.14 | -8.40 | -8.27 | 0.18 |
| *SAUSA300_2087* | 118.99 | 83.51 | 12.83 | 11.46 | -9.27 | -7.29 | -8.28 | 1.41 |
| *SAUSA300_1212* | 61.30 | 43.95 | 6.41 | 6.11 | -9.56 | -7.19 | -8.37 | 1.67 |
| *narH* | 1433.84 | 1618.34 | 190.61 | 174.99 | -7.52 | -9.25 | -8.39 | 1.22 |
| *crtM* | 286.05 | 301.52 | 33.91 | 35.91 | -8.44 | -8.40 | -8.42 | 0.03 |
| *SAUSA300_2539* | 192.30 | 143.29 | 21.99 | 17.58 | -8.74 | -8.15 | -8.45 | 0.42 |
| *alr2* | 75.72 | 68.57 | 9.16 | 7.64 | -8.26 | -8.97 | -8.62 | 0.50 |
| *SAUSA300_0305* | 1281.20 | 1380.12 | 146.62 | 155.12 | -8.74 | -8.90 | -8.82 | 0.11 |
| *SAUSA300_1960* | 26.44 | 30.77 | 2.75 | 3.82 | -9.62 | -8.05 | -8.84 | 1.11 |
| *SAUSA300_0954* | 42.07 | 33.40 | 3.67 | 5.35 | -11.48 | -6.24 | -8.86 | 3.70 |
| *SAUSA300_0425* | 235.57 | 223.28 | 29.32 | 22.92 | -8.03 | -9.74 | -8.89 | 1.21 |
| *narJ* | 378.59 | 507.22 | 55.90 | 45.85 | -6.77 | -11.06 | -8.92 | 3.03 |
| *SAUSA300_0113* | 14141.26 | 13105.86 | 1715.48 | 1360.18 | -8.24 | -9.64 | -8.94 | 0.98 |
| *SAUSA300_0781* | 800.45 | 727.86 | 90.72 | 80.24 | -8.82 | -9.07 | -8.95 | 0.18 |
| *gntR* | 512.00 | 575.78 | 65.06 | 56.55 | -7.87 | -10.18 | -9.03 | 1.64 |
| *nirB* | 2686.19 | 2719.80 | 341.81 | 255.23 | -7.86 | -10.66 | -9.26 | 1.98 |
| *SAUSA300_2327* | 2103.28 | 1810.86 | 205.27 | 210.90 | -10.25 | -8.59 | -9.42 | 1.17 |
| *SAUSA300_2403* | 697.09 | 558.20 | 86.14 | 51.20 | -8.09 | -10.90 | -9.50 | 1.99 |
| *comK* | 28.84 | 29.89 | 1.83 | 9.17 | -15.74 | -3.26 | -9.50 | 8.82 |
| *nanA* | 187.49 | 147.68 | 14.66 | 23.69 | -12.79 | -6.23 | -9.51 | 4.63 |
| *SAUSA300_1456* | 1034.81 | 759.51 | 90.72 | 98.58 | -11.41 | -7.70 | -9.56 | 2.62 |
| *SAUSA300_2144* | 17212.05 | 15616.45 | 1873.09 | 1500.02 | -9.19 | -10.41 | -9.80 | 0.86 |
| *SAUSA300_1744* | 32.45 | 33.40 | 6.41 | 2.29 | -5.06 | -14.57 | -9.82 | 6.73 |
| *acuA* | 211.53 | 181.96 | 16.49 | 25.22 | -12.82 | -7.22 | -10.02 | 3.97 |
| *SAUSA300_2486* | 17663.96 | 14571.25 | 1569.77 | 1642.92 | -11.25 | -8.87 | -10.06 | 1.69 |
| *SAUSA300_2615* | 10.82 | 26.37 | 3.67 | 1.53 | -2.95 | -17.26 | -10.10 | 10.11 |
| *SAUSA300_0010* | 58.89 | 52.74 | 6.41 | 4.58 | -9.18 | -11.50 | -10.34 | 1.64 |
| *SAUSA300_1957* | 8.41 | 12.31 | 1.83 | 0.76 | -4.59 | -16.11 | -10.35 | 8.14 |
| *nirD* | 432.67 | 478.21 | 39.40 | 48.14 | -10.98 | -9.93 | -10.46 | 0.74 |
| *adhE* | 7119.91 | 6614.90 | 691.87 | 603.68 | -10.29 | -10.96 | -10.62 | 0.47 |
| *SAUSA300_1030* | 38.46 | 23.73 | 6.41 | 1.53 | -6.00 | -15.53 | -10.76 | 6.74 |
| *SAUSA300_2525* | 1263.17 | 1131.35 | 121.88 | 100.87 | -10.36 | -11.22 | -10.79 | 0.60 |
| *adh* | 46966.87 | 43893.07 | 4237.37 | 4116.46 | -11.08 | -10.66 | -10.87 | 0.30 |
| *SAUSA300_2313* | 30554.06 | 27265.71 | 2704.26 | 2585.88 | -11.30 | -10.54 | -10.92 | 0.53 |
| *SAUSA300_2500* | 455.51 | 410.52 | 40.32 | 38.21 | -11.30 | -10.74 | -11.02 | 0.39 |
| *SAUSA300_2305* | 6.01 | 23.73 | 0.92 | 1.53 | -6.56 | -15.53 | -11.04 | 6.34 |
| *SAUSA300_2544* | 31.25 | 63.29 | 20.16 | 3.06 | -1.55 | -20.71 | -11.13 | 13.55 |
| *SAUSA300_1211* | 51.68 | 45.71 | 3.67 | 5.35 | -14.10 | -8.55 | -11.32 | 3.93 |
| *SAUSA300_2143* | 4467.37 | 3897.74 | 438.95 | 307.19 | -10.18 | -12.69 | -11.43 | 1.78 |
| *SAUSA300_1656* | 41849.29 | 31887.79 | 3527.17 | 2871.67 | -11.86 | -11.10 | -11.48 | 0.54 |
| *SAUSA300_0274* | 54.08 | 31.65 | 3.67 | 3.82 | -14.75 | -8.28 | -11.52 | 4.58 |
| *metE* | 597.33 | 519.52 | 50.40 | 45.08 | -11.85 | -11.52 | -11.69 | 0.23 |
| *SAUSA300_1479* | 22.84 | 14.06 | 4.58 | 0.76 | -4.98 | -18.41 | -11.69 | 9.49 |
| *putA* | 3596.01 | 3377.34 | 327.15 | 272.04 | -10.99 | -12.42 | -11.70 | 1.01 |
| *ilvA* | 30438.68 | 32002.94 | 2598.87 | 2540.79 | -11.71 | -12.60 | -12.15 | 0.62 |
| *SAUSA300_2501* | 543.25 | 461.50 | 48.57 | 33.62 | -11.19 | -13.73 | -12.46 | 1.80 |
| *SAUSA300_0229* | 126.20 | 88.78 | 8.25 | 9.17 | -15.30 | -9.68 | -12.49 | 3.97 |
| *epiF* | 395.42 | 374.48 | 25.66 | 38.21 | -15.41 | -9.80 | -12.61 | 3.97 |
| *leuB* | 182.68 | 117.79 | 12.83 | 10.70 | -14.24 | -11.01 | -12.63 | 2.28 |
| *mtlF* | 3731.82 | 3584.79 | 270.33 | 288.85 | -13.80 | -12.41 | -13.11 | 0.99 |
| *SAUSA300_0011* | 110.57 | 75.60 | 6.41 | 8.41 | -17.24 | -8.99 | -13.12 | 5.83 |
| *gntK* | 1153.80 | 1077.72 | 90.72 | 79.47 | -12.72 | -13.56 | -13.14 | 0.60 |
| *SAUSA300_0202* | 96.15 | 132.74 | 8.25 | 8.41 | -11.66 | -15.79 | -13.72 | 2.92 |
| *SAUSA300_2145* | 579.30 | 591.61 | 50.40 | 36.68 | -11.49 | -16.13 | -13.81 | 3.28 |
| *SAUSA300_0711* | 1098.51 | 992.46 | 79.73 | 66.48 | -13.78 | -14.93 | -14.35 | 0.81 |
| *SAUSA300_2417* | 469.93 | 411.40 | 30.24 | 30.57 | -15.54 | -13.46 | -14.50 | 1.47 |
| *SAUSA300_0203* | 304.07 | 237.35 | 19.24 | 17.58 | -15.80 | -13.50 | -14.65 | 1.62 |
| *SAUSA300_2617* | 206.72 | 174.05 | 11.91 | 13.75 | -17.35 | -12.65 | -15.00 | 3.32 |
| *gltD* | 3654.90 | 3447.66 | 239.18 | 233.83 | -15.28 | -14.74 | -15.01 | 0.38 |
| *gntP* | 2427.79 | 2029.74 | 137.46 | 163.53 | -17.66 | -12.41 | -15.04 | 3.71 |
| *SAUSA300_1739* | 1271.58 | 1042.56 | 78.81 | 73.36 | -16.13 | -14.21 | -15.17 | 1.36 |
| *SAUSA300_1327* | 7976.84 | 6637.76 | 453.61 | 509.69 | -17.59 | -13.02 | -15.30 | 3.23 |
| *SAUSA300_1740* | 1835.26 | 1296.61 | 126.46 | 74.89 | -14.51 | -17.31 | -15.91 | 1.98 |
| *SAUSA300_1291* | 85.33 | 72.08 | 3.67 | 7.64 | -23.28 | -9.43 | -16.36 | 9.79 |
| *SAUSA300_2459* | 373.78 | 326.13 | 17.41 | 28.27 | -21.47 | -11.53 | -16.50 | 7.02 |
| *ilvD* | 90.14 | 130.98 | 6.41 | 6.88 | -14.05 | -19.05 | -16.55 | 3.53 |
| *SAUSA300_1961* | 32.45 | 20.22 | 4.58 | 0.76 | -7.08 | -26.46 | -16.77 | 13.70 |
| *SAUSA300_0358* | 342.53 | 281.30 | 18.33 | 18.34 | -18.69 | -15.34 | -17.01 | 2.37 |
| *malR* | 192.30 | 175.81 | 12.83 | 9.17 | -14.99 | -19.17 | -17.08 | 2.96 |
| *SAUSA300_2618* | 61.30 | 46.59 | 2.75 | 3.82 | -22.30 | -12.19 | -17.25 | 7.14 |
| *SAUSA300_1210* | 19.23 | 10.55 | 0.92 | 0.76 | -20.98 | -13.80 | -17.39 | 5.08 |
| *SAUSA300_0343* | 217.54 | 190.76 | 11.91 | 11.46 | -18.26 | -16.64 | -17.45 | 1.14 |
| *gltB* | 5656.02 | 5934.51 | 337.23 | 298.78 | -16.77 | -19.86 | -18.32 | 2.19 |
| *leuD* | 117.78 | 59.78 | 3.67 | 11.46 | -32.13 | -5.22 | -18.67 | 19.03 |
| *SAUSA300_2449* | 2466.25 | 2052.60 | 114.55 | 114.62 | -21.53 | -17.91 | -19.72 | 2.56 |
| *ilvB* | 213.93 | 185.48 | 13.75 | 7.64 | -15.56 | -24.27 | -19.92 | 6.16 |
| *thrS* | 123955.37 | 115279.52 | 6177.36 | 5028.09 | -20.07 | -22.93 | -21.50 | 2.02 |
| *SAUSA300_0929* | 13891.27 | 12250.54 | 860.49 | 448.55 | -16.14 | -27.31 | -21.73 | 7.90 |
| *leuC* | 253.60 | 208.34 | 7.33 | 18.34 | -34.59 | -11.36 | -22.98 | 16.43 |
| *sdrD* | 514.40 | 494.91 | 26.58 | 14.52 | -19.36 | -34.09 | -26.72 | 10.42 |
| *SAUSA300_0359* | 176.68 | 118.67 | 4.58 | 5.35 | -38.56 | -22.19 | -30.37 | 11.58 |
| *leuA* | 264.41 | 194.27 | 7.33 | 7.64 | -36.07 | -25.42 | -30.75 | 7.53 |
| *SAUSA300_2619* | 70.91 | 60.65 | 2.75 | 1.53 | -25.79 | -39.69 | -32.74 | 9.82 |
| *SAUSA300_1052* | 116.58 | 75.60 | 1.83 | 8.41 | -63.61 | -8.99 | -36.30 | 38.62 |
| *SAUSA300_0796* | 480.75 | 510.73 | 15.58 | 12.23 | -30.86 | -41.77 | -36.32 | 7.72 |
| *pyc* | 8836.18 | 8902.21 | 216.27 | 184.92 | -40.86 | -48.14 | -44.50 | 5.15 |
| *SAUSA300_1226* | 3995.03 | 3668.30 | 88.89 | 80.24 | -44.94 | -45.72 | -45.33 | 0.55 |
| *oppC* | 774.01 | 796.43 | 16.49 | 15.28 | -46.92 | -52.11 | -49.52 | 3.67 |
| *SAUSA300_0360* | 104.56 | 106.37 | 2.75 | 1.53 | -38.03 | -69.60 | -53.82 | 22.32 |
| *SAUSA300_0435* | 639.40 | 472.93 | 11.00 | 8.41 | -58.14 | -56.26 | -57.20 | 1.33 |
| *thrC* | 4503.43 | 4151.79 | 67.81 | 84.06 | -66.41 | -49.39 | -57.90 | 12.03 |
| *oppB* | 862.95 | 820.16 | 11.00 | 19.87 | -78.47 | -41.28 | -59.88 | 26.30 |
| *SAUSA300_1225* | 634.59 | 539.74 | 11.00 | 8.41 | -57.71 | -64.21 | -60.96 | 4.60 |
| *dapD* | 522.82 | 500.18 | 6.41 | 12.23 | -81.50 | -40.91 | -61.21 | 28.70 |
| *dapB* | 478.35 | 385.03 | 6.41 | 5.35 | -74.57 | -71.98 | -73.28 | 1.83 |
| *thrB* | 4186.13 | 3660.39 | 46.74 | 58.08 | -89.57 | -63.03 | -76.30 | 18.77 |
| *SAUSA300_0437* | 1891.75 | 1627.13 | 26.58 | 13.75 | -71.18 | -118.30 | -94.74 | 33.31 |
| *SAUSA300_2306* | 383.40 | 2870.12 | 19.24 | 16.05 | -19.92 | -178.86 | -99.39 | 112.38 |
| *ilvC* | 155.04 | 125.71 | 0.92 | 2.29 | -169.19 | -54.83 | -112.01 | 80.86 |
| *SAUSA300_0436* | 420.66 | 377.99 | 2.75 | 3.06 | -153.01 | -123.67 | -138.34 | 20.75 |
| *SAUSA300_0798* | 1074.48 | 1025.86 | 5.50 | 10.70 | -195.42 | -95.89 | -145.66 | 70.38 |
| *dapA* | 576.90 | 510.73 | 2.75 | 5.35 | -209.85 | -95.48 | -152.66 | 80.87 |
| *SAUSA300_1286* | 431.47 | 353.38 | 2.75 | 2.29 | -156.95 | -154.15 | -155.55 | 1.98 |
| *SAUSA300_0797* | 439.89 | 385.91 | 7.33 | 1.53 | -60.00 | -252.51 | -156.26 | 136.12 |
| *asd* | 393.01 | 405.25 | 3.67 | 1.53 | -107.22 | -265.16 | -186.19 | 111.68 |
| *oppD* | 1258.36 | 1193.76 | 11.00 | 4.58 | -114.43 | -260.37 | -187.40 | 103.19 |
| *SAUSA300_2307* | 231.96 | 2282.03 | 12.83 | 5.35 | -18.08 | -426.62 | -222.35 | 288.88 |
| *oppF* | 1705.46 | 1724.71 | 3.67 | 6.88 | -465.27 | -250.78 | -358.02 | 151.66 |
| *oppA* | 4234.21 | 3880.16 | 9.16 | 11.46 | -462.05 | -338.52 | -400.29 | 87.35 |
| *trpC*† | 4.81 | 5.27 | 0.00 | 0.00 | 0.00 | 0.00 | -500.00 | 0.00 |
| *SAUSA300_1502*† | 9.61 | 9.67 | 0.00 | 0.00 | 0.00 | 0.00 | -500.00 | 0.00 |

† denotes genes with FC values arbitrarily set "max" fold change as gene is completely induced or repressed by pyruvate
