# Supplementary material for: Staphylococcus aureus Responds to the Central Metabolite Pyruvate To Regulate Virulence
Source: mBio. 2018 Jan 23;9(1):e02272-17. doi: 10.1128/mBio.02272-17 (PMC5784258; doi:10.1128/mBio.02272-17)
Supplement: TABLE S3 [file mbo001183696st3.docx]

**Supplemental Table 3:** **Genes most induced in the presence of of pyruvate**.

| **Accession Number** | ***Gene name*** | **Gene product** | **Avg Fold Change (YCP / YC)^1^** | **Standard Deviation** | **Functional category** |
| --- | --- | --- | --- | --- | --- |
| SAUSA300_1334 | *SAUSA300_1334* | Putative membrane protein | 19.1 | 0.1 | Membrane-associated* |
| SAUSA300_1790 | *prsA* | Foldase/ isomerase precursor | 11.8 | 0.6 | Membrane-associated |
| SAUSA300_0010 | *SAUSA300_0010* | Putative membrane protein | -10.3 | 1.6 | Membrane-associated* |
| SAUSA300_0274 | *SAUSA300_0274* | Putative membrane protein | -11.5 | 4.6 | Membrane-associated* |
| SAUSA300_1739 | *SAUSA300_1739* | Putative membrane protein | -15.2 | 1.4 | Membrane-associated* |
| SAUSA300_1740 | *SAUSA300_1740* | Uncharacterized protein | -15.9 | 2.0 | Membrane-associated* |
| SAUSA300_1093 | *pyrB* | Aspartate carbamoyltransferase | 149.6 | 16.7 | Metabolism - pyrimidine |
| SAUSA300_1094 | *pyrC* | Dihydroorotase | 101.6 | 7.4 | Metabolism - pyrimidine |
| SAUSA300_1095 | *carA* | Carbamoyl-phosphate synthase small chain | 77.3 | 1.4 | Metabolism - amino acid; pyrimidine |
| SAUSA300_1096 | *carB* | Carbamoyl-phosphate synthase large chain | 59.4 | 4.5 | Metabolism - amino acid; pyrimidine |
| SAUSA300_1097 | *pyrF* | Orotidine 5'-phosphate decarboxylase | 41.6 | 6.7 | Metabolism - pyrimidine |
| SAUSA300_1098 | *pyrE* | Orotate phosphoribosyltransferase | 41.2 | 4.1 | Metabolism - pyrimidine |
| SAUSA300_0885 | *fabH* | 3-oxoacyl-[acyl-carrier-protein] synthase 3 | 33.9 | 8.5 | Metabolism - lipid |
| SAUSA300_0355 | *SAUSA300_0355* | Acetyl-CoA C-acetyltransferase | 13.6 | 2.1 | Metabolism - *general* |
| SAUSA300_2526 | *pyrD* | Dihydroorotate dehydrogenase | 12.1 | 0.3 | Metabolism - pyrimidine |
| SAUSA300_2345 | *nirD* | Nitrite reductase (NADH) small subunit | -10.5 | 0.7 | Metabolism - nitrite |
| SAUSA300_0151 | *adhE* | Aldehyde-alcohol dehydrogenase | -10.6 | 0.5 | Metabolism - carbon |
| SAUSA300_0594 | *adh* | Alcohol dehydrogenase | -10.9 | 0.3 | Metabolism - carbon |
| SAUSA300_2500 | *crtQ* | 4,4'-diaponeurosporenoate glycosyltransferase | -11.0 | 0.4 | Metabolism - terpenoid and polyketide |
| SAUSA300_0357 | *metE* | 5-methyltetrahydropteroyltriglutamate--homocysteine methyltransferase | -11.7 | 0.2 | Metabolism - amino acid |
| SAUSA300_1711 | *putA* | Proline dehydrogenase | -11.7 | 1.0 | Metabolism - amino acid |
| SAUSA300_2014 | *ilvA* | Threonine dehydratase catabolic | -12.2 | 0.6 | Metabolism - amino acid |
| SAUSA300_2501 | *crtP* | Diapolycopene oxygenase | -12.5 | 1.8 | Metabolism - terpenoid and polyketide |
| SAUSA300_0229 | *fadX* | Putative acyl-CoA transferase | -12.5 | 4.0 | Metabolism - *general* |
| SAUSA300_2011 | *leuB* | 3-isopropylmalate dehydrogenase | -12.6 | 2.3 | Metabolism - amino acid; carbon |
| SAUSA300_2443 | *gntK* | Gluconokinase | -13.1 | 0.6 | Metabolism - carbon |
| SAUSA300_0711 | *SAUSA300_0711* | Putative lipid kinase | -14.4 | 0.8 | Metabolism - lipid |
| SAUSA300_0446 | *gltD* | Glutamate synthase (NADPH/NADH) small chain | -15.0 | 0.4 | Metabolism - amino acid; carbon |
| SAUSA300_1291 | *SAUSA300_1291* | Hippurate hydrolase | -16.4 | 9.8 | Metabolism - *general* |
| SAUSA300_2006 | *ilvD* | Dihydroxy-acid dehydratase | -16.5 | 3.5 | Metabolism - amino acid |
| SAUSA300_0358 | *SAUSA300_0358* | Putative 5-methyltetrahydrofolate-homocysteine methyltransferase | -17.0 | 2.4 | Metabolism - amino acid; carbon |
| SAUSA300_0445 | *gltB* | Glutamate synthase, large subunit | -18.3 | 2.2 | Metabolism - carbon |
| SAUSA300_2013 | *leuD* | 3-isopropylmalate dehydratase, small subunit | -18.7 | 19.0 | Metabolism - amino acid |
| SAUSA300_2007 | *ilvB* | Acetolactate synthase I/II/III large subunit | -19.9 | 6.2 | Metabolism - amino acid |
| SAUSA300_1629 | *thrS* | Threonyl-tRNA synthetase | -21.5 | 2.0 | Metabolism - aminoacyl-tRNA |
| SAUSA300_2012 | *leuC* | 3-isopropylmalate dehydratase large subunit | -23.0 | 16.4 | Metabolism - amino acid |
| SAUSA300_0359 | *SAUSA300_0359* | Cystathionine beta-lyase | -30.4 | 11.6 | Metabolism - amino acid |
| SAUSA300_2010 | *leuA* | 2-isopropylmalate synthase | -30.7 | 7.5 | Metabolism - amino acid |
| SAUSA300_1014 | *pyc* | Pyruvate carboxylase | -44.5 | 5.1 | Metabolism - amino acid; carbon |
| SAUSA300_1226 | *SAUSA300_1226* | Homoserine dehydrogenase | -45.3 | 0.5 | Metabolism - amino acid |
| SAUSA300_0360 | *SAUSA300_0360* | Cystathionine gamma-synthase | -53.8 | 22.3 | Metabolism - amino acid |
| SAUSA300_1227 | *thrC* | Threonine synthase | -57.9 | 12.0 | Metabolism - amino acid |
| SAUSA300_1225 | *SAUSA300_1225* | Aspartokinase | -61.0 | 4.6 | Metabolism - amino acid |
| SAUSA300_1290 | *dapD* | Tetrahydrodipicolinate N-acetyltransferase | -61.2 | 28.7 | Metabolism - amino acid |
| SAUSA300_1289 | *dapB* | 4-hydroxy-tetrahydrodipicolinate reductase | -73.3 | 1.8 | Metabolism - amino acid |
| SAUSA300_1228 | *thrB* | Homoserine kinase | -76.3 | 18.8 | Metabolism - amino acid |
| SAUSA300_2009 | *ilvC* | Ketol-acid reductoisomerase (NADP(+)). | -112.0 | 80.9 | Metabolism - amino acid |
| SAUSA300_1288 | *dapA* | 4-hydroxy-tetrahydrodipicolinate synthase | -152.7 | 80.9 | Metabolism - amino acid |
| SAUSA300_1286 | *SAUSA300_1286* | aspartate kinase | -155.5 | 2.0 | Metabolism - amino acid; carbon |
| SAUSA300_1287 | *asd* | Aspartate-semialdehyde dehydrogenase | -186.2 | 111.7 | Metabolism - amino acid |
| SAUSA300_1265 | *trpC+* | Indole-3-glycerol phosphate synthase | -500 | - | Metabolism - amino acid |
| SAUSA300_2305 | *SAUSA300_2305* | Transposase, truncation | -11.0 | 6.3 | Other - transposase |
| SAUSA300_0343 | *SAUSA300_0343* | Acetyltransferase | -17.5 | 1.1 | Other - transferase |
| SAUSA300_2619 | *SAUSA300_2619* | Adenosyl-fluoride synthase | -32.7 | 9.8 | Other - transferase |
| SAUSA300_2459 | *SAUSA300_2459* | MarR family transcriptional regulator | -16.5 | 7.0 | Transcriptional regulator |
| SAUSA300_1457 | *malR* | Maltose operon transcriptional repressor | -17.1 | 3.0 | Transcriptional regulator |
| SAUSA300_1092 | *pyrP* | Uracil permease | 176.6 | 22.6 | Transport |
| SAUSA300_0432 | *SAUSA300_0432* | Sodium dependent transporter | 70.0 | 6.4 | Transport |
| SAUSA300_2133 | *SAUSA300_2133* | Transporter gate domain protein | 13.4 | 1.8 | Transport |
| SAUSA300_2313 | *SAUSA300_2313* | L-lactate permease | -10.9 | 0.5 | Transport |
| SAUSA300_1762 | *epiF* | lantibiotic transport system ATP-binding protein | -12.6 | 4.0 | Transport |
| SAUSA300_2105 | *mtlF* | PTS system, mannitol-specific IIB component | -13.1 | 1.0 | Transport |
| SAUSA300_0202 | *SAUSA300_0202* | Peptide/nickel transport system permease | -13.7 | 2.9 | Transport |
| SAUSA300_2145 | *SAUSA300_2145* | Glycine betaine transporter | -13.8 | 3.3 | Transport |
| SAUSA300_2417 | *SAUSA300_2417* | Aminobenzoyl-glutamate transport protein | -14.5 | 1.5 | Transport |
| SAUSA300_0203 | *SAUSA300_0203* | Peptide/nickel transport system substrate-binding protein | -14.7 | 1.6 | Transport |
| SAUSA300_2617 | *SAUSA300_2617* | Putative cobalt ABC transporter | -15.0 | 3.3 | Transport |
| SAUSA300_2442 | *gntP* | Gluconate permease | -15.0 | 3.7 | Transport |
| SAUSA300_2618 | *SAUSA300_2618* | Energy-coupling factor transport system | -17.2 | 7.1 | Transport |
| SAUSA300_2449 | *SAUSA300_2449* | Putative transporter | -19.7 | 2.6 | Transport |
| SAUSA300_0796 | *metN2* | ABC transporter, ATP-binding protein | -36.3 | 7.7 | Transport |
| SAUSA300_0888 | *oppC* | Oligopeptide transport system permease protein | -49.5 | 3.7 | Transport |
| SAUSA300_0435 | *metN1* | D-methionine transport system ATP-binding protein | -57.2 | 1.3 | Transport |
| SAUSA300_0887 | *oppB* | Oligopeptide transport system permease protein | -59.9 | 26.3 | Transport |
| SAUSA300_0437 | *SAUSA300_0437* | D-methionine transport system substrate-binding protein | -94.7 | 33.3 | Transport |
| SAUSA300_2306 | *hrtA* | Putative hemin import ATP-binding protein HrtA. | -99.4 | 112.4 | Transport |
| SAUSA300_0436 | *SAUSA300_0436* | D-methionine transport system permease protein | -138.3 | 20.8 | Transport |
| SAUSA300_0798 | *SAUSA300_0798* | D-methionine transport system permease protein | -145.7 | 70.4 | Transport |
| SAUSA300_0797 | *SAUSA300_0797* | D-methionine transport system permease protein | -156.3 | 136.1 | Transport |
| SAUSA300_0889 | *oppD* | Oligopeptide transport ATP-binding protein | -187.4 | 103.2 | Transport |
| SAUSA300_2307 | *hrtB* | Putative hemin transport system permease protein | -222.4 | 288.9 | Transport |
| SAUSA300_0890 | *oppF* | Oligopeptide transport ATP-binding protein | -358.0 | 151.7 | Transport |
| SAUSA300_0891 | *oppA* | Oligopeptide transport ATP-binding protein | -400.3 | 87.4 | Transport |
| SAUSA300_1382 | *lukS-PVL* | Leukotoxin | 54.9 | 6.0 | **Virulence factor - toxin** |
| SAUSA300_1381 | *lukF-PV* | Leukotoxin | 39.3 | 10.9 | **Virulence factor - toxin** |
| SAUSA300_1769 | *lukE* | Leukotoxin | 18.9 | 0.3 | **Virulence factor - toxin** |
| SAUSA300_2486 | *clpL* | ATP-dependent Clp protease ATP-binding subunit | -10.1 | 1.7 | **Virulence factor - protease** |
| SAUSA300_1327 | *ebh* | Extracellular matrix-binding protein | -15.3 | 3.2 | **Virulence factor - surface protein** |
| SAUSA300_0547 | *sdrD* | Serine-aspartate repeat-containing protein | -26.7 | 10.4 | **Virulence factor - surface protein** |
| SAUSA300_1052 | *SAUSA300_1052* | Fibrinogen-binding protein | -36.3 | 38.6 | **Virulence factor - surface protein** |
| SAUSA300_1379 | *SAUSA300_1379+* | Putative lipoprotein | 175 | - | x Unknown |
| SAUSA300_1380 | *SAUSA300_1380* | Uncharacterized protein | 70.9 | 4.3 | x Unknown |
| SAUSA300_0561 | *SAUSA300_0561* | Uncharacterized protein | 33.1 | 22.2 | x Unknown |
| SAUSA300_2493 | *SAUSA300_2493* | Uncharacterized protein | 22.5 | 4.0 | x Unknown |
| SAUSA300_0815 | *ear* | Ear protein | 11.5 | 1.1 | x Unknown |
| SAUSA300_2525 | *SAUSA300_2525* | Uncharacterized protein | -10.8 | 0.6 | x Unknown |
| SAUSA300_1211 | *SAUSA300_1211* | Uncharacterized protein | -11.3 | 3.9 | x Unknown |
| SAUSA300_2143 | *SAUSA300_2143* | Uncharacterized protein | -11.4 | 1.8 | x Unknown |
| SAUSA300_1656 | *SAUSA300_1656* | Putative universal stress protein | -11.5 | 0.5 | x Unknown |
| SAUSA300_0011 | *SAUSA300_0011* | Uncharacterized protein | -13.1 | 5.8 | x Unknown |
| SAUSA300_1210 | *SAUSA300_1210* | Uncharacterized protein | -17.4 | 5.1 | x Unknown |
| SAUSA300_0929 | *SAUSA300_0929* | Uncharacterized protein | -21.7 | 7.9 | x Unknown |
| SAUSA300_1502 | *SAUSA300_1502+* | Putative lipoprotein | -500 | - | x Unknown |
|  | | |  |  |  |

^1^ Significantly induced genes whose relative fold change was greater than 10 in pyruvate grown cultures (YCP), relative to cultures grown in the absence of pyruvate (YC).

^*^ denotes uncharacterized protein with a predicted transmembrane domain

^+^ denotes genes with FC values arbitrarily set "max" fold change as gene is completely induced or repressed by pyruvate
