# Supplementary material for: Staphylococcus aureus Responds to the Central Metabolite Pyruvate To Regulate Virulence
Source: mBio. 2018 Jan 23;9(1):e02272-17. doi: 10.1128/mBio.02272-17 (PMC5784258; doi:10.1128/mBio.02272-17)
Supplement: TABLE S4 [file mbo001183696st4.docx]

**Supplemental Table 4: Metabolic pathways whose flux are significantly altered in the presence of pyruvate**.

| **Pathway** | | **E.C Number** | **Metabolic pathways altered in Pyruvate** | | | **Gene Association** | | **Fold Change \|YCP/YC\|** | | **Normalized Flux*** | | |
| --- | --- | --- | --- | --- | --- | --- | --- | --- | --- | --- | --- | --- |
|  |  |  |  |  |  |  |  |  |  | **YC** | | **YCP** |
| Alanine, aspartate and glutamate metabolism | | 4.3.2.1 | argininosuccinate lyase | | | SAUSA0863 | | 2.10 | | -1867 | | -3920 |
| Alanine, aspartate and glutamate metabolism | | 6.3.4.5 | argininosuccinate synthase | | | SAUSA0864 | | 2.10 | | -1867 | | -3920 |
| Alanine, aspartate and glutamate metabolism | | 2.6.1.1 | aspartate transaminase | | | SAUSA1916 | | 1.53 | | 2517 | | 3861 |
| Alanine, aspartate and glutamate metabolism | | 2.6.1.2 | D-alanine transaminase | | | SAUSA1696 | | 2.10 | | -2121 | | -1012 |
| Alanine, aspartate and glutamate metabolism | | 1.4.1.1 | L-alanine dehydrogenase | | | SAUSA1331 or SAUSA1655 | | 2.50 | | 1849 | | 740 |
| Alanine, aspartate and glutamate metabolism | | 1.4.1.2 | glutamate dehydrogenase (NAD) | | | SAUSA0861 | | 2.26 | | 7948 | | 3511 |
| Arginine and proline metabolism | | 3.5.3.6 | arginine deiminase | | | SAUSA2570 | | 4.84 | | -19251 | | -3975 |
| Arginine and proline metabolism | | 3.5.3.1 | arginase | | | SAUSA2114 | | 33.08 | | 31280 | | 946 |
| Arginine transport | | - | arginine/ornithine antiporter | | | SAUSA2568 | | 15.55 | | 13900 | | 894 |
| D-Alanine metabolism | | 5.1.1.1 | alanine racemase | | | SAUSA2027 or SAUSA1292 | | 2.48 | | -1856 | | -747 |
| D-Glutamine and D-glutamate metabolism | | 5.1.1.3 | glutamate racemase | | | SAUSA1049 | | 2.10 | | -2121 | | -1012 |
| Glycine, serine and threonine metabolism | | 4.3.1.19 | L-serine deaminase | | | SAUSA2469 and SAUSA2470 | | 2.51 | | -4296 | | -10786 |
| Glycine, serine and threonine metabolism | | 2.1.2.1 | glycine hydroxymethyltransferase | | | SAUSA2067 | | 2.48 | | 26582 | | 10739 |
| Glycine, serine and threonine metabolism | | 1.4.4.2 | glycine-cleavage complex | | | SAUSA1467 or SAUSA0996 | | 2.92 | | 31317 | | 10727 |
| Glycine metabolism | | 1.4.4.2 | glycine-cleavage complex | | | SAUSA1496 and SAUSA1497 | | 2.92 | | 31317 | | 10727 |
| Glycine metabolism | | 2.1.2.10 | glycine cleavage complex | | | SAUSA0791 and SAUSA1498 | | 2.92 | | 31317 | | 10727 |
| Pyruvate metabolism | | 1.1.1.38 | malic enzyme | | | SAUSA1648 | | 2.28 | | 4954 | | -11312 |
| Pyruvate metabolism | | 1.2.3.3 | pyruvate Oxidase | | | SAUSA2477 | | 13.50 | | 157493 | | 11666 |
| Pyruvate metabolism | | 2.3.1.54 | pyruvate formate lyase | | | SAUSA0220 | | 16.86 | | -157109 | | -9319 |
| Tryptophan metabolism | | 1.11.1.6 | catalase | | | SAUSA1232 | | 13.50 | | 157493 | | 11666 |
| Citrate cycle (TCA cycle) | | 4.2.1.2 | fumarase | | | SAUSA1801 | | 1.27 | | 4954 | | -3907 |
| Citrate cycle (TCA cycle) | | 1.3.5.1 | succinate dehyrdogenase | | | SAUSA1047 and SAUSA1048 and SAUSA1046 | | 1.39 | | 6809 | | 4902 |
| Glycolysis / Gluconeogenesis | | 4.2.1.11 | enolase | | | SAUSA0760 | | 4.47 | | -2515 | | -11240 |
| Glycolysis / Gluconeogenesis | | 1.2.1.59 | glyceraldehyde-3-phosphate dehydrogenase | | | SAUSA0756 or SAUSA1633 | | 4.47 | | -2515 | | -11240 |
| Glycolysis / Gluconeogenesis | | 2.7.2.3 | phosphoglycerate kinase | | | SAUSA0757 | | 4.47 | | 2515 | | 11240 |
| Glycolysis / Gluconeogenesis | | 5.4.2.1 | phosphoglycerate mutase | | | SAUSA0375 or SAUSA0759 or SAUSA2362 | | 4.47 | | 2515 | | 11240 |
| Glycolysis / Gluconeogenesis | | 4.1.1.49 | phosphoenolpyruvate carboxykinase | | | SAUSA1731 | | 4.47 | | 2517 | | 11241 |
| Glycolysis / Gluconeogenesis | | 5.3.1.1 | triose-phosphate isomerase | | | SAUSA0758 | | 5.38 | | -1993 | | -10718 |
| Glyoxylate and dicarboxylate metabolism | | 1.2.1.2 | formate dehydrogenase | | | SAUSA0179 and SAUSA2258 | | 8.18 | | -99228 | | 12129 |
| Purine metabolism | | 2.7.4.3 | adenylate kinase | | | SAUSA2183 | | 1.08 | | -1584 | | -1718 |
| Glycerophospholipid metabolism | | 1.1.1.8 | glycerol-3-phosphate dehydrogenase (NADP) | | | SAUSA1363 | | 8.43 | | -1478 | | -12454 |
| Oxidative phosphorylation | | 1.9.3.1 | cytochrome oxidase bd (ubiquinol-8: 2 protons) | | | SAUSA0986 and SAUSA0987 and SAUSA0963 and SAUSA0962 and SAUSA0961 and SAUSA0960 | | 1.68 | | 10077 | | 16908 |
| Oxidative phosphorylation | | 3.6.3.14 | ATP synthase (four protons for one ATP) | | | SAUSA2060 and SAUSA2064 and SAUSA2057 and SAUSA2058 and SAUSA2062 and SAUSA2059 and SAUSA2061 and SAUSA2063 and SA1912 | | 2.62 | | 5881 | | 15403 |
| Oxidative phosphorylation | | 1.6.99.3 | NADH dehydrogenase (ubiquinone-8 _ 3.5 protons) | | | SAUSA0425 and SAUSA0610 and SAUSA0841 and SAUSA0844 and SAUSA0855 and SAUSA1724 | | 2.78 | | 3268 | | 1175 |
| One carbon pool by folate | | 3.5.4.9 | methenyltetrahydrofolate cyclohydrolase | | | SAUSA1678 | | 2.70 | | 57898 | | 21465 |
| One carbon pool by folate | | 1.5.1.5 | methylenetetrahydrofolate dehydrogenase (NADP) | | | SAUSA0965 | | 2.70 | | 57898 | | 21465 |
| One carbon pool by folate | | 6.3.4.3 | formate-tetrahydrofolate ligase | | | SAUSA0965 | | 2.70 | | -57881 | | -21448 |
| Taurine and hypotaurine metabolism | | 2.3.1.8 | phosphotransacetylase | | | SAUSA0570 | | 16.16 | | -157493 | | -9748 |
|  | |  |  | | |  | |  | |  | |  |
|  |  | | |  |  | |  | |  | |  |  |

| **More Active in Pyruvate** |  |  |  |  |  |  |
| --- | --- | --- | --- | --- | --- | --- |
| **Less Active in Pyruvate** |  |  |  |  |  |  |
| **Direction of Reaction Flux changes** |  |  |  |  |  |  |

*Positive normalized flux indicates the forward reaction and negative flux indicates a reverse reaction. Normalized metabolic flux changes for *S. aureus* cultured with 2% pyruvate (YCP), were calculated relative to 0% pyruvate (YC). For normalized flux (*), positive values signify forward reactions whereas negative values signify reverse reactions. For simplicity, the absolute fold change is given and relative change in activity, irrespective of direction, are color coded (flux that become more active in pyruvate are colored in green and flux that become less active in pyruvate are colored in salmon). Flux that change direction in pyruvate are colored in orange.
